# Correction: National estimates from the Youth ’19 Rangatahi smart survey: A survey calibration approach

**DOI:** 10.1371/journal.pone.0253512

**Published:** 2021-06-17

**Authors:** The PLOS ONE Staff

## Notice of Republication

This article was republished on June 7, 2021, to include content edits to the article’s text that were requested by the authors prior to publication, but erroneously were not implemented during the typesetting process. The publisher apologizes for the errors. The republished article also provides an updated version of Fig 1, to correct for a typographical error in the “Students withdrawn by caregivers” box of the Sample Design.

Please download this article again to view the correct version. The originally published, uncorrected article and the republished, corrected articles are provided here for reference.

## Supporting information

S1 File Originally published, uncorrected article. (PDF)

S2 File Republished, corrected article. (PDF)
