# Supplementary figures and images for: National estimates from the Youth ’19 Rangatahi smart survey: A survey calibration approach
Source: PLoS One. 2021 May 14;16(5):e0251177. doi: 10.1371/journal.pone.0251177 (PMC8121344; doi:10.1371/journal.pone.0251177)

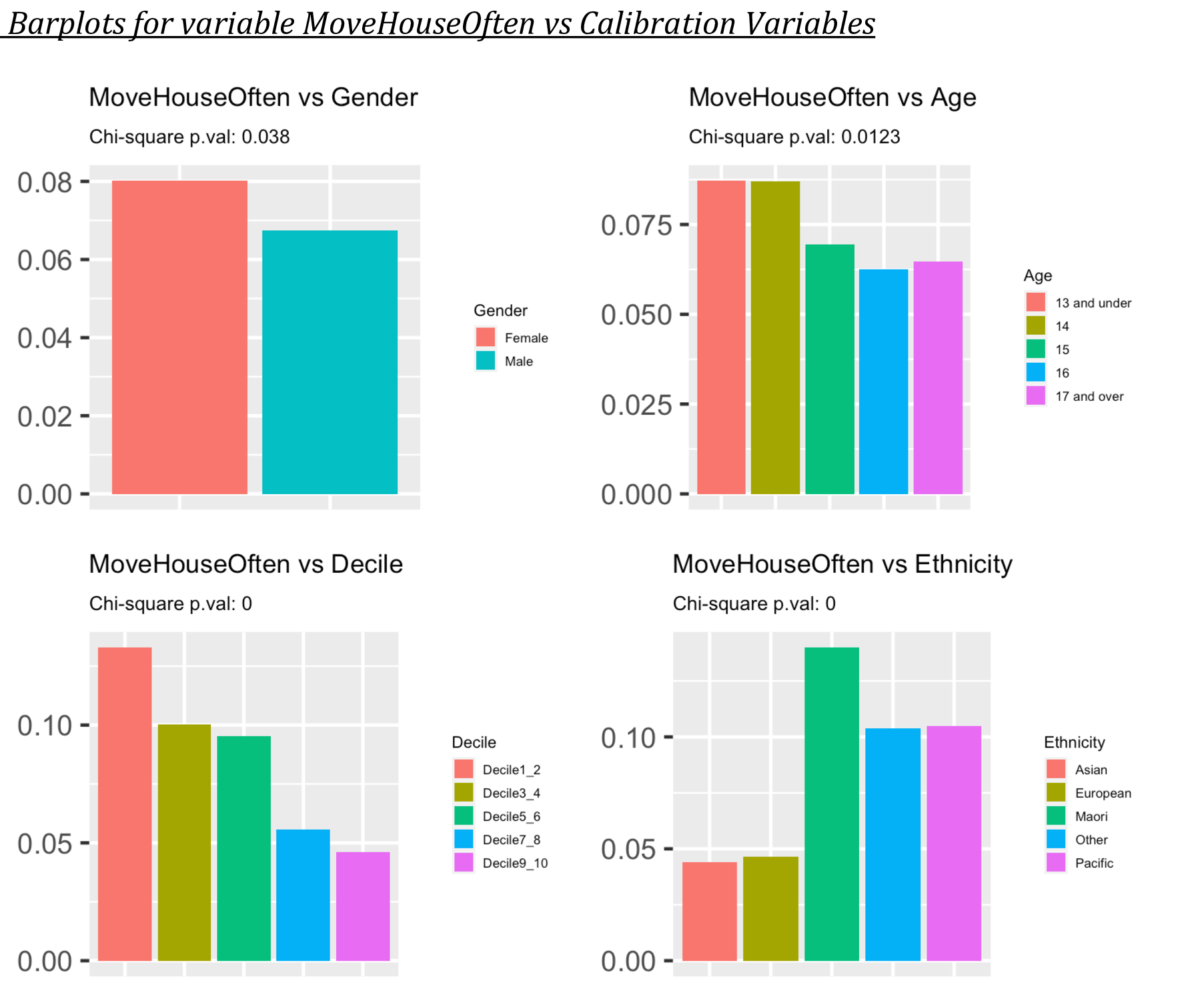

Supplement: S1 File — (ZIP) [file pone.0251177.s001.zip › Descriptive Stats File/Descriptive_Statistics1.tiff]

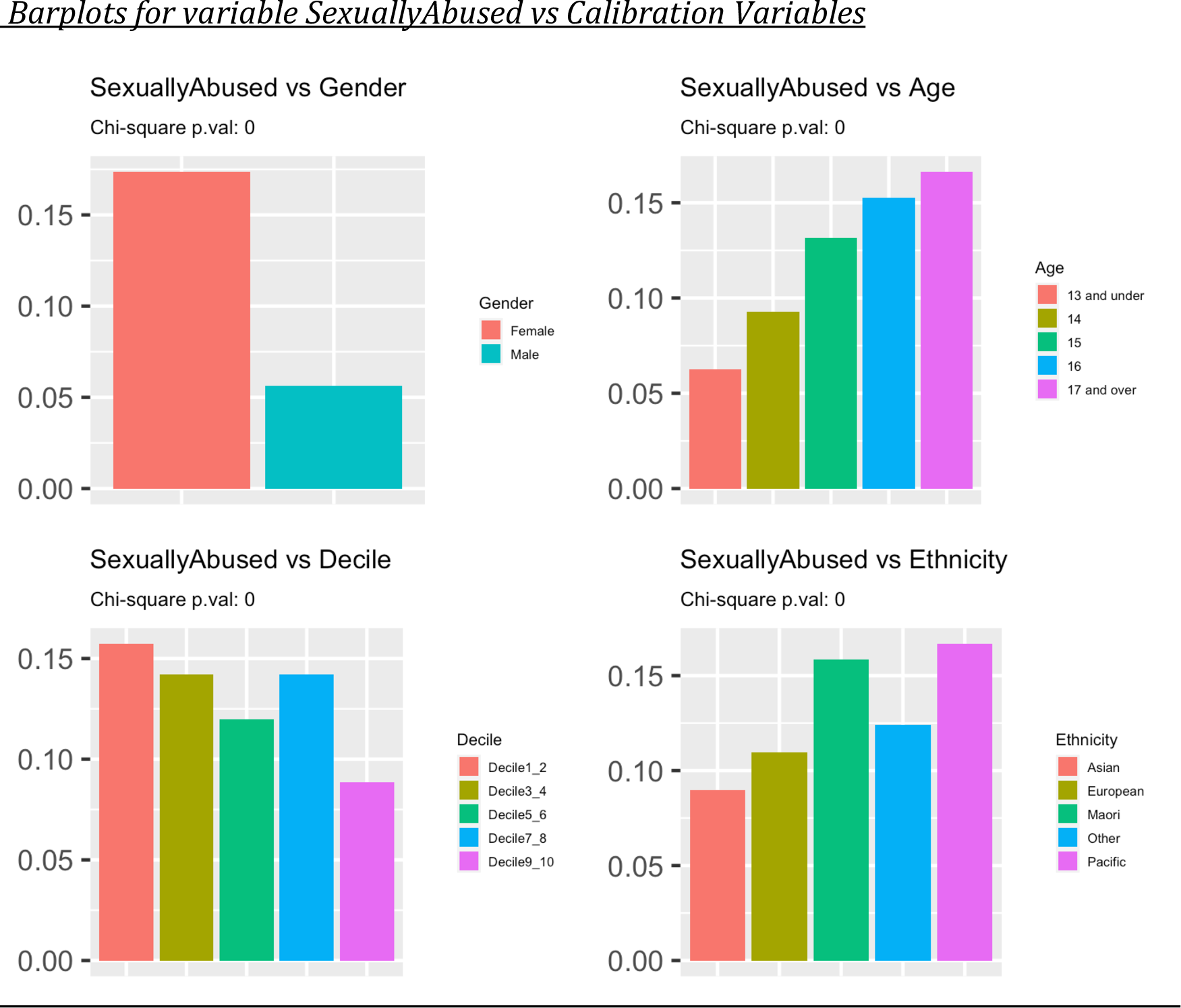

Supplement: S1 File — (ZIP) [file pone.0251177.s001.zip › Descriptive Stats File/Descriptive_Statistics10.tiff]

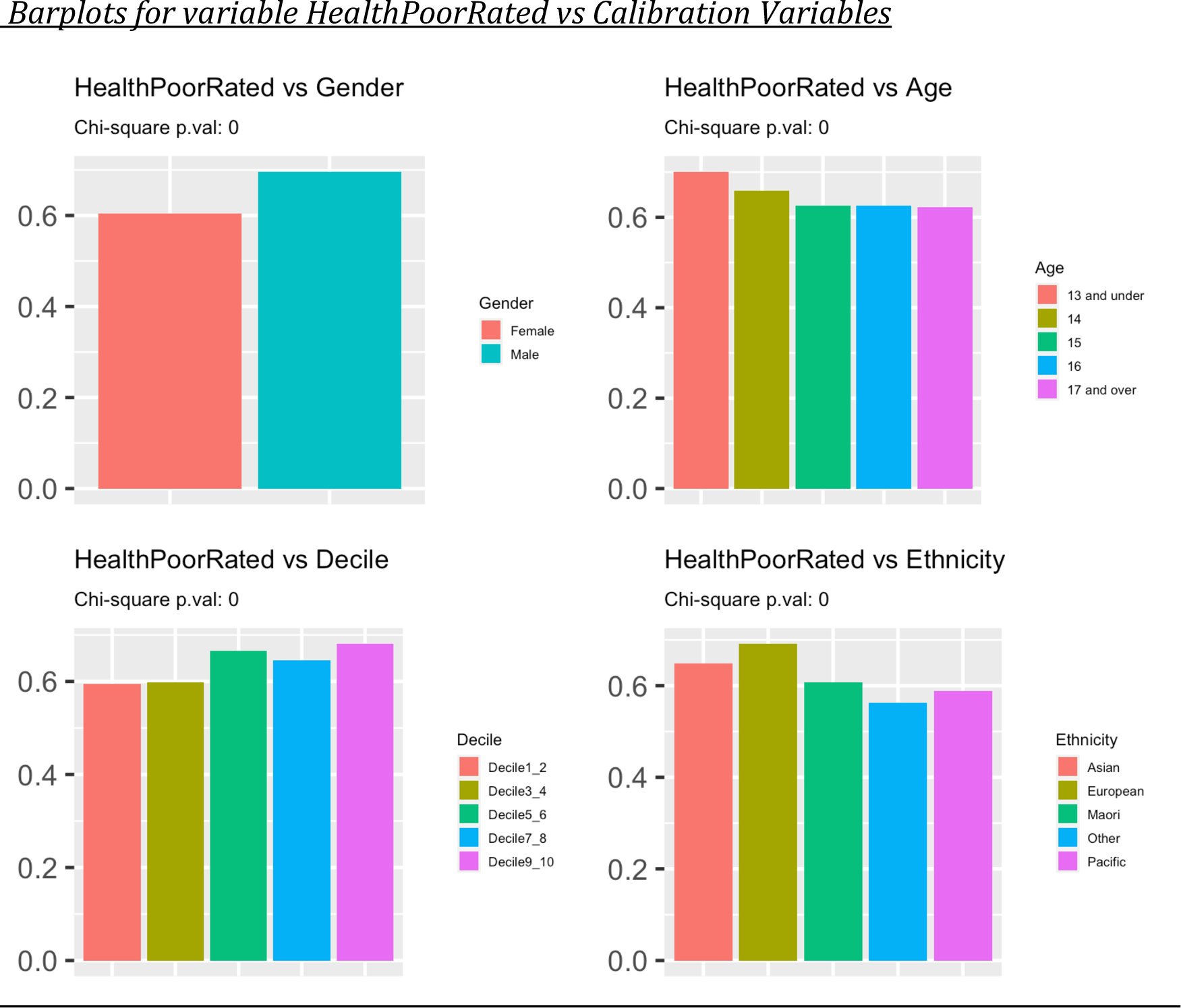

Supplement: S1 File — (ZIP) [file pone.0251177.s001.zip › Descriptive Stats File/Descriptive_Statistics11.tiff]

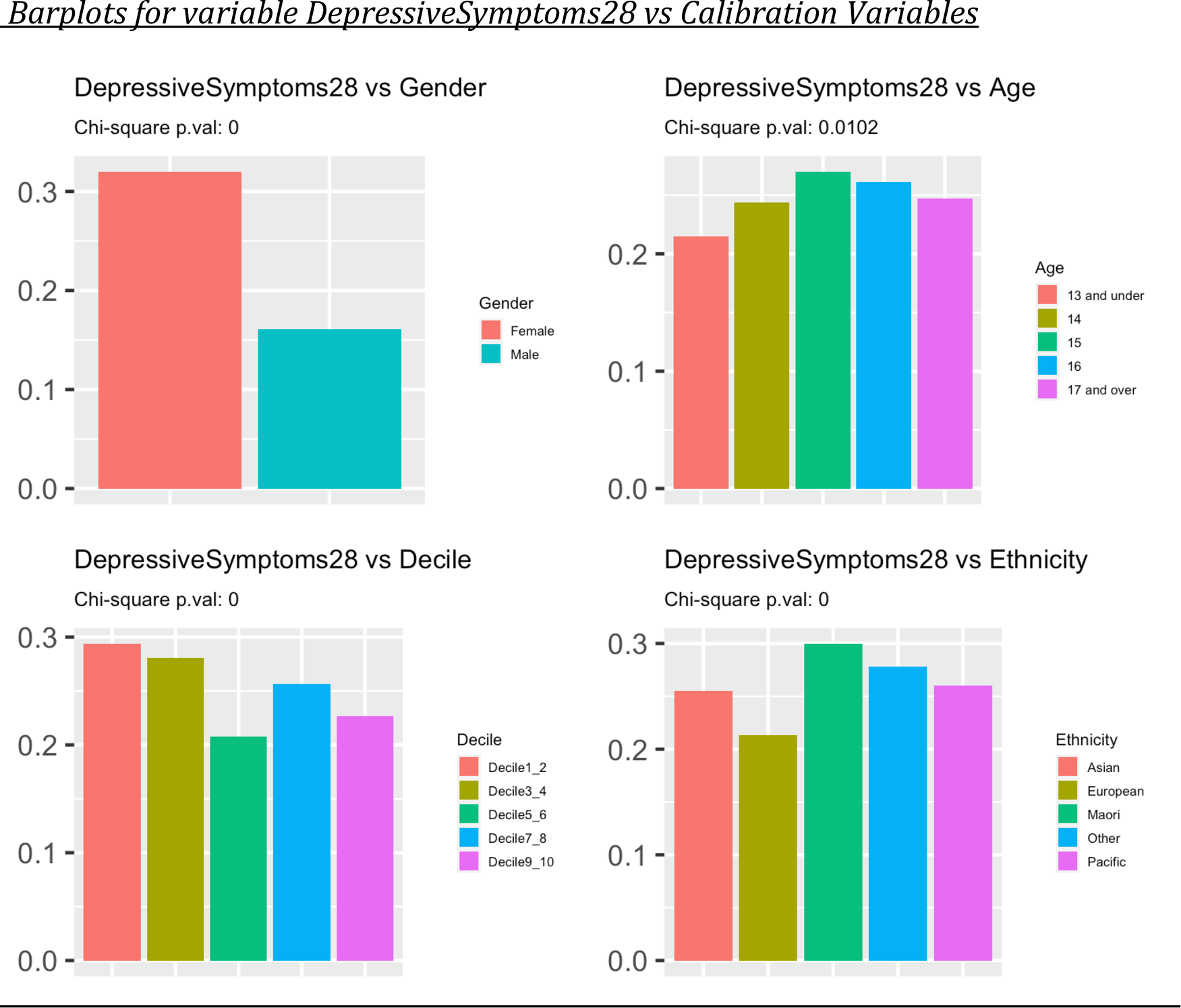

Supplement: S1 File — (ZIP) [file pone.0251177.s001.zip › Descriptive Stats File/Descriptive_Statistics12.tiff]

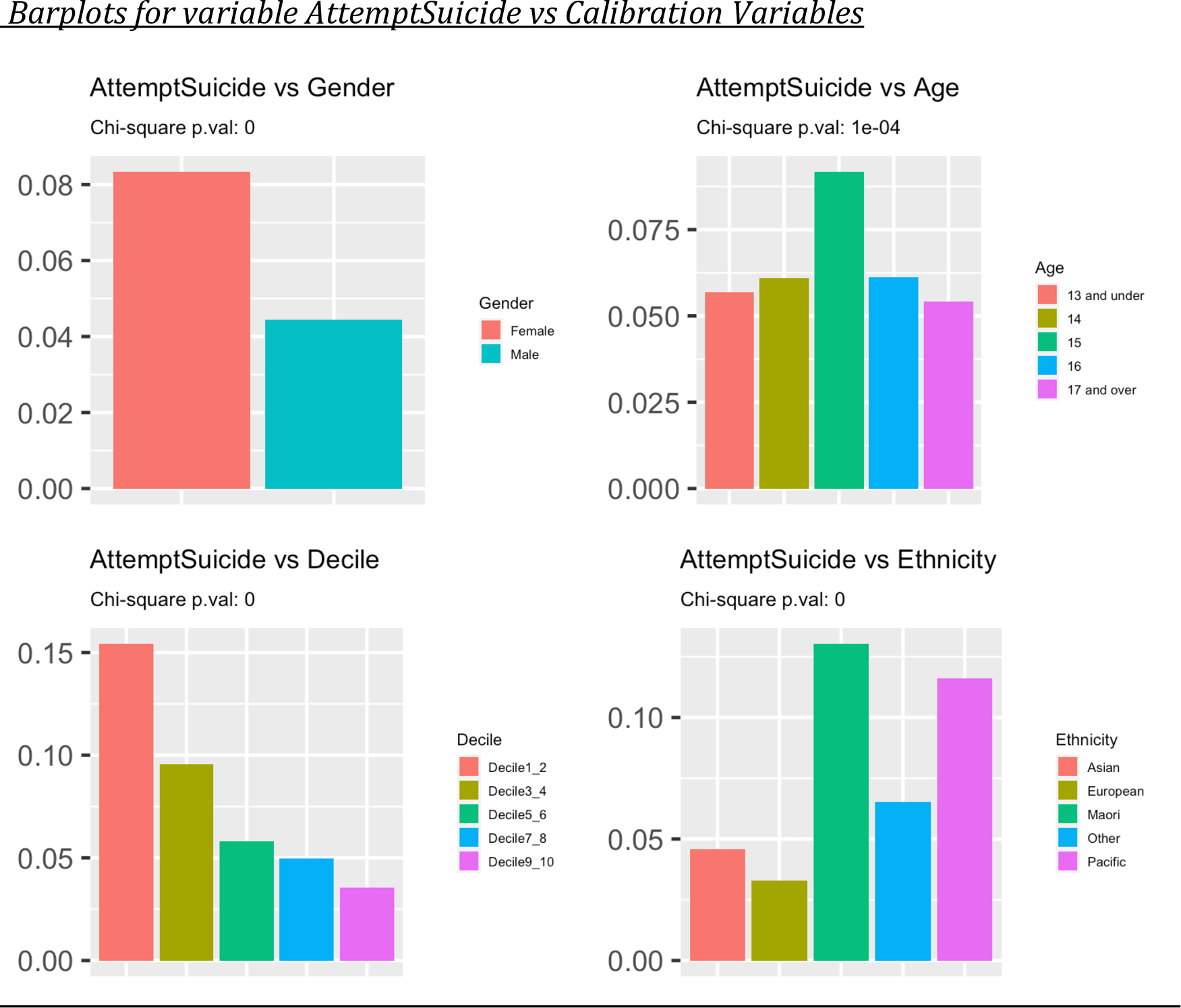

Supplement: S1 File — (ZIP) [file pone.0251177.s001.zip › Descriptive Stats File/Descriptive_Statistics13.tiff]

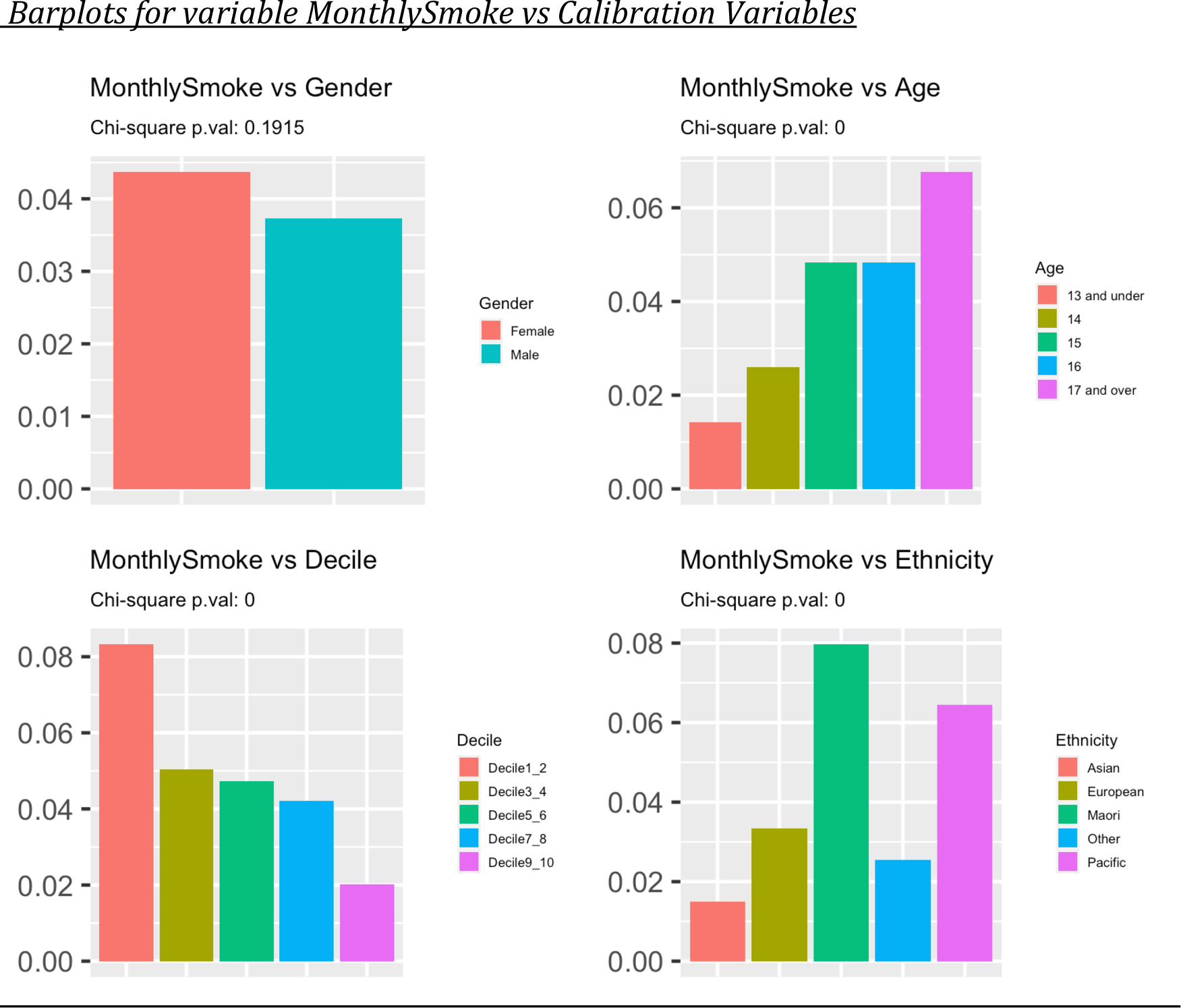

Supplement: S1 File — (ZIP) [file pone.0251177.s001.zip › Descriptive Stats File/Descriptive_Statistics14.tiff]

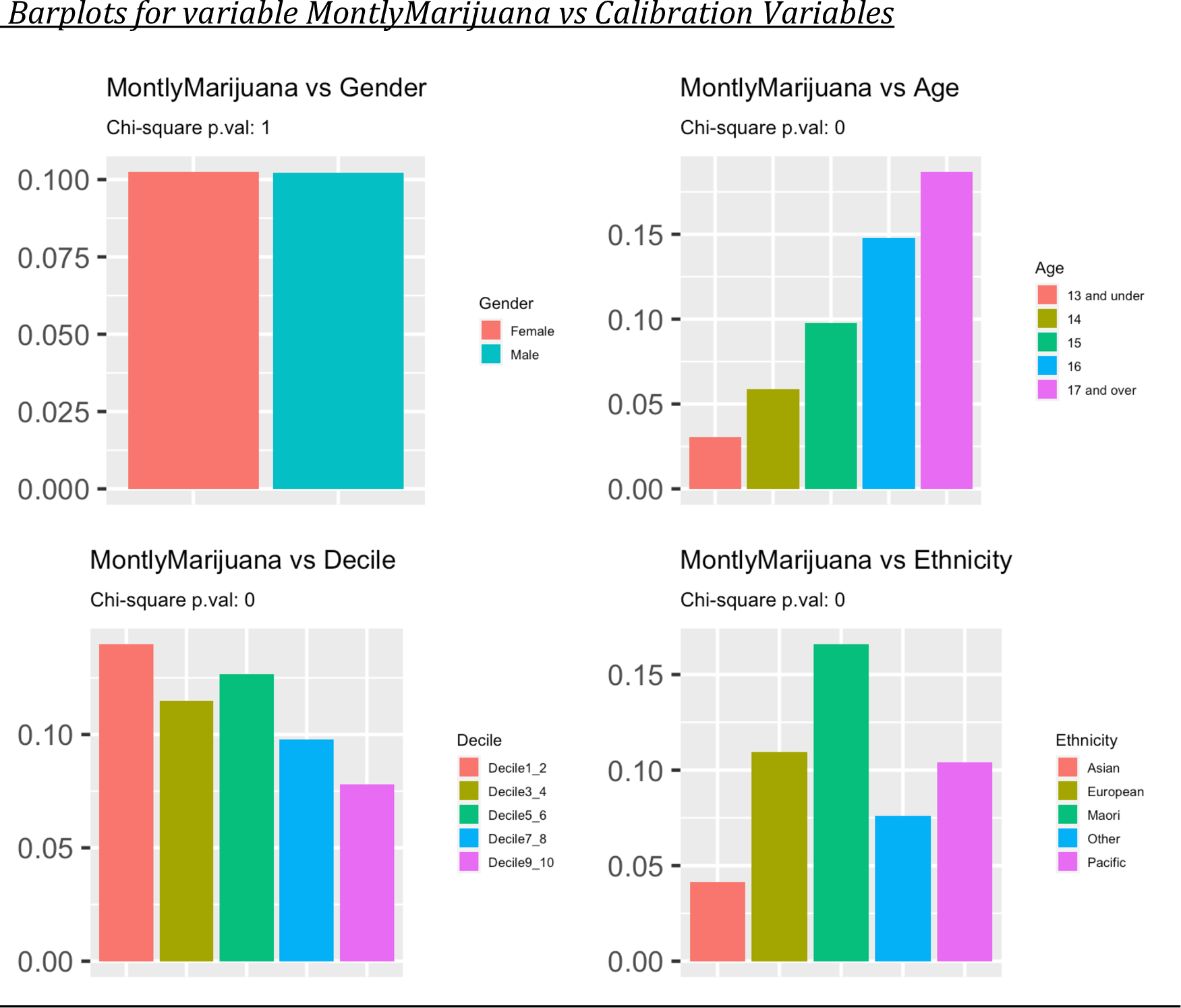

Supplement: S1 File — (ZIP) [file pone.0251177.s001.zip › Descriptive Stats File/Descriptive_Statistics15.tiff]

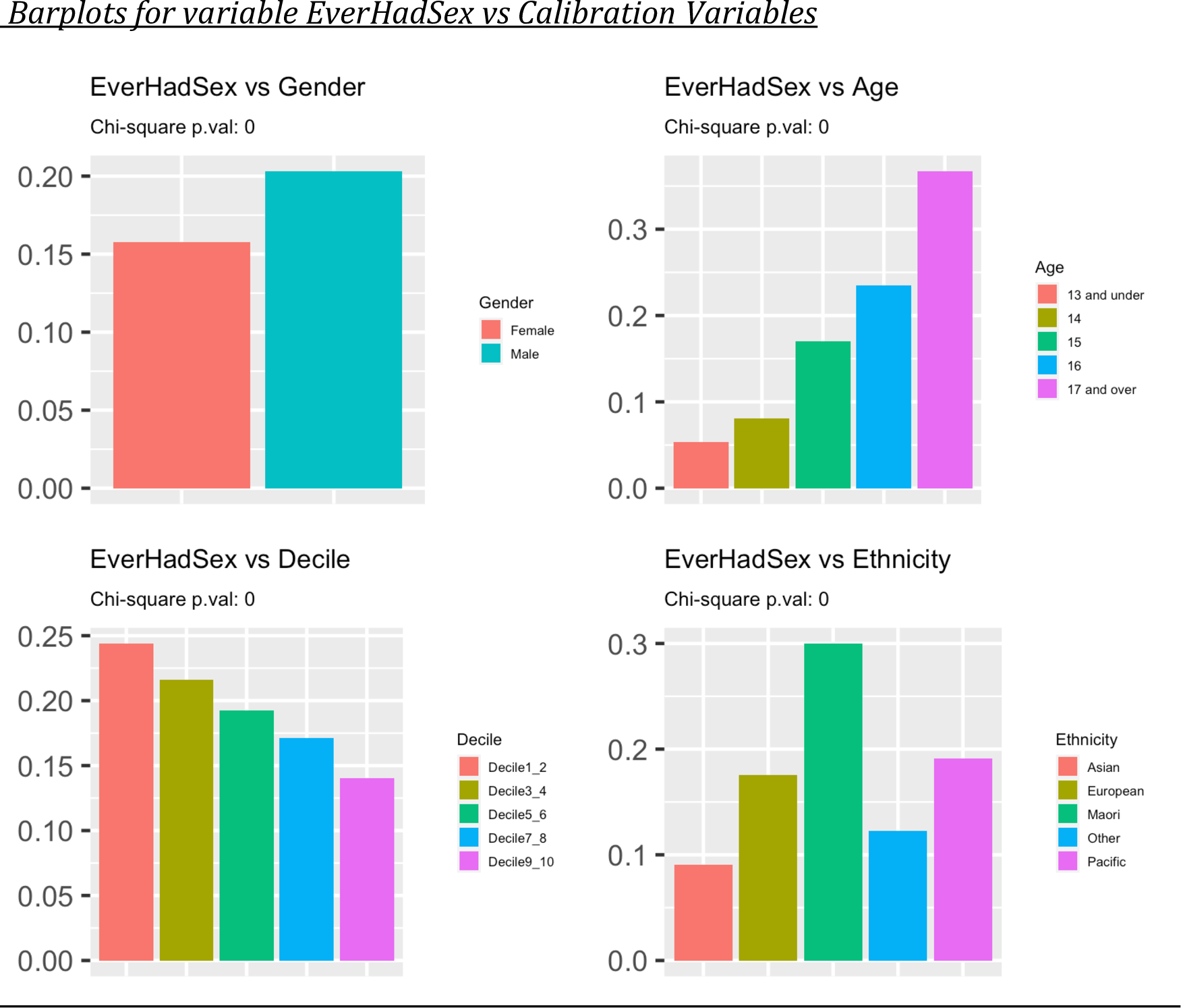

Supplement: S1 File — (ZIP) [file pone.0251177.s001.zip › Descriptive Stats File/Descriptive_Statistics16.tiff]

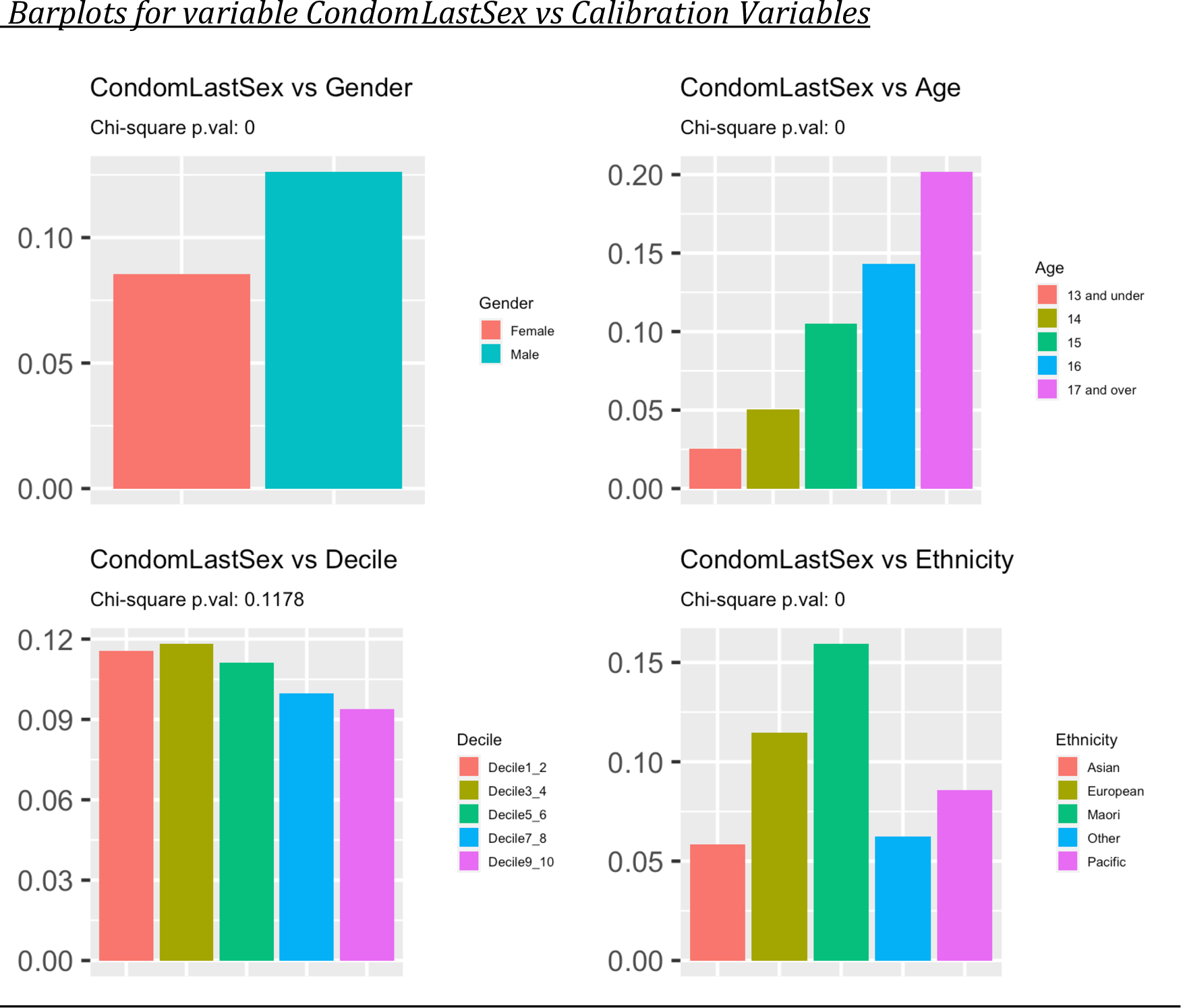

Supplement: S1 File — (ZIP) [file pone.0251177.s001.zip › Descriptive Stats File/Descriptive_Statistics17.tiff]

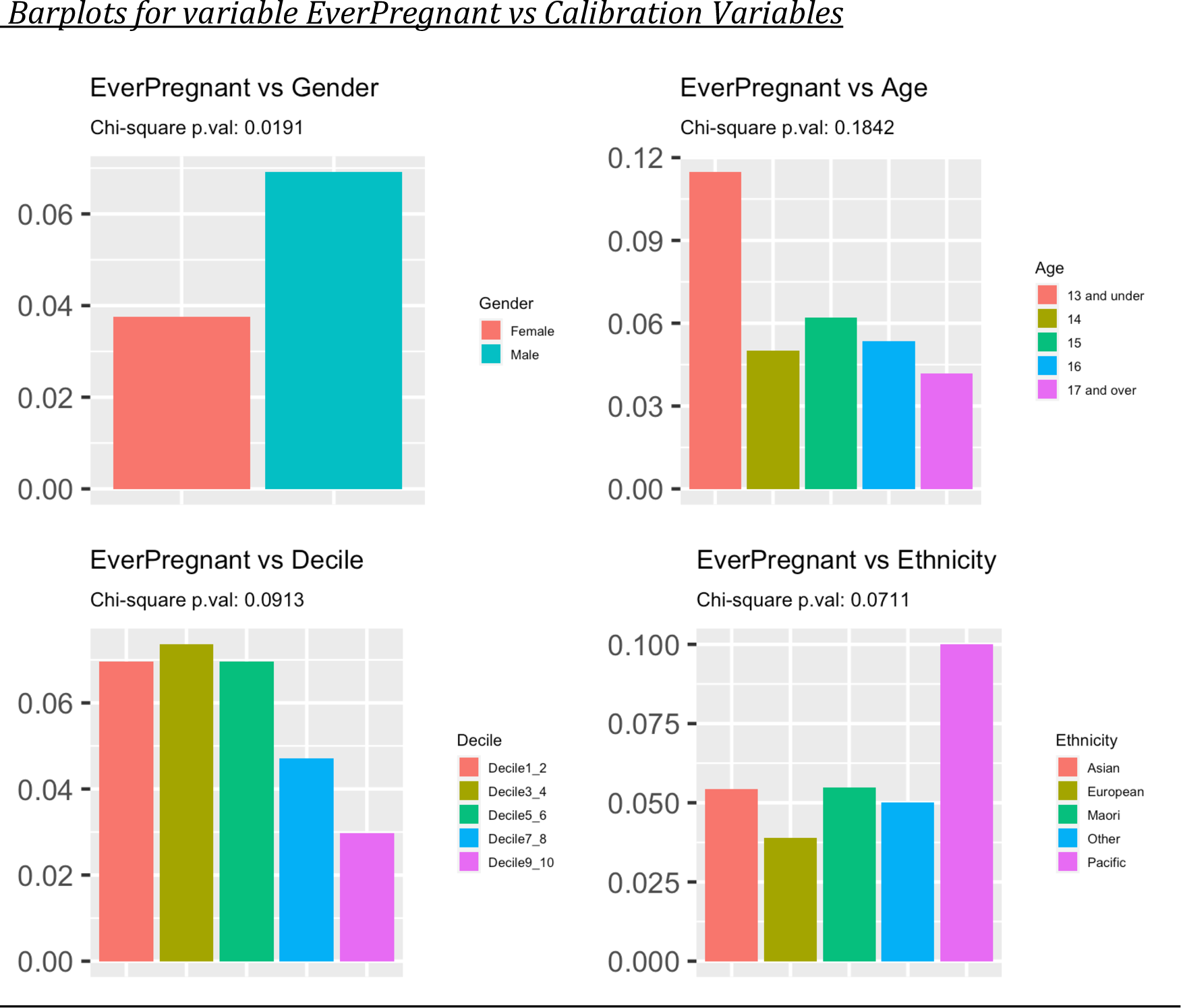

Supplement: S1 File — (ZIP) [file pone.0251177.s001.zip › Descriptive Stats File/Descriptive_Statistics18.tiff]

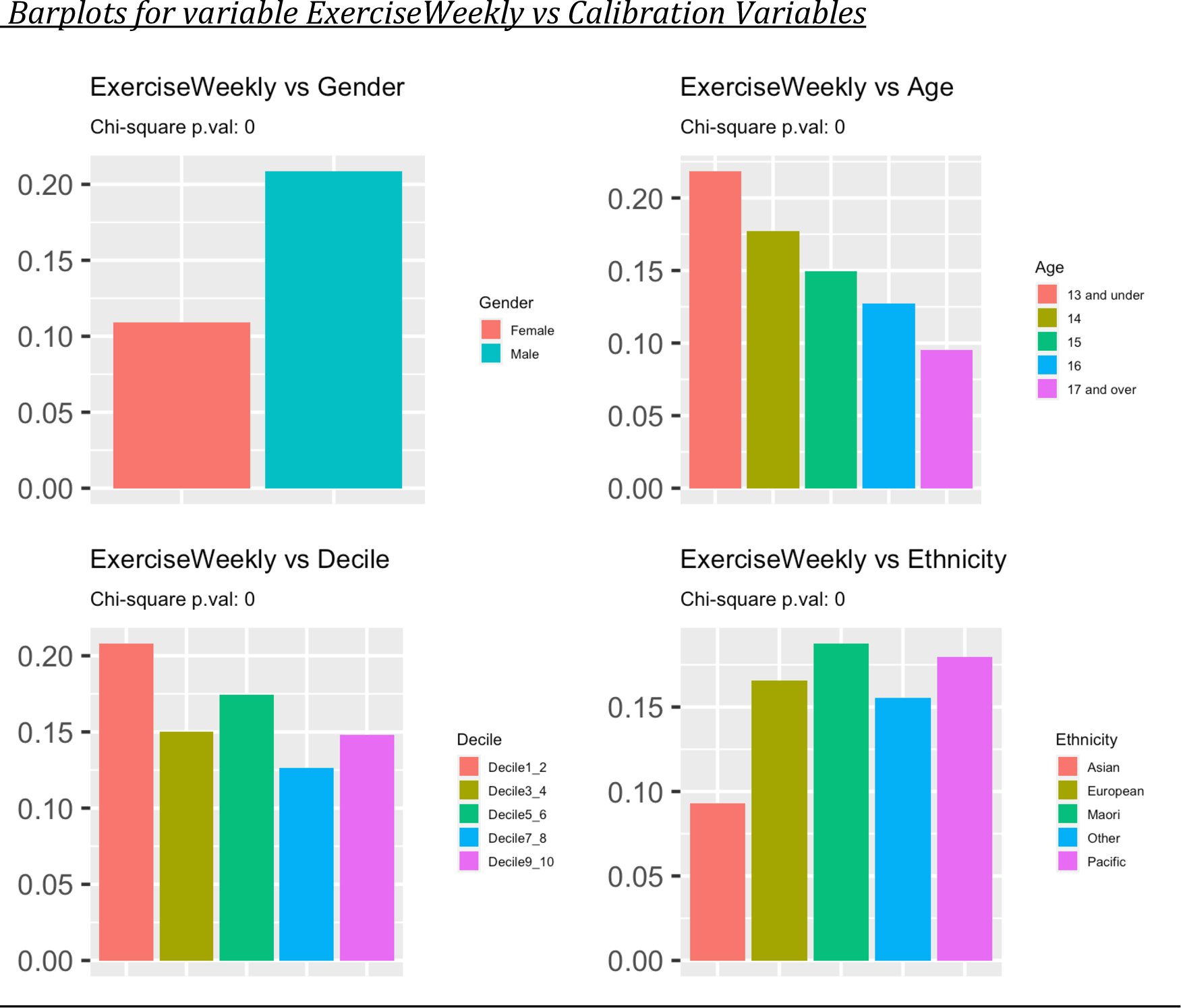

Supplement: S1 File — (ZIP) [file pone.0251177.s001.zip › Descriptive Stats File/Descriptive_Statistics19.tiff]

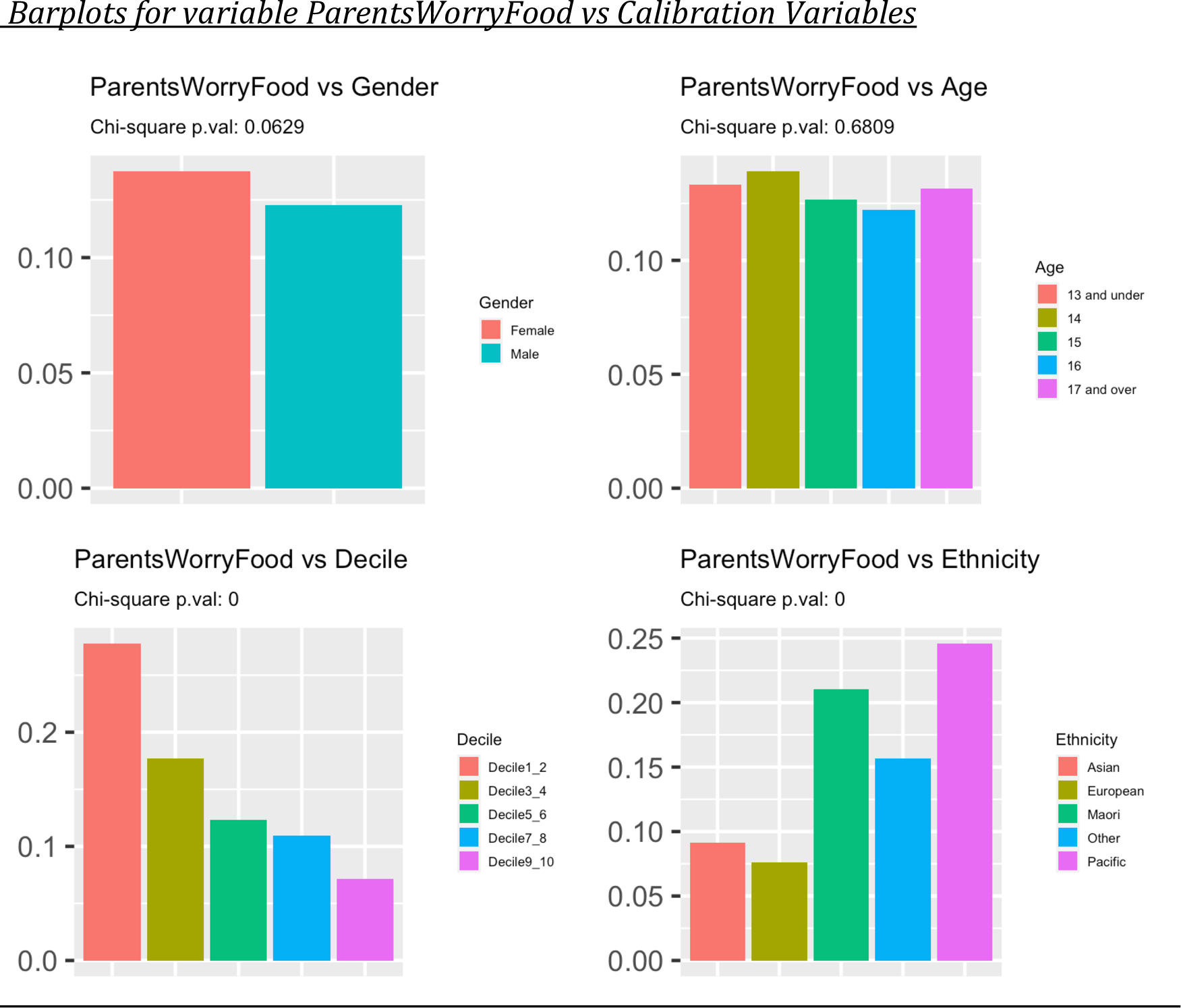

Supplement: S1 File — (ZIP) [file pone.0251177.s001.zip › Descriptive Stats File/Descriptive_Statistics2.tiff]

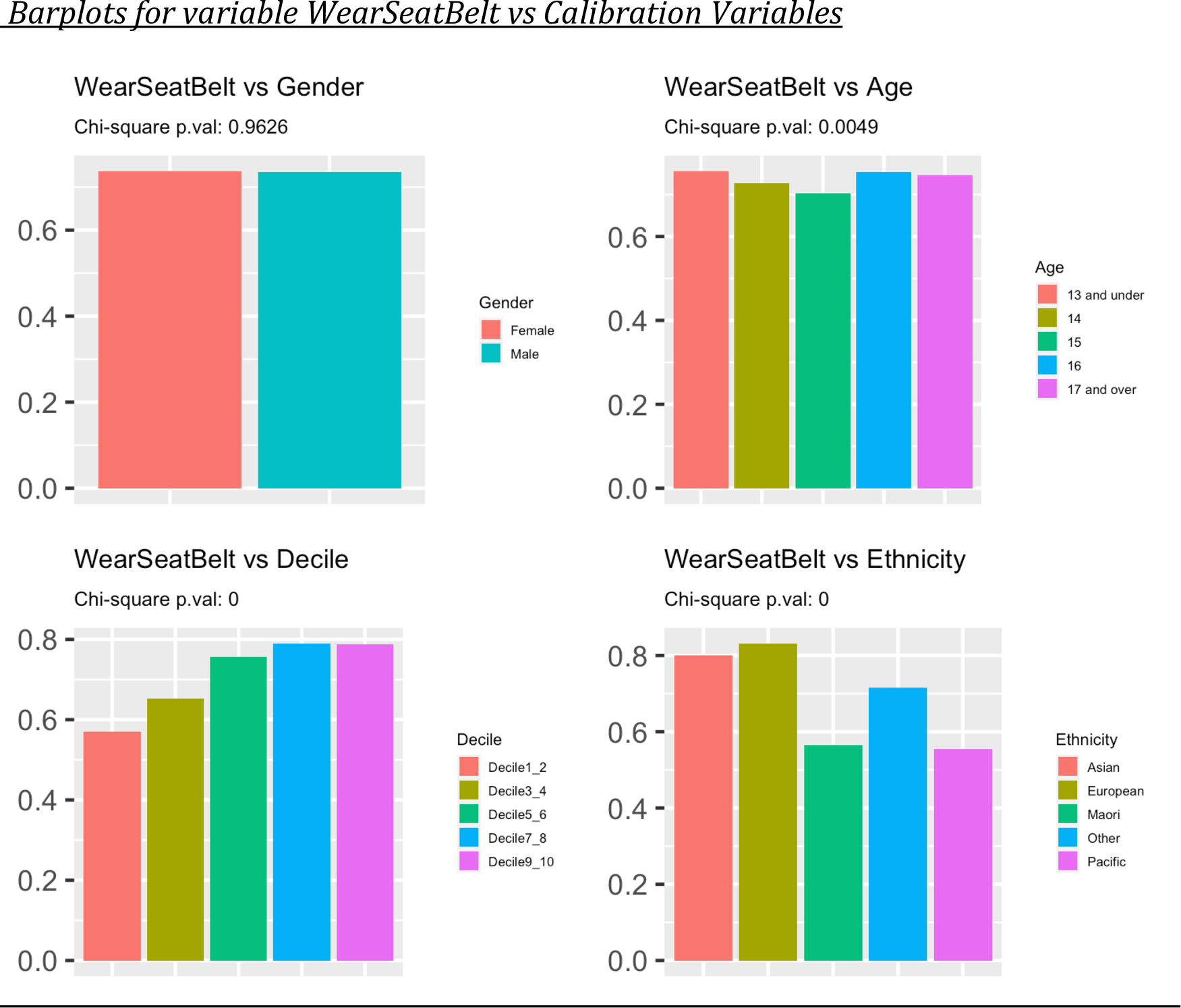

Supplement: S1 File — (ZIP) [file pone.0251177.s001.zip › Descriptive Stats File/Descriptive_Statistics20.tiff]

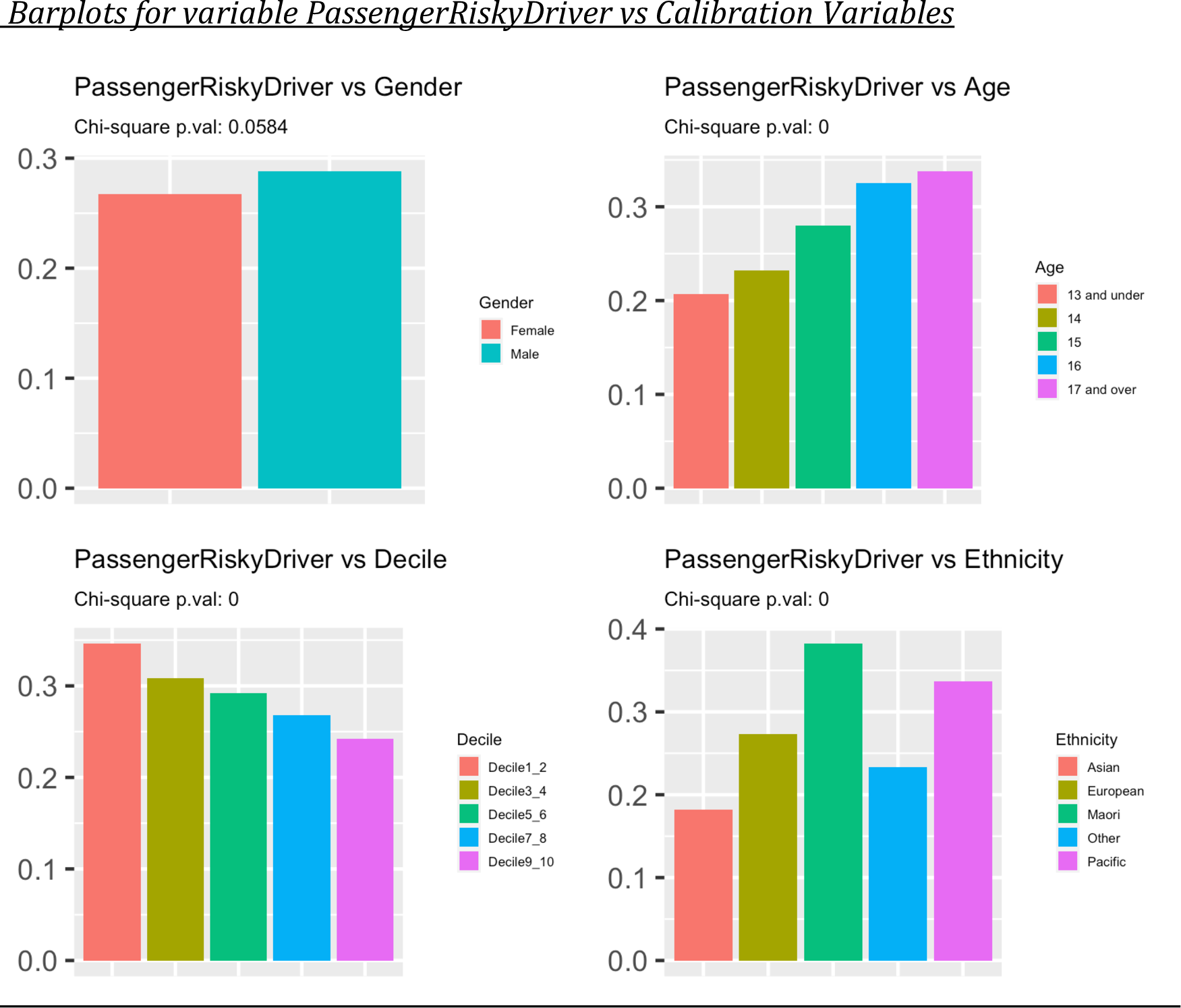

Supplement: S1 File — (ZIP) [file pone.0251177.s001.zip › Descriptive Stats File/Descriptive_Statistics21.tiff]

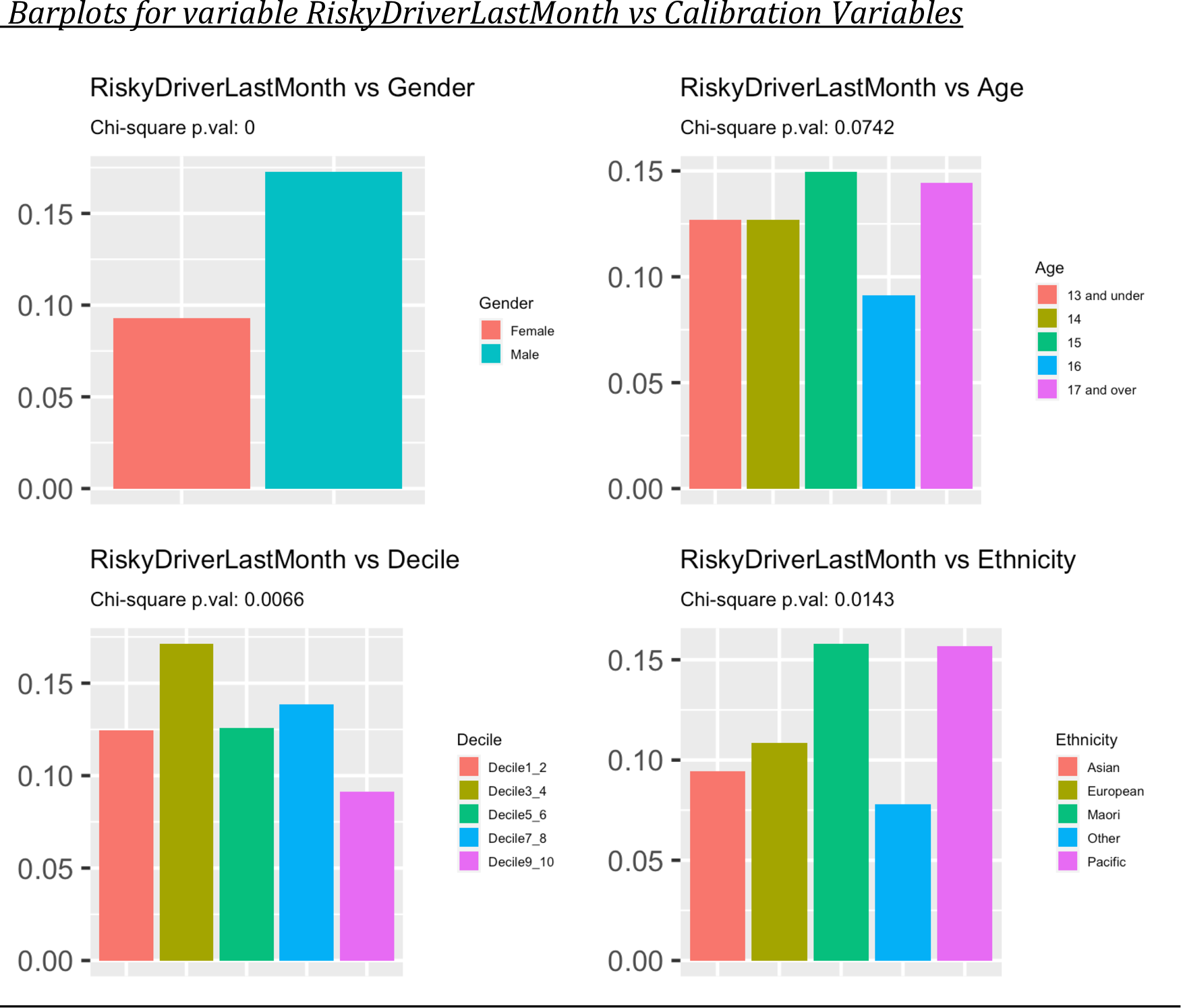

Supplement: S1 File — (ZIP) [file pone.0251177.s001.zip › Descriptive Stats File/Descriptive_Statistics22.tiff]

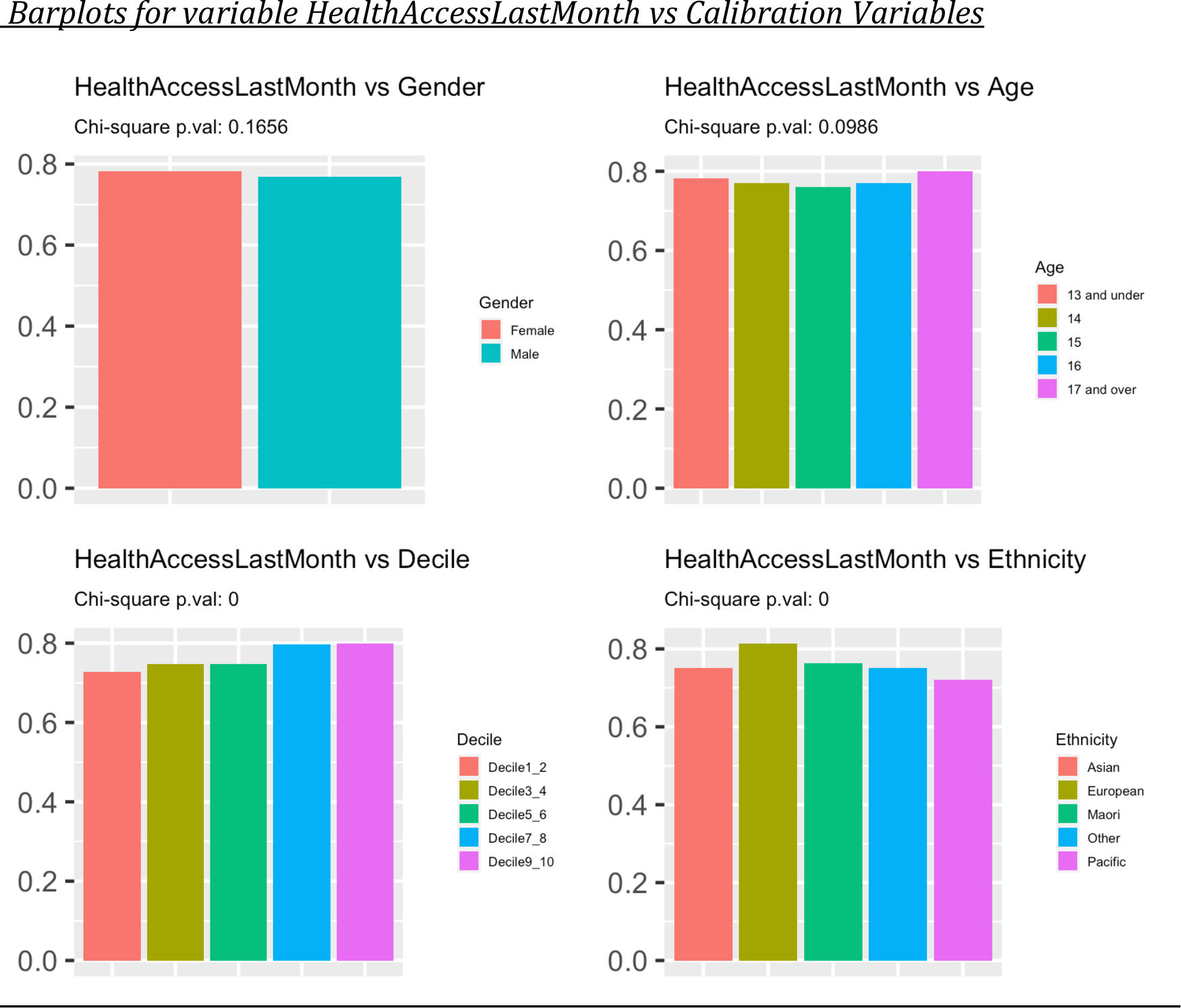

Supplement: S1 File — (ZIP) [file pone.0251177.s001.zip › Descriptive Stats File/Descriptive_Statistics23.tiff]

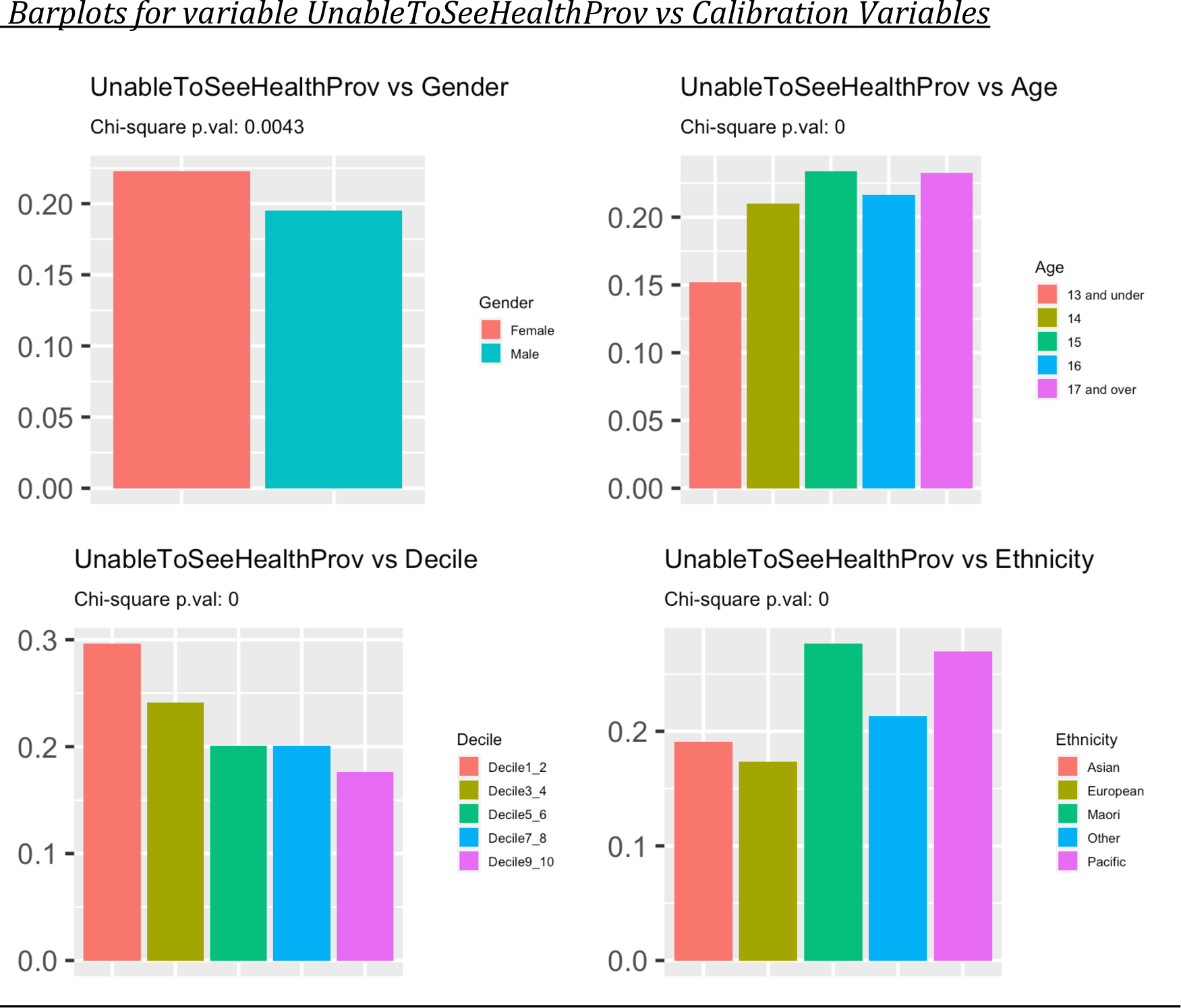

Supplement: S1 File — (ZIP) [file pone.0251177.s001.zip › Descriptive Stats File/Descriptive_Statistics24.tiff]

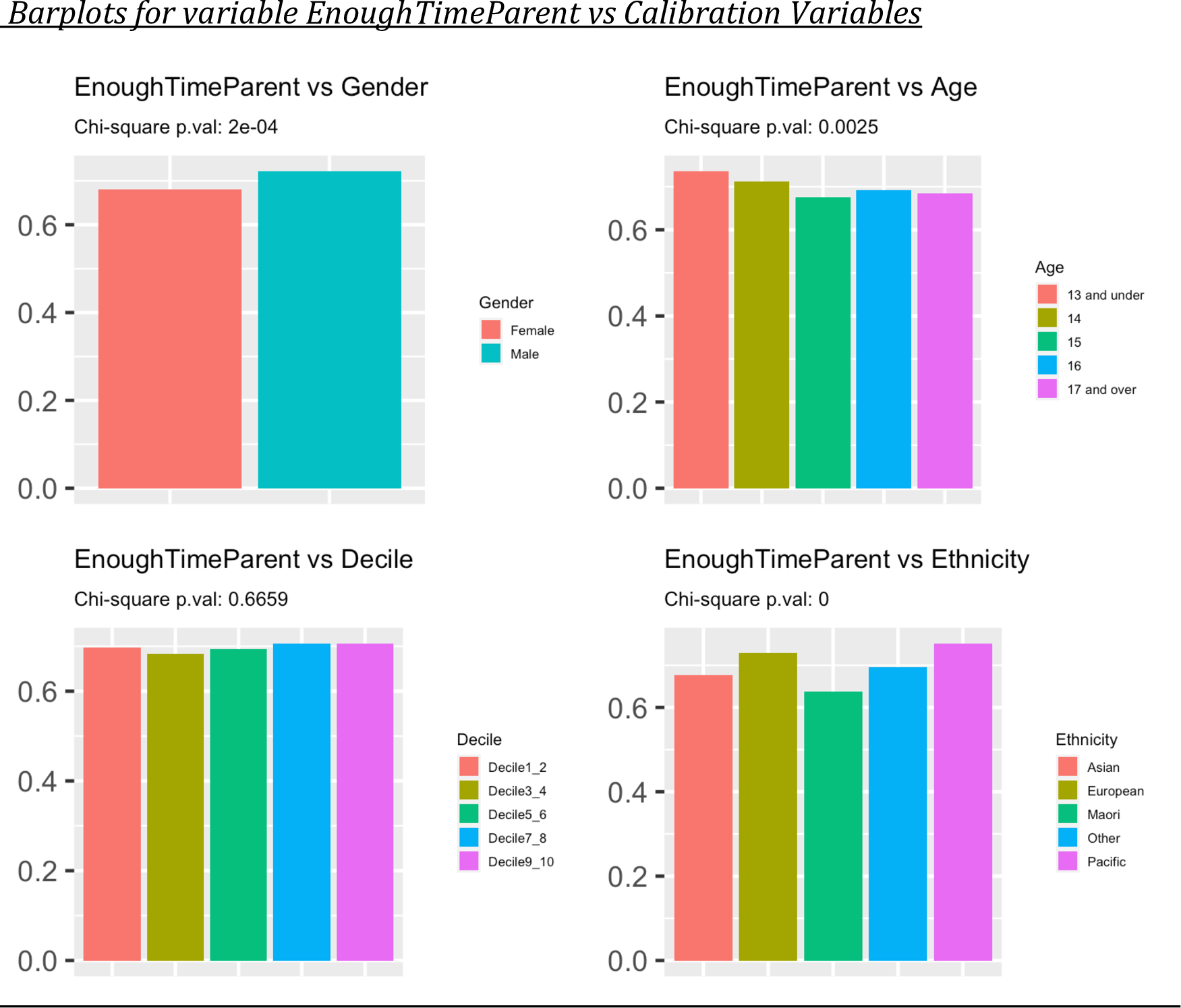

Supplement: S1 File — (ZIP) [file pone.0251177.s001.zip › Descriptive Stats File/Descriptive_Statistics3.tiff]

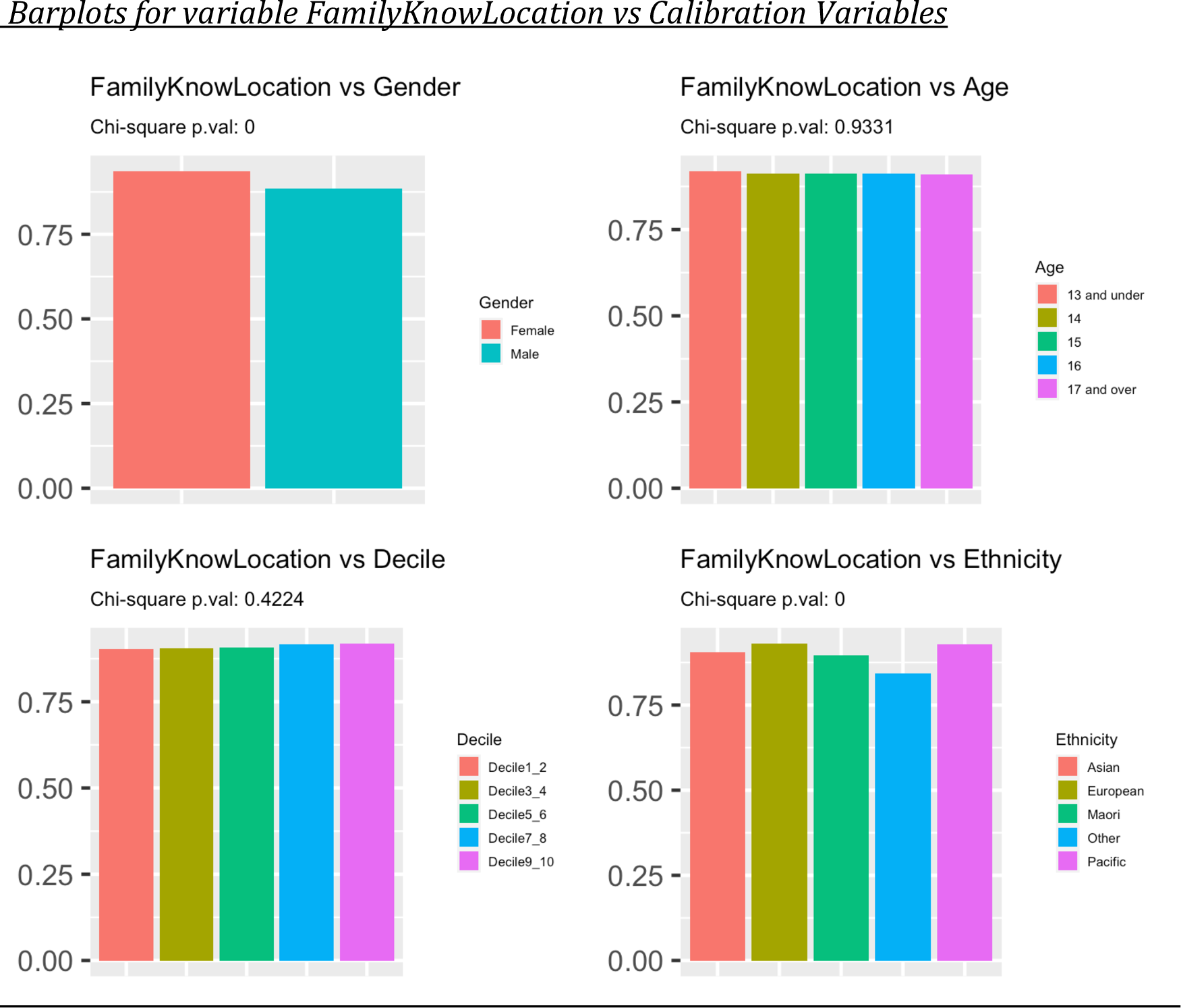

Supplement: S1 File — (ZIP) [file pone.0251177.s001.zip › Descriptive Stats File/Descriptive_Statistics4.tiff]

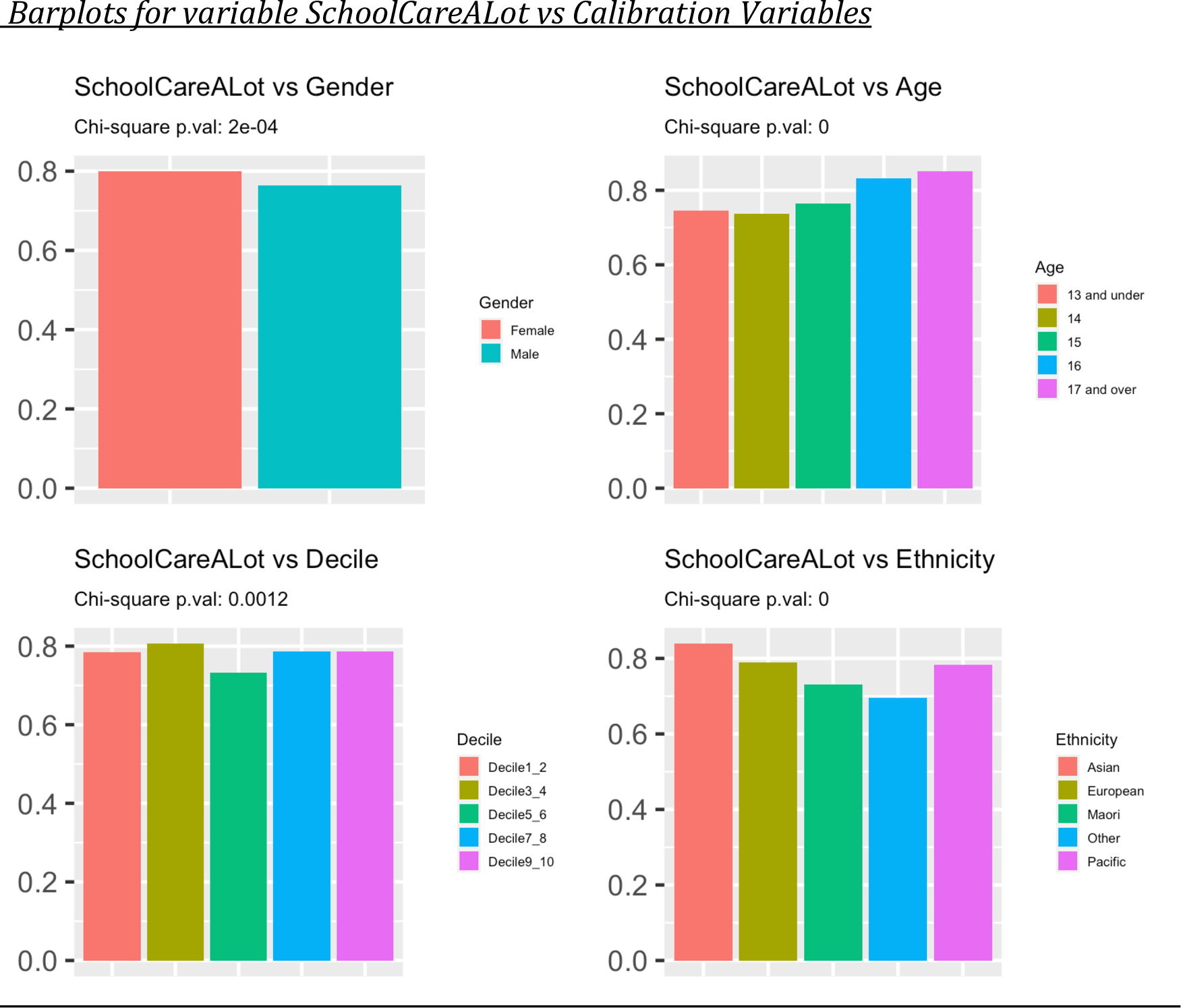

Supplement: S1 File — (ZIP) [file pone.0251177.s001.zip › Descriptive Stats File/Descriptive_Statistics5.tiff]

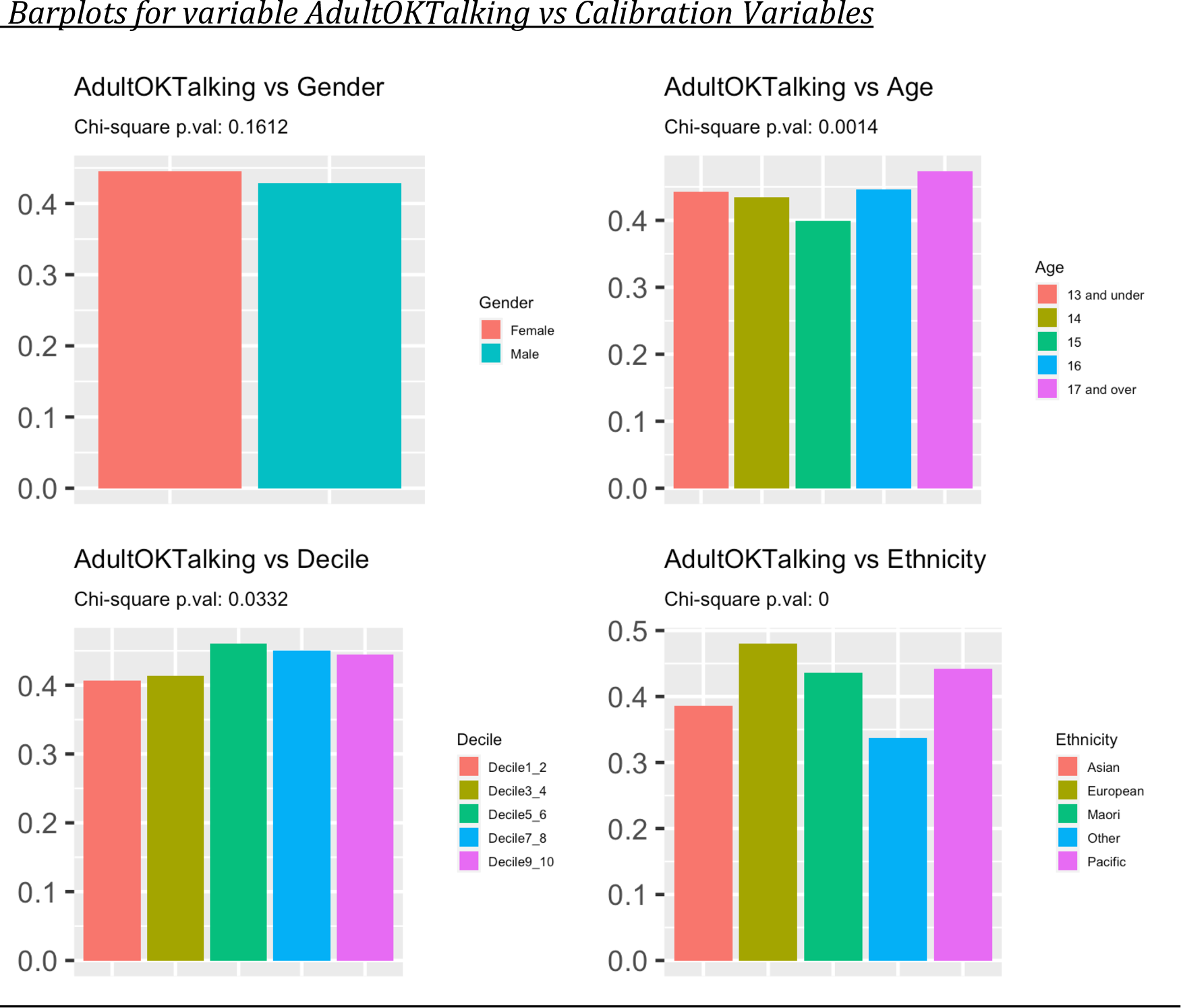

Supplement: S1 File — (ZIP) [file pone.0251177.s001.zip › Descriptive Stats File/Descriptive_Statistics6.tiff]

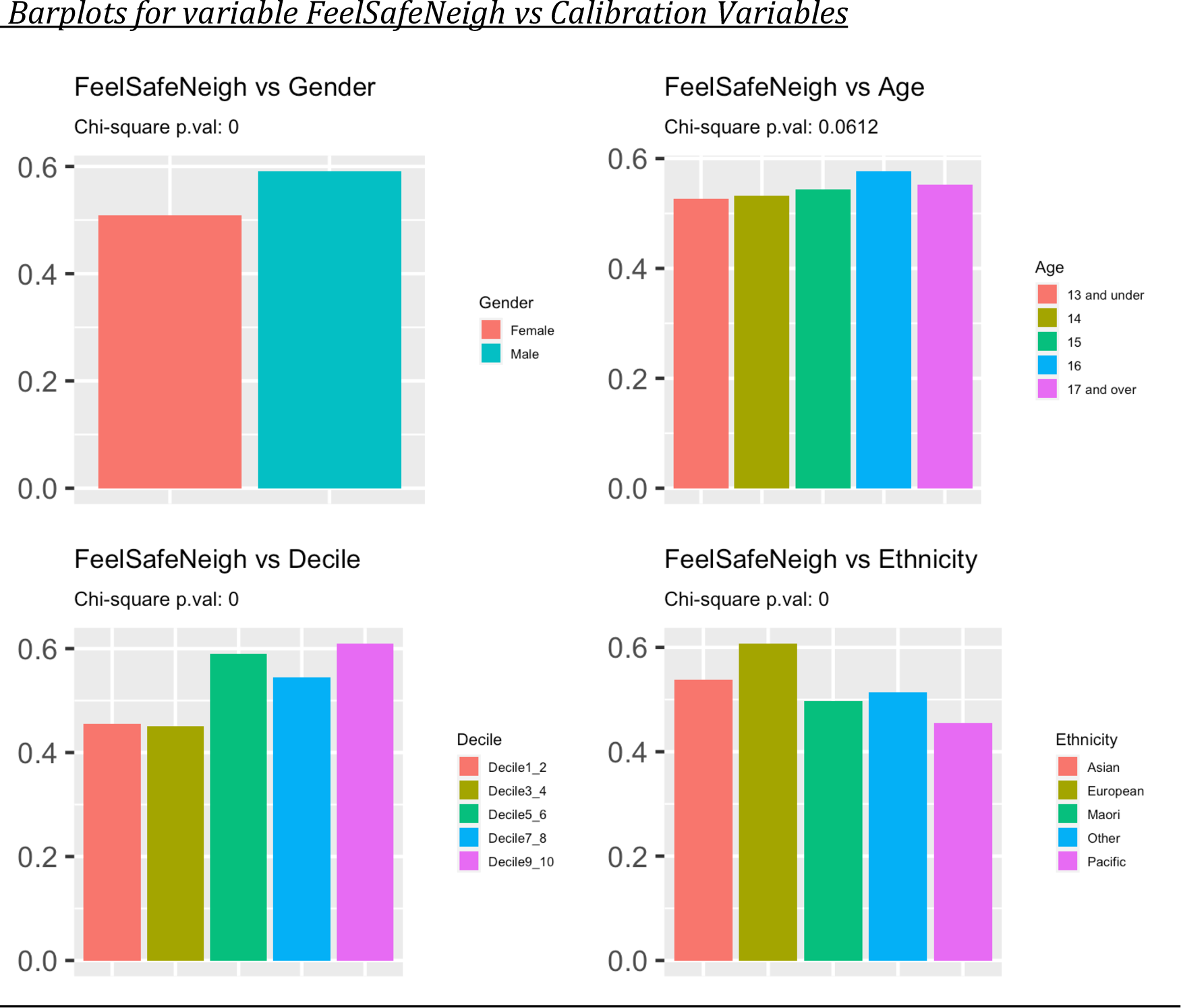

Supplement: S1 File — (ZIP) [file pone.0251177.s001.zip › Descriptive Stats File/Descriptive_Statistics7.tiff]

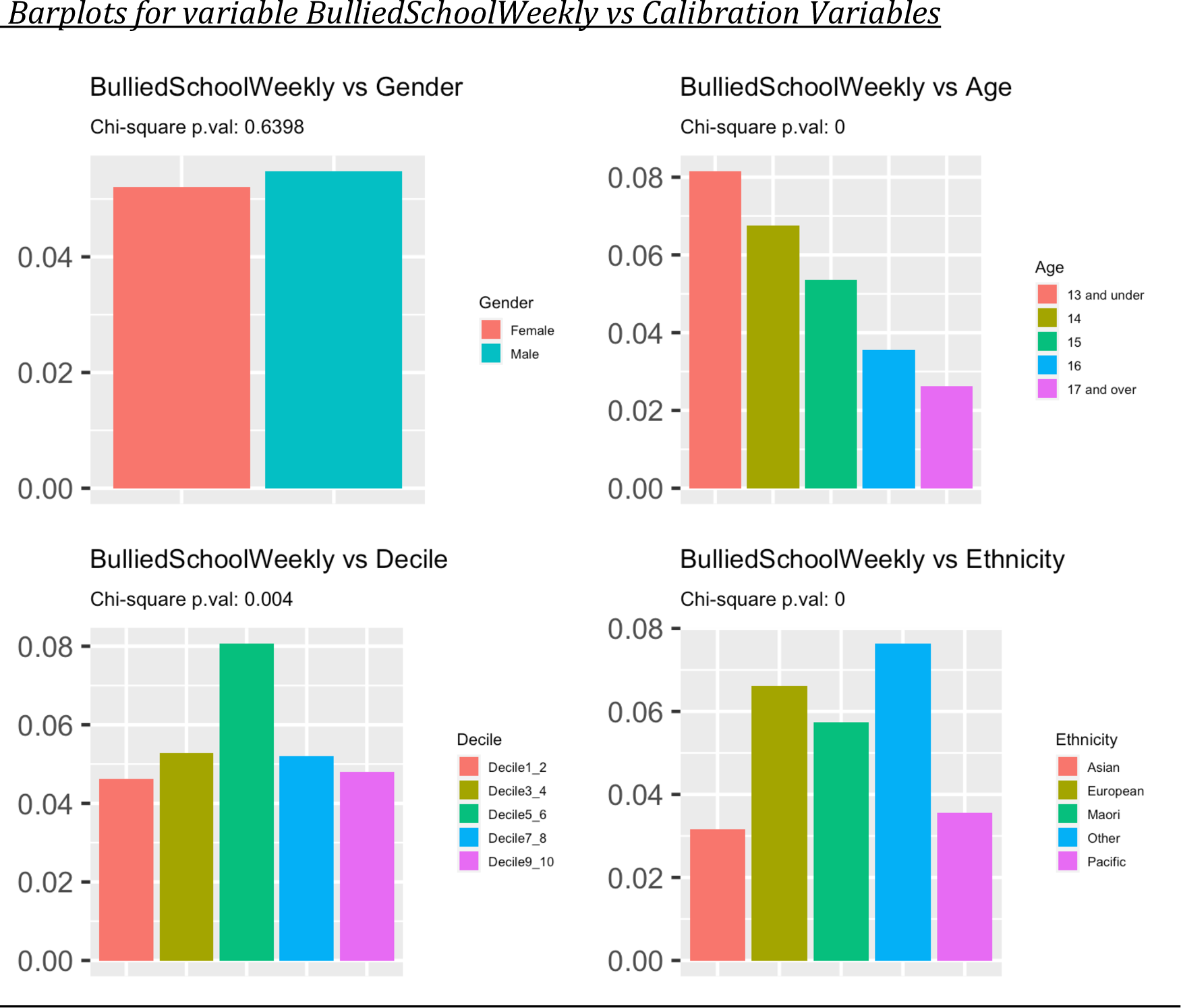

Supplement: S1 File — (ZIP) [file pone.0251177.s001.zip › Descriptive Stats File/Descriptive_Statistics8.tiff]

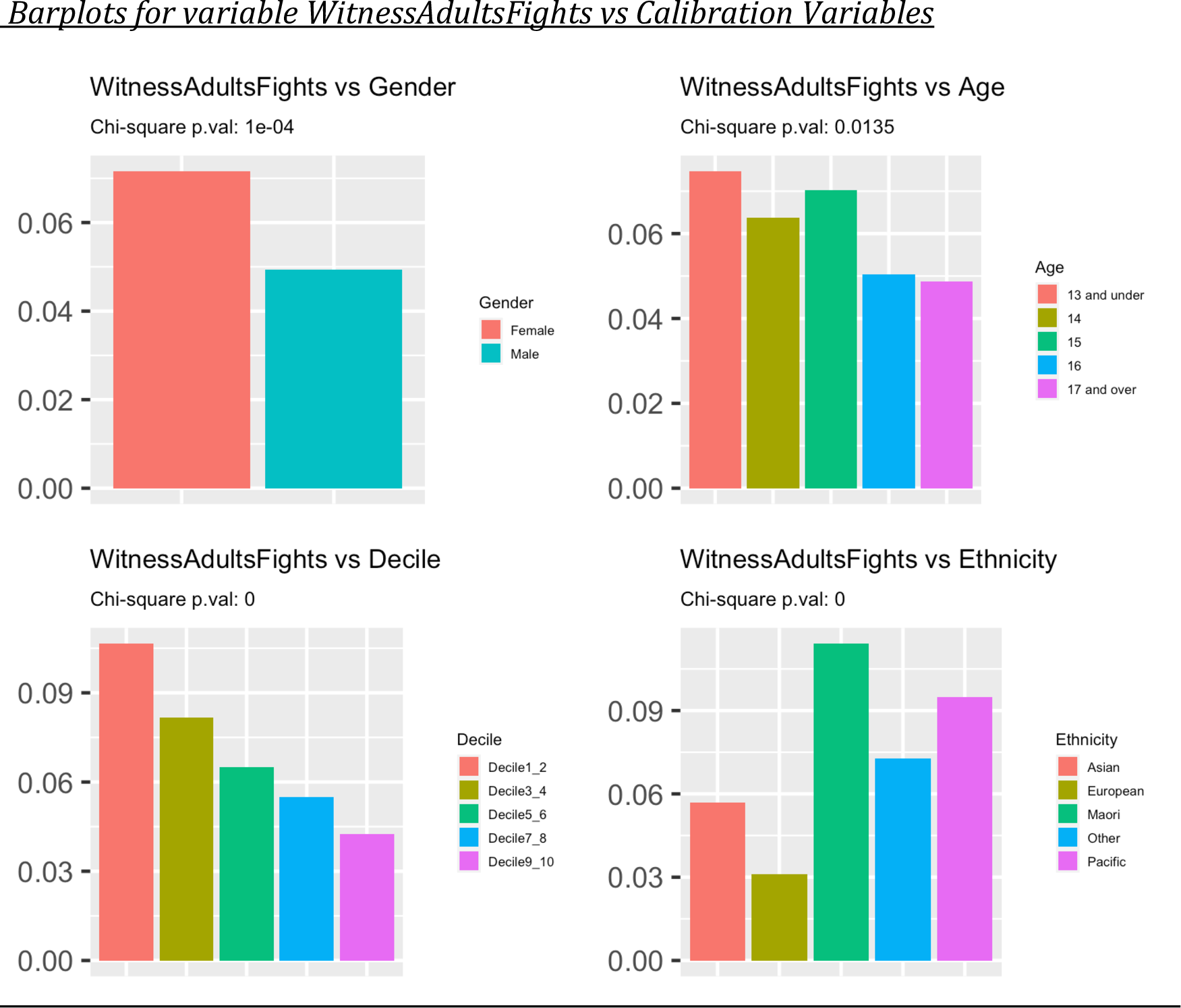

Supplement: S1 File — (ZIP) [file pone.0251177.s001.zip › Descriptive Stats File/Descriptive_Statistics9.tiff]
