# Supplementary material for: National estimates from the Youth ’19 Rangatahi smart survey: A survey calibration approach
Source: PLoS One. 2021 May 14;16(5):e0251177. doi: 10.1371/journal.pone.0251177 (PMC8121344; doi:10.1371/journal.pone.0251177)

Descriptive Statistics for 'National estimates from the Youth 19 Rangatahi smart survey: a survey calibration approach'

Barplots for variable MoveHouseOften vs Calibration Variables

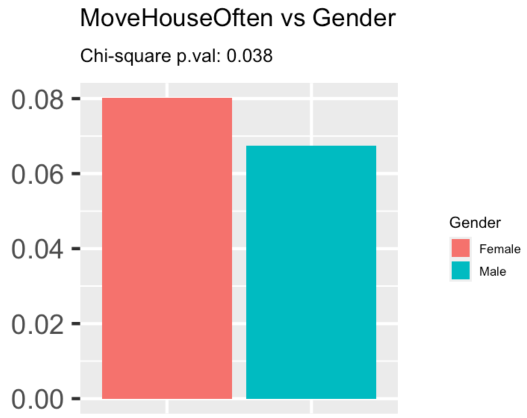

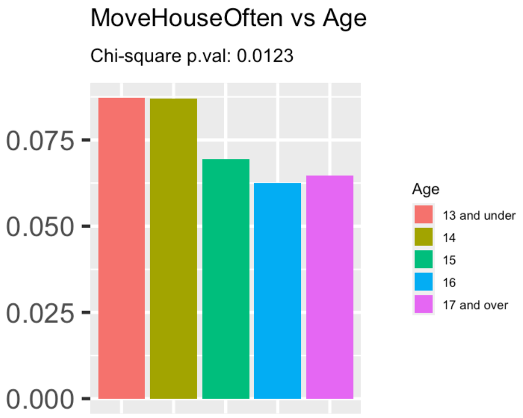

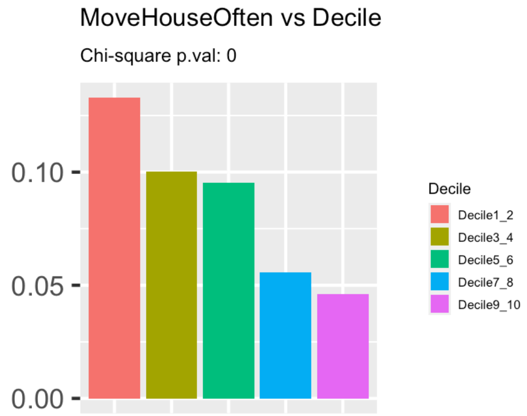

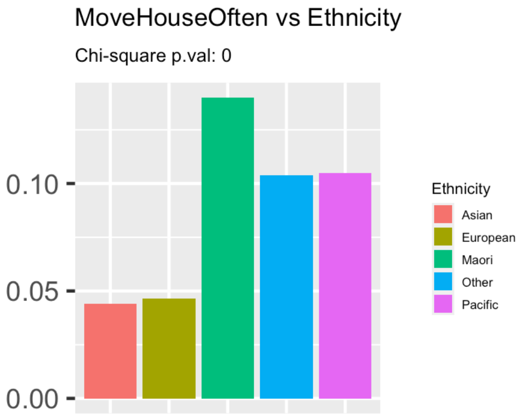

Barplots for variable ParentsWorryFood vs Calibration Variables

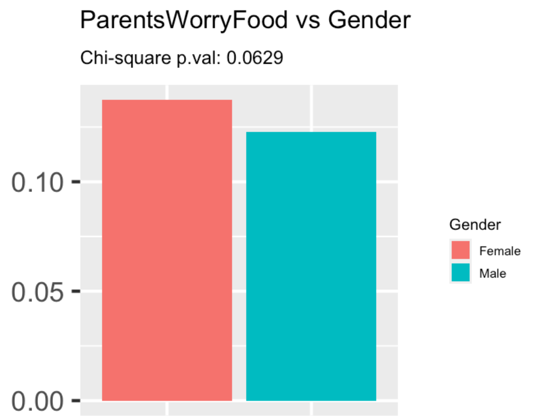

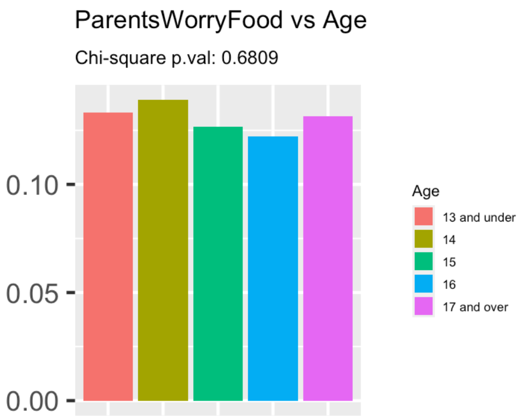

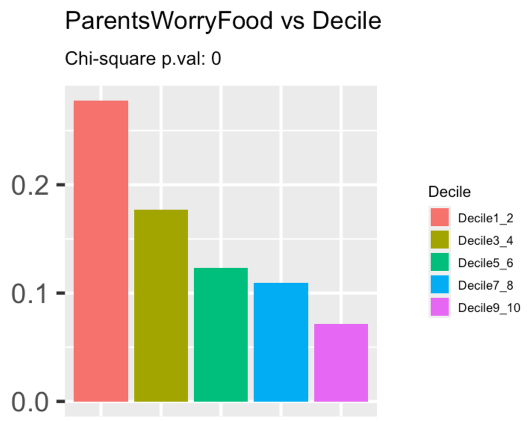

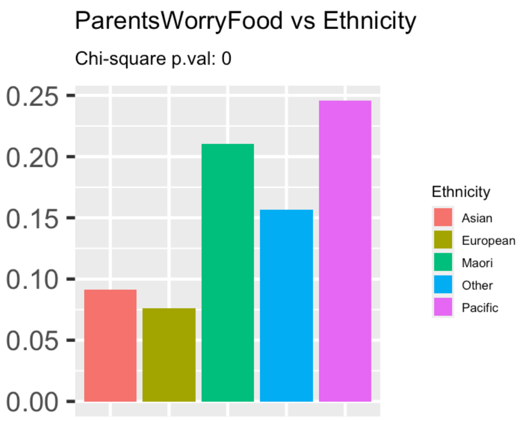

Barplots for variable EnoughTimeParent vs Calibration Variables

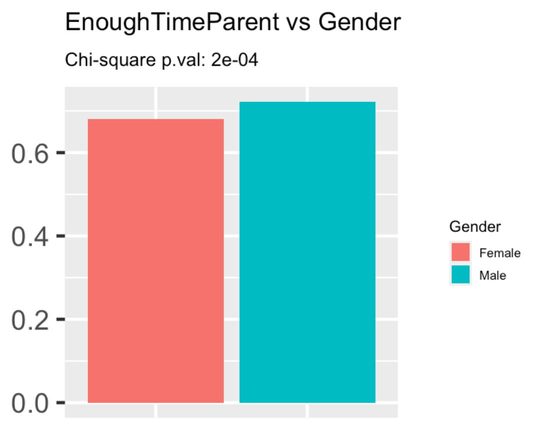

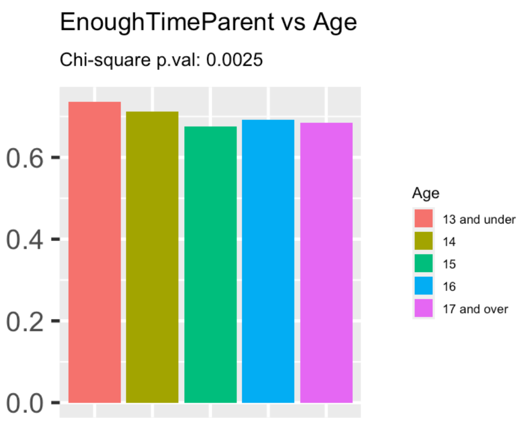

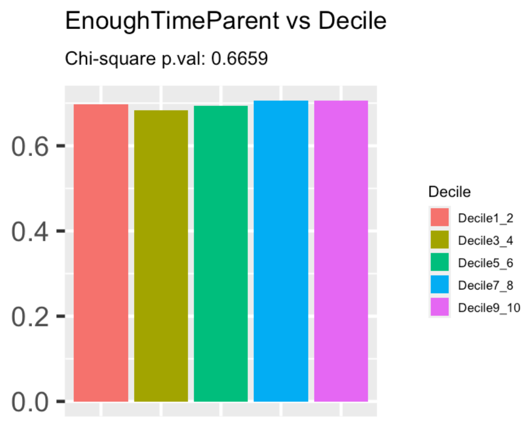

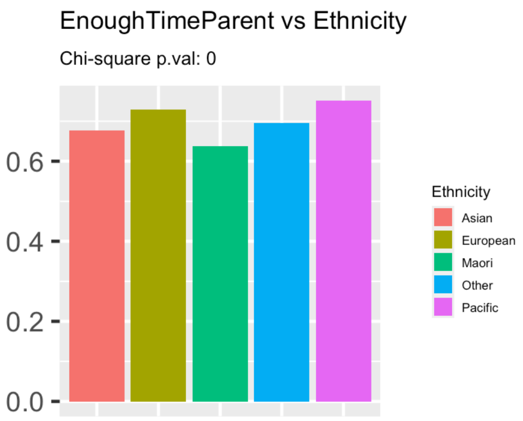

Barplots for variable FamilyKnowLocation vs Calibration Variables

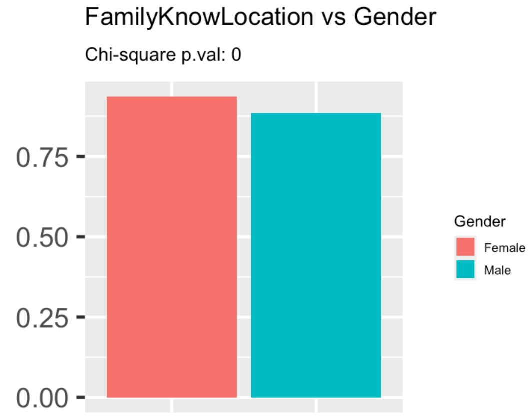

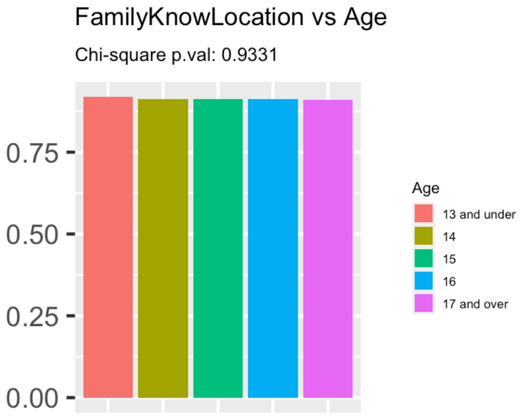

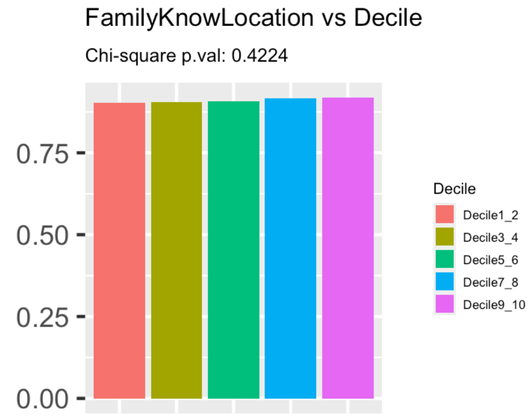

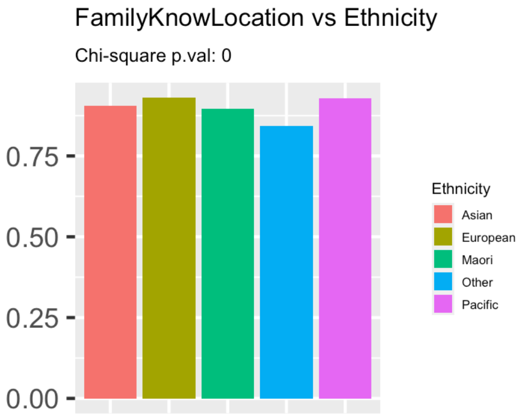

Barplots for variable SchoolCareALot vs Calibration Variables

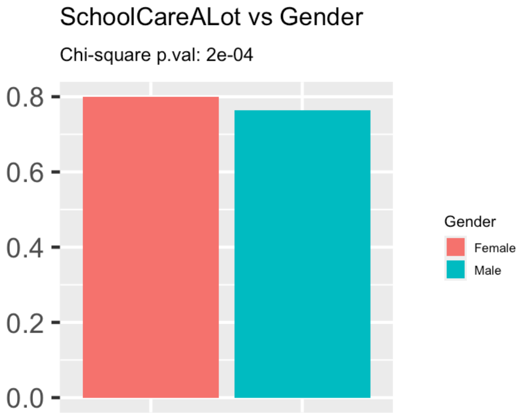

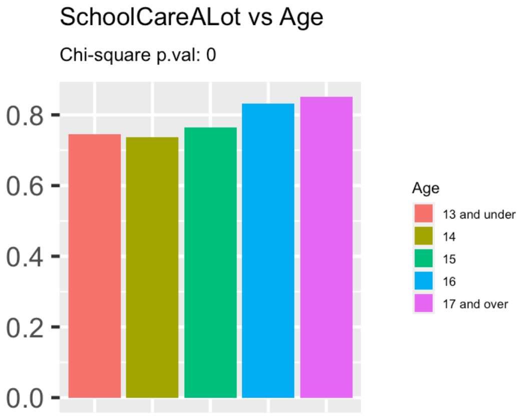

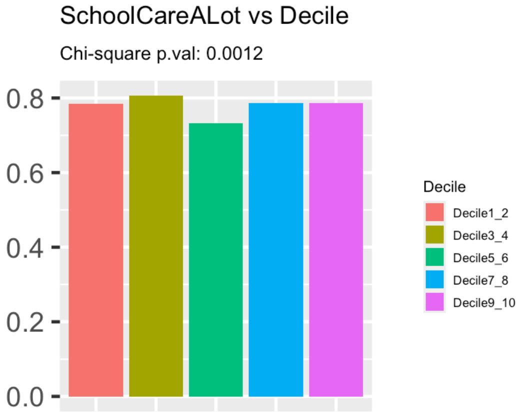

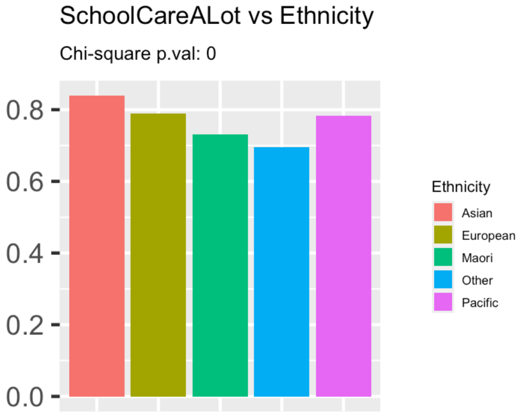

Barplots for variable AdultOKTalking vs Calibration Variables

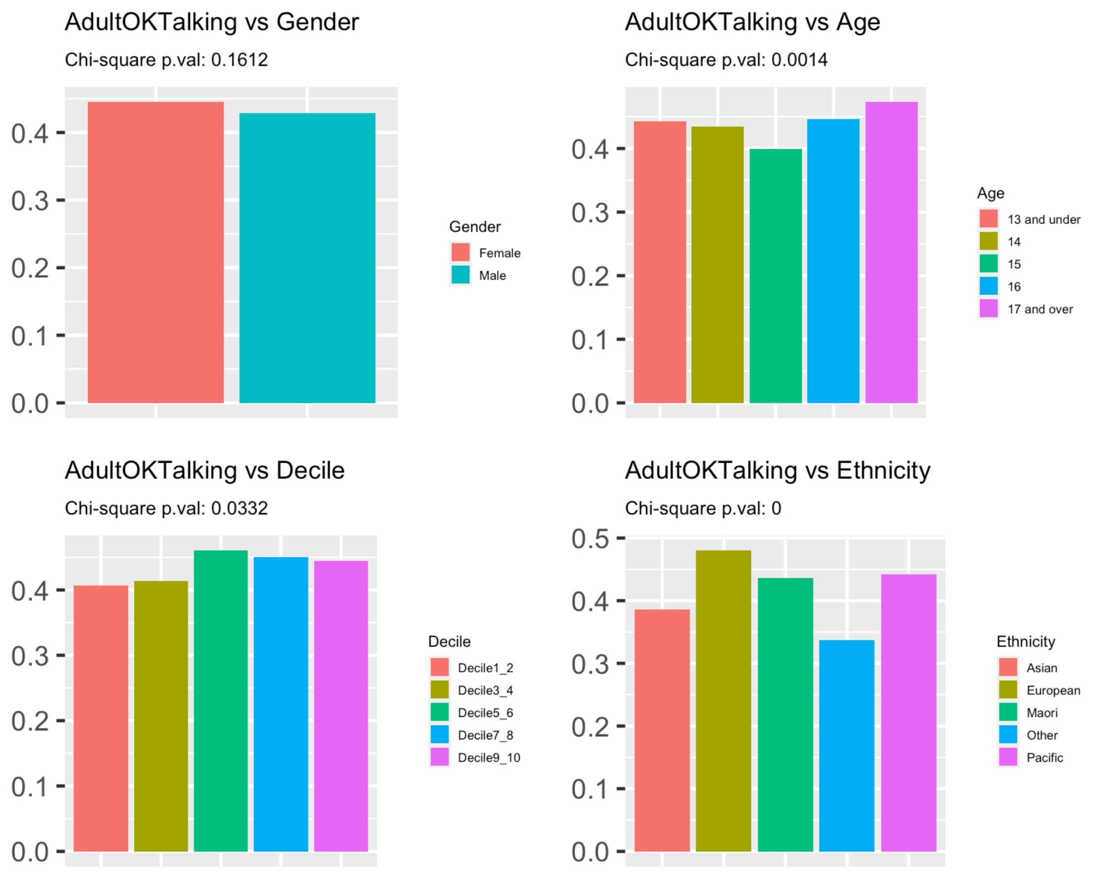

Barplots for variable FeelSafeNeigh vs Calibration Variables

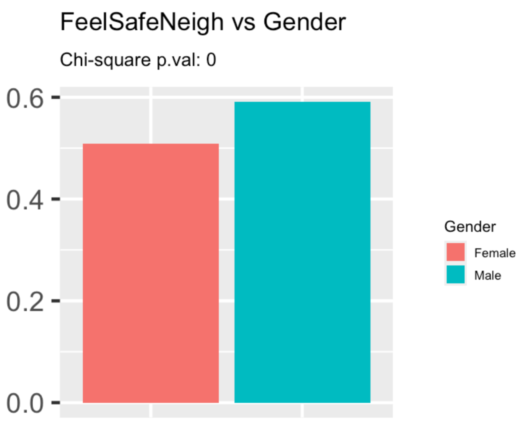

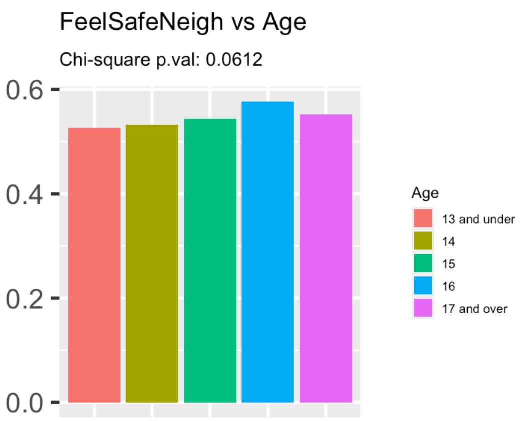

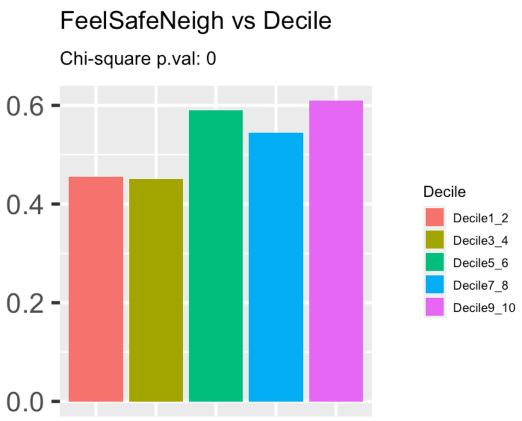

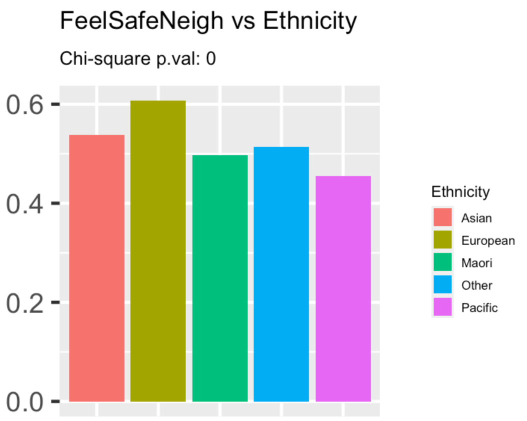

Barplots for variable BulliedSchoolWeekly vs Calibration Variables

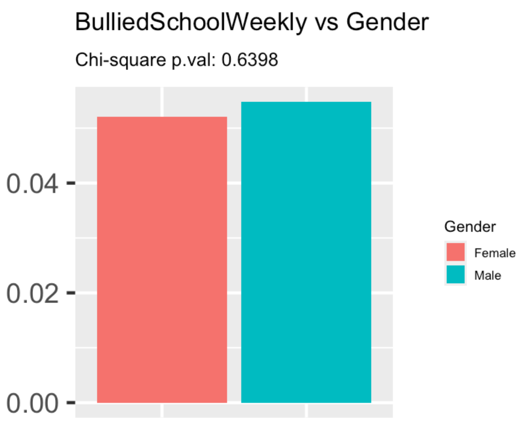

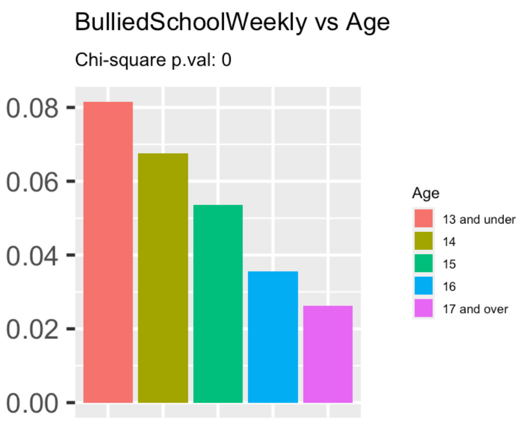

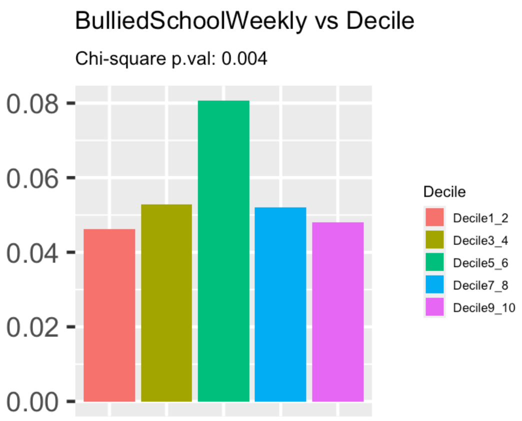

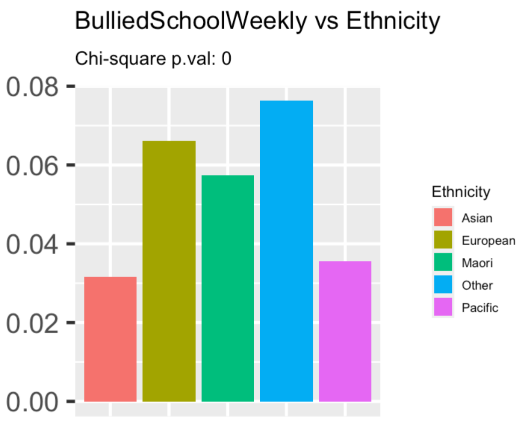

Barplots for variable WitnessAdultsFights vs Calibration Variables

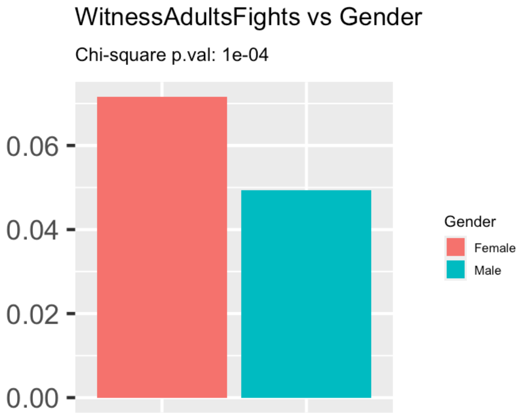

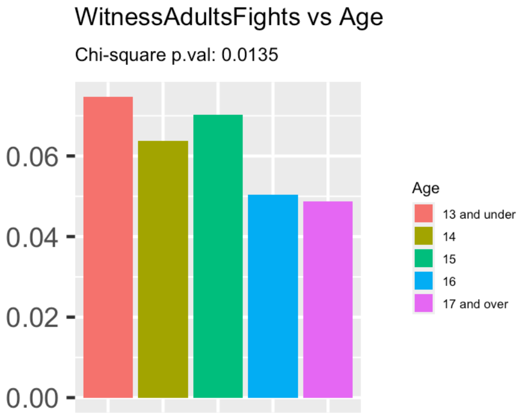

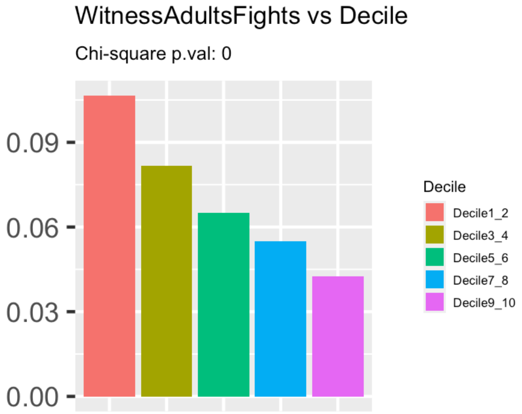

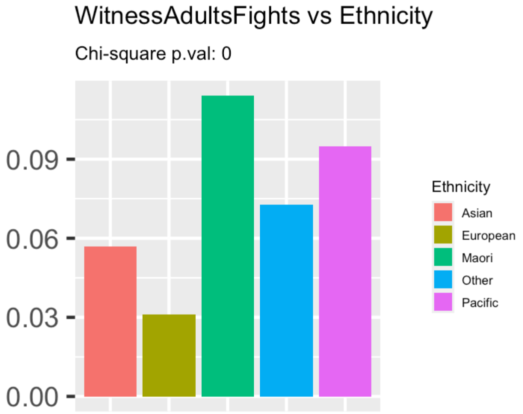

Barplots for variable SexuallyAbused vs Calibration Variables

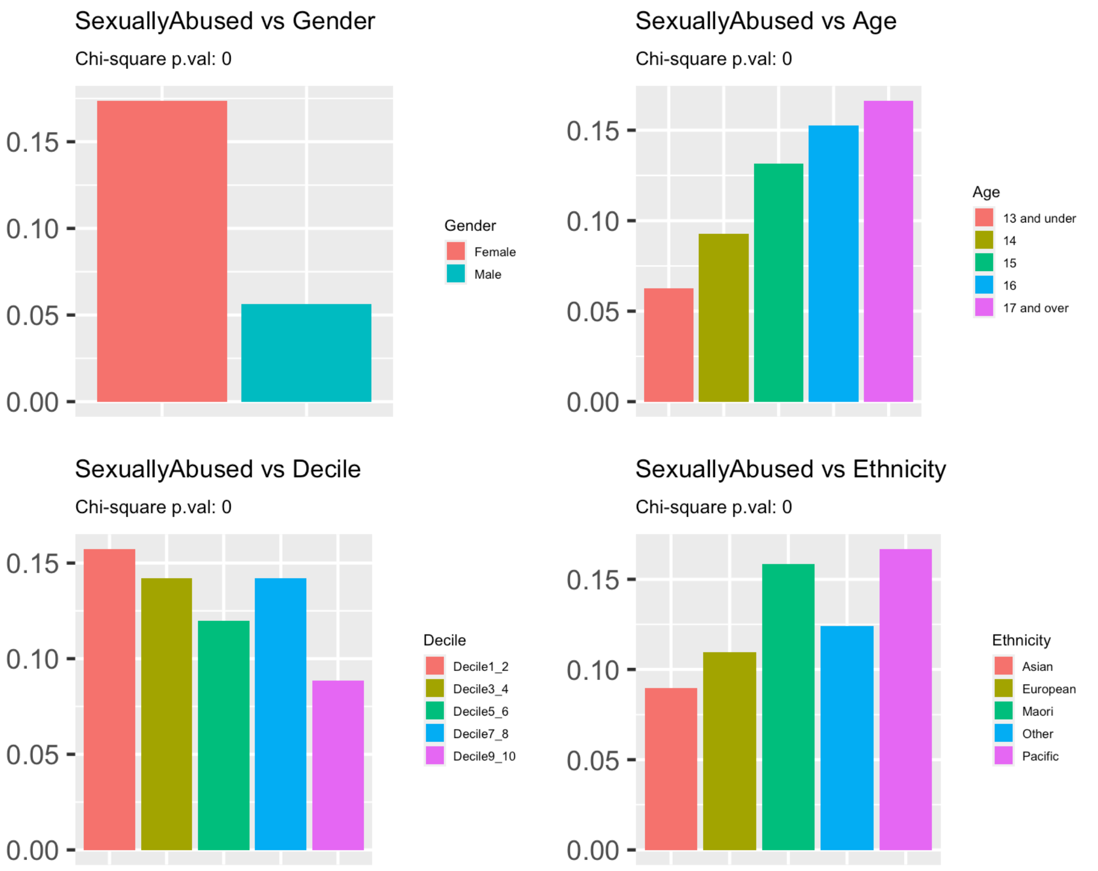

Barplots for variable HealthPoorRated vs Calibration Variables

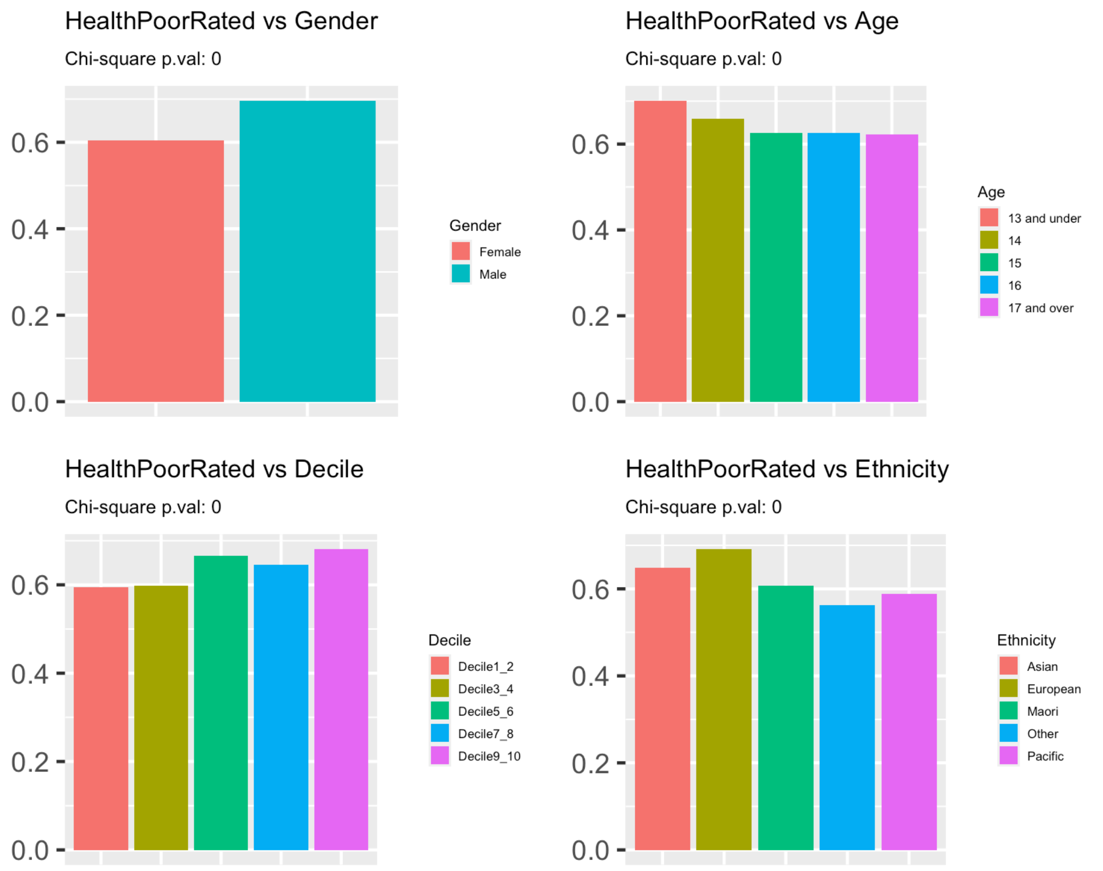

Barplots for variable DepressiveSymptoms28 vs Calibration Variables

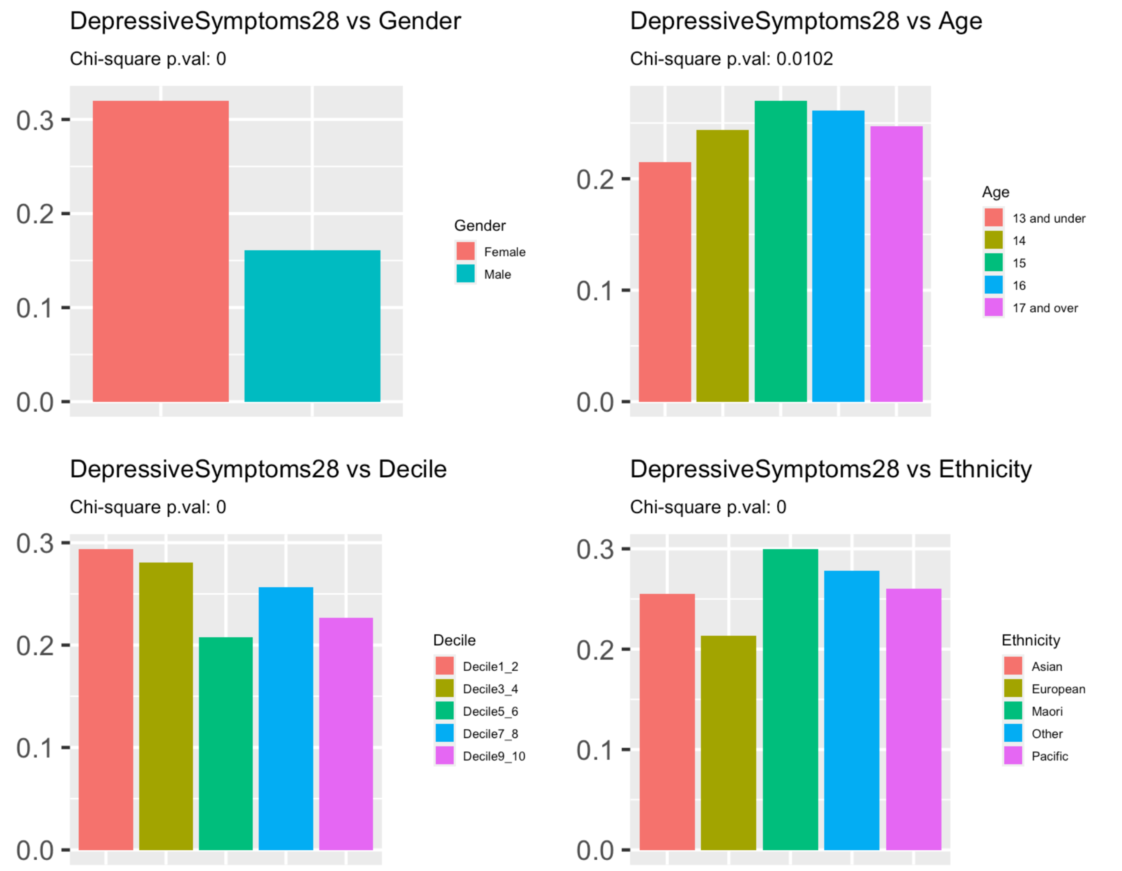

Barplots for variable AttemptSuicide vs Calibration Variables

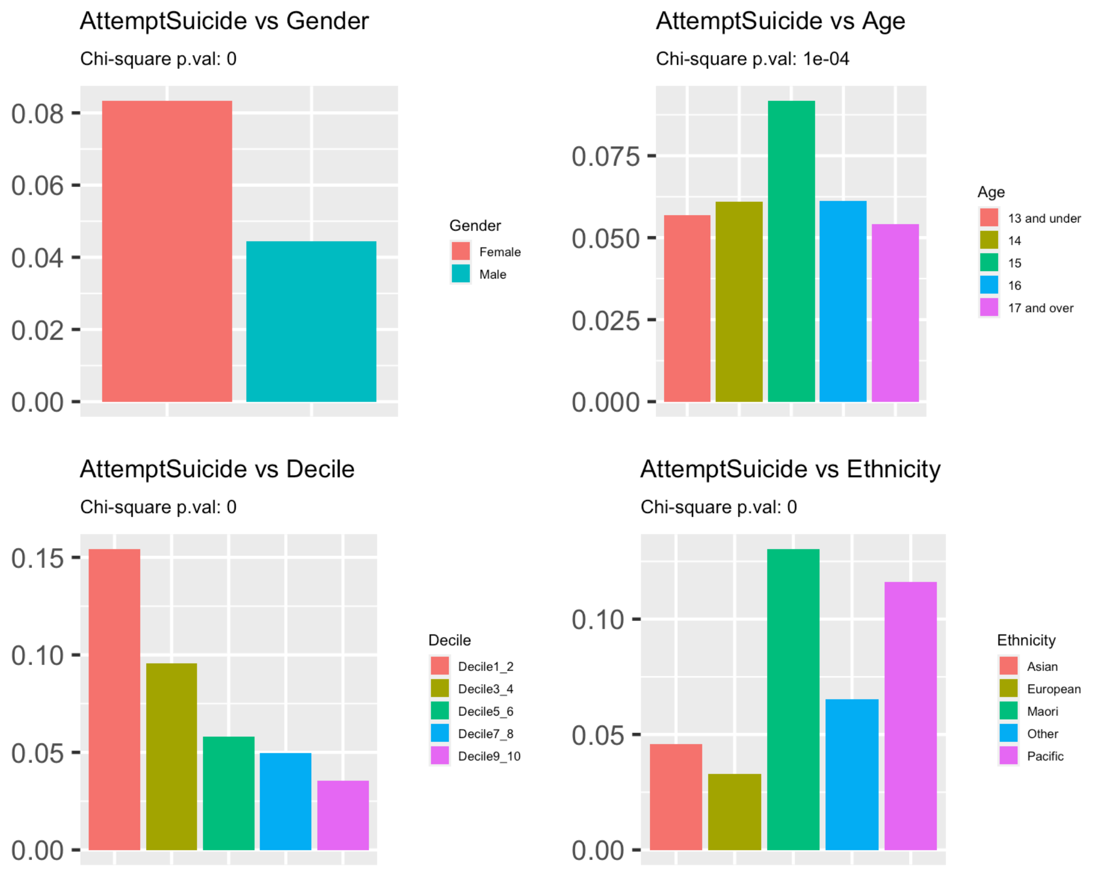

Barplots for variable MonthlySmoke vs Calibration Variables

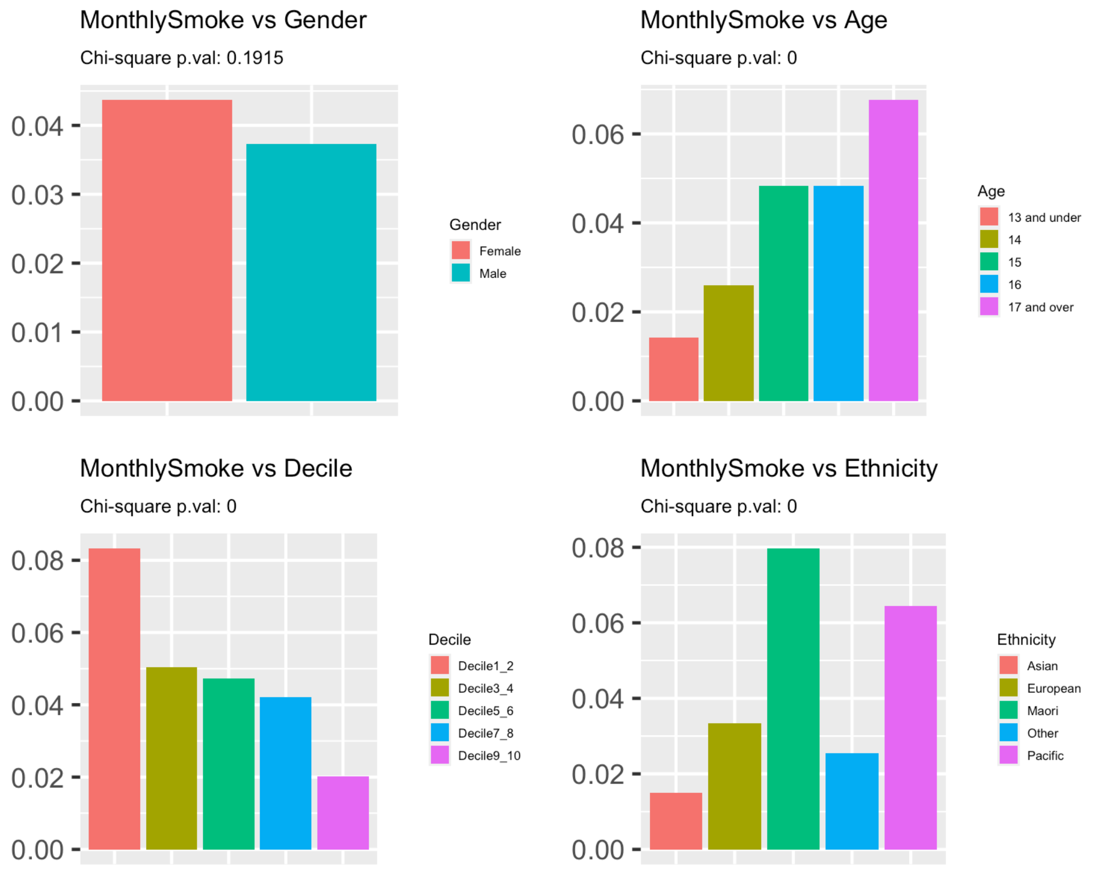

Barplots for variable BingeDrink vs Calibration Variables

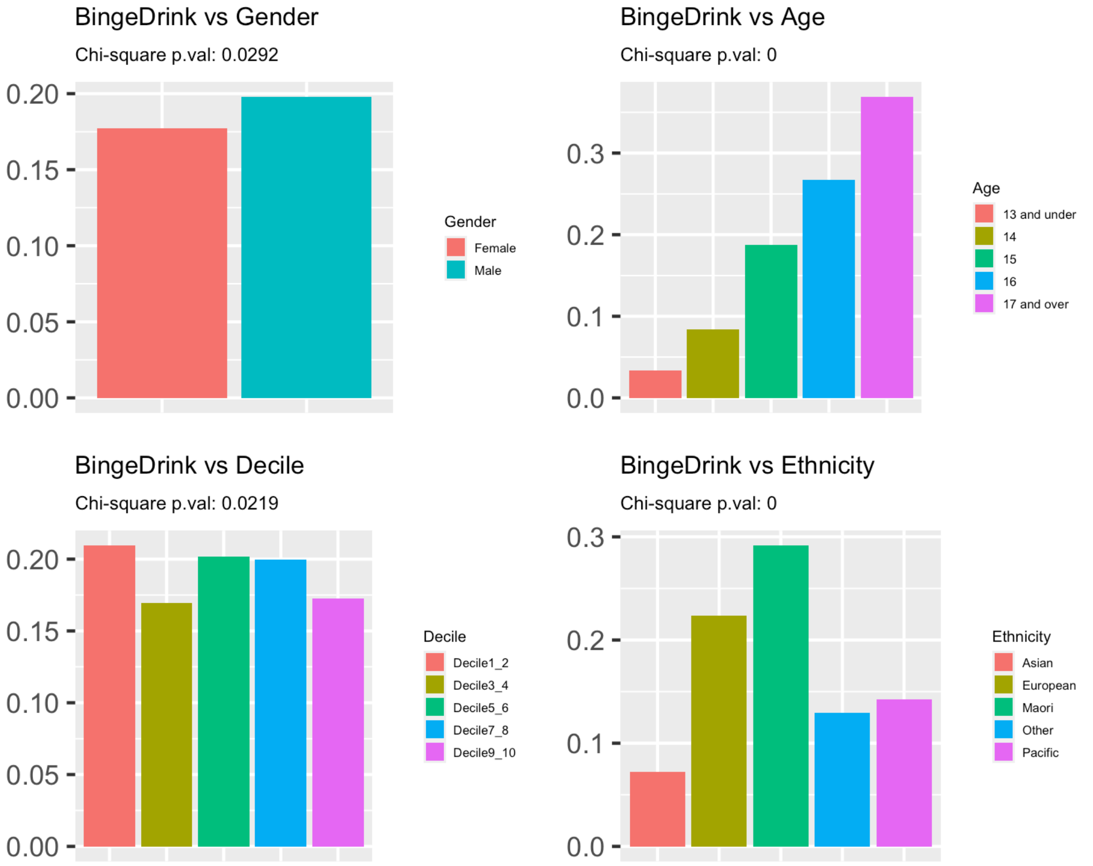

Barplots for variable MontlyMarijuana vs Calibration Variables

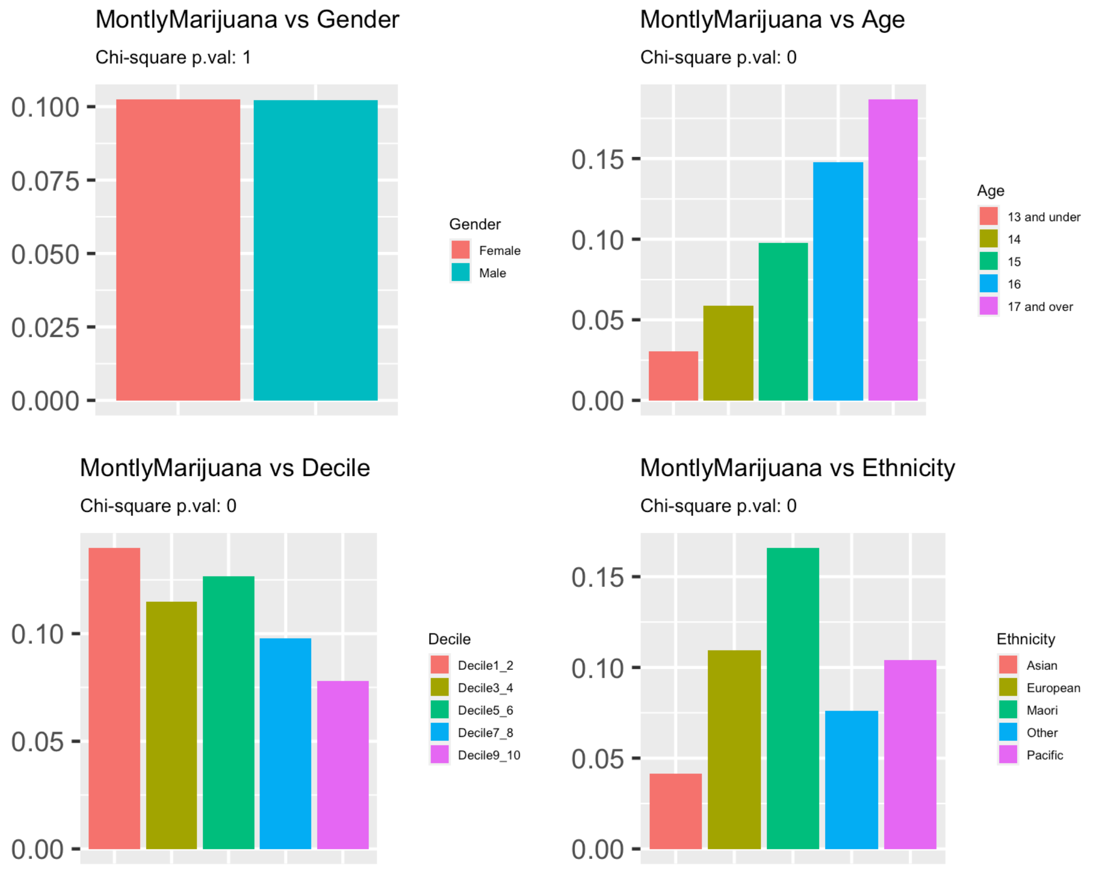

Barplots for variable EverHadSex vs Calibration Variables

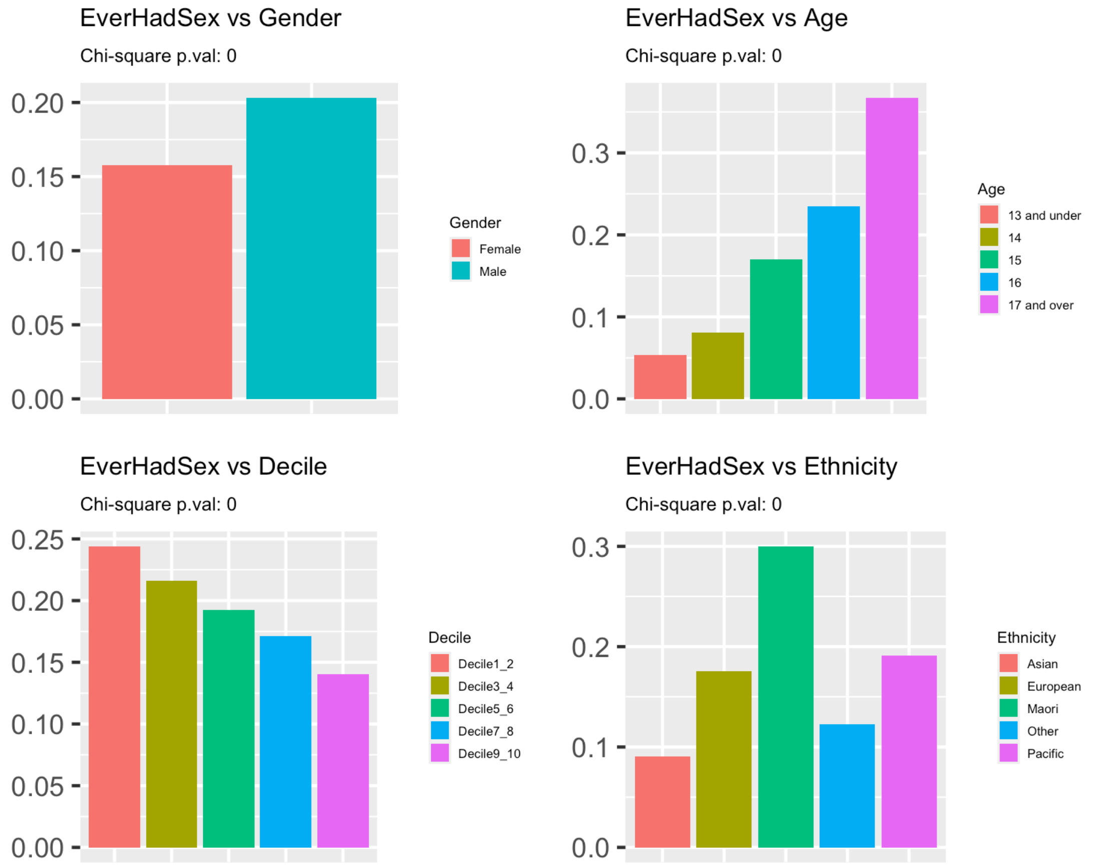

Barplots for variable CondomLastSex vs Calibration Variables

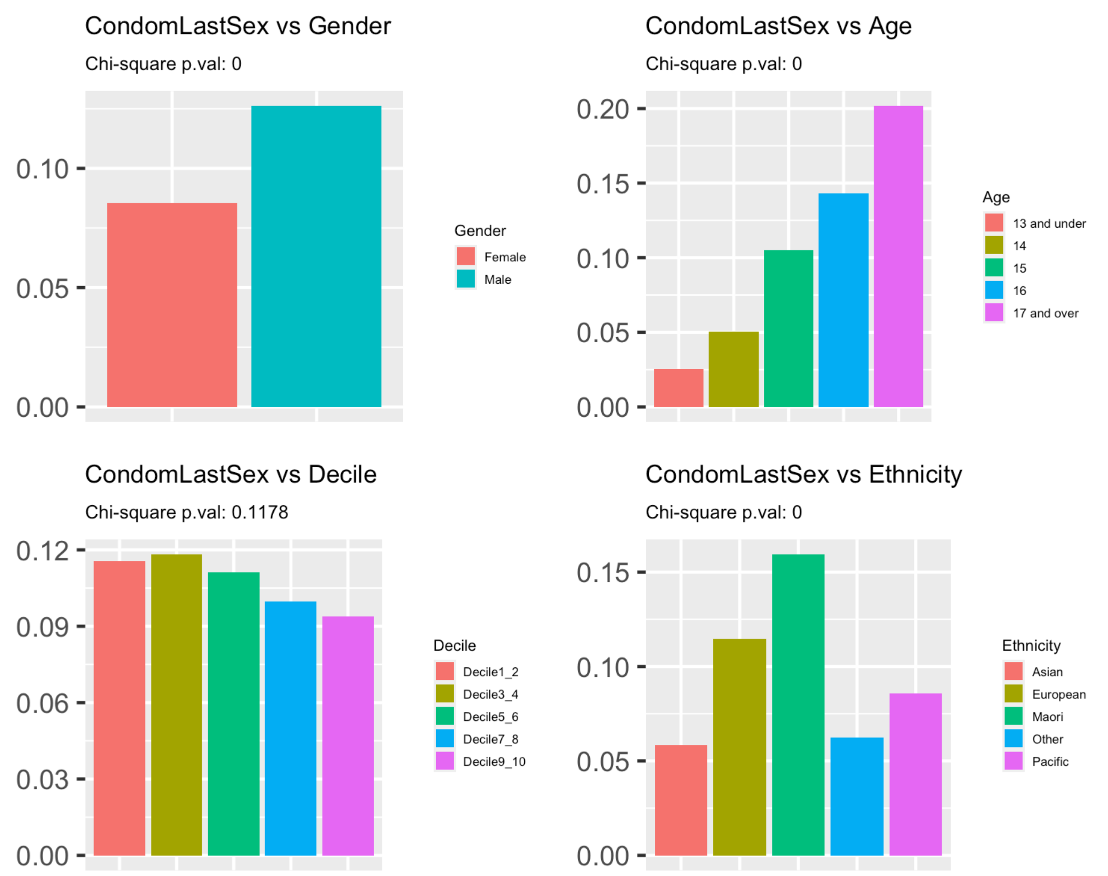

Barplots for variable EverPregnant vs Calibration Variables

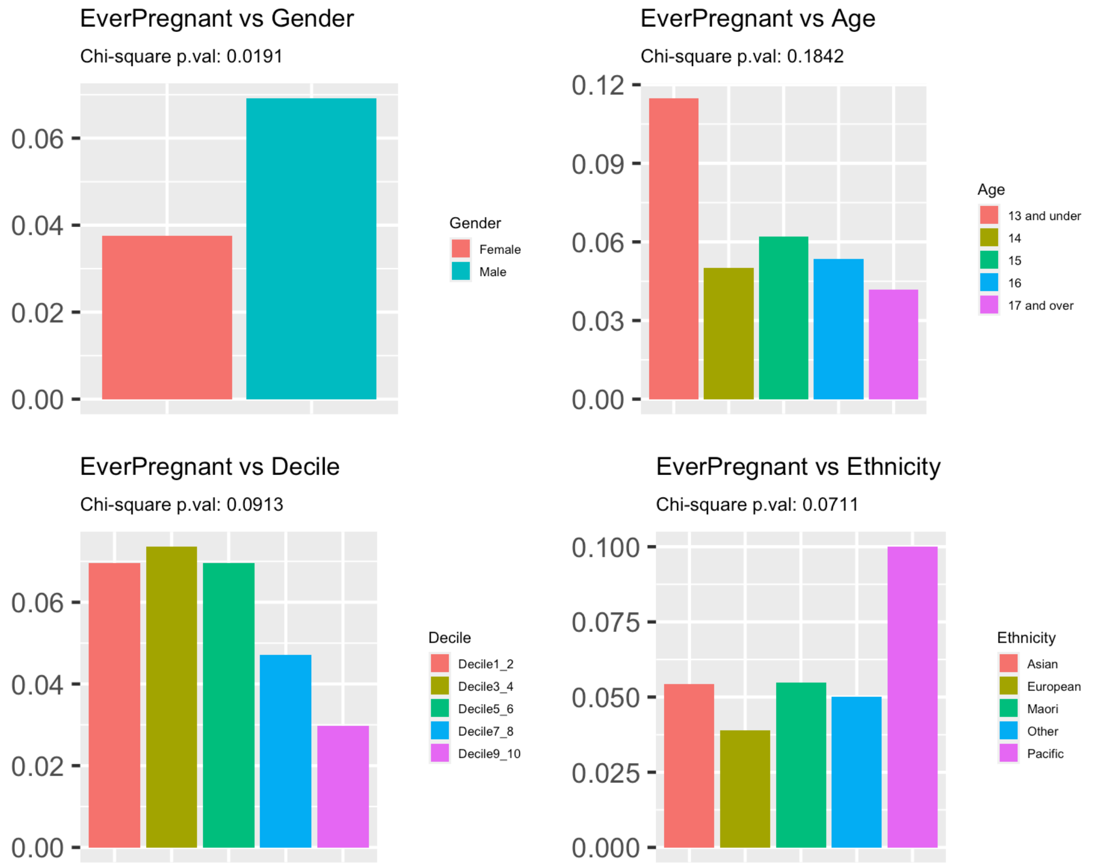

Barplots for variable ExerciseWeekly vs Calibration Variables

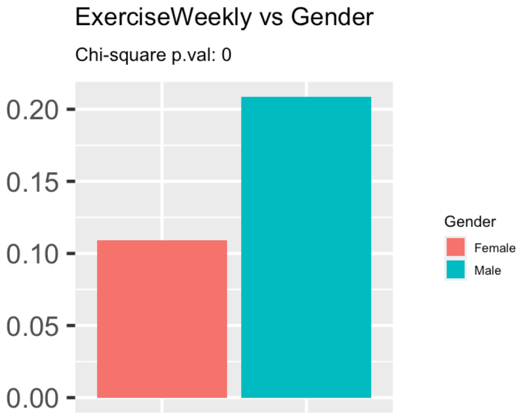

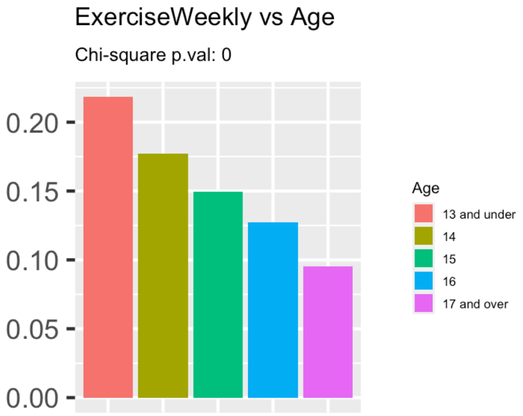

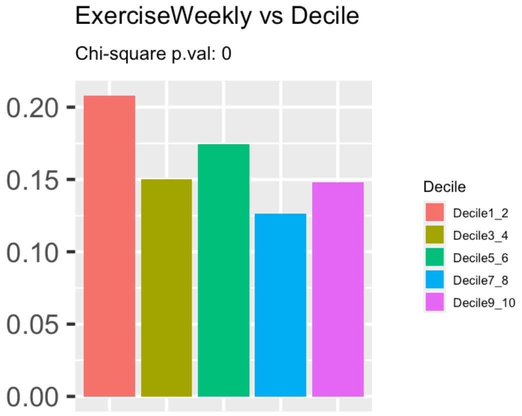

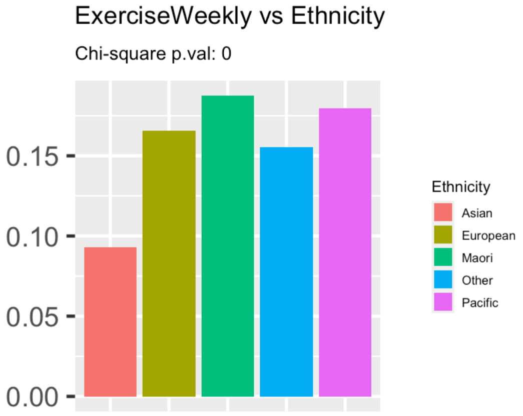

Barplots for variable WearSeatBelt vs Calibration Variables

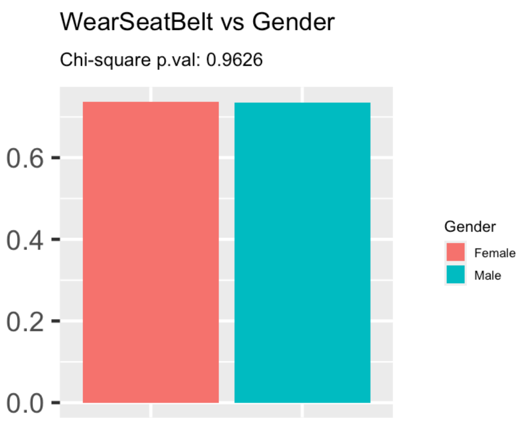

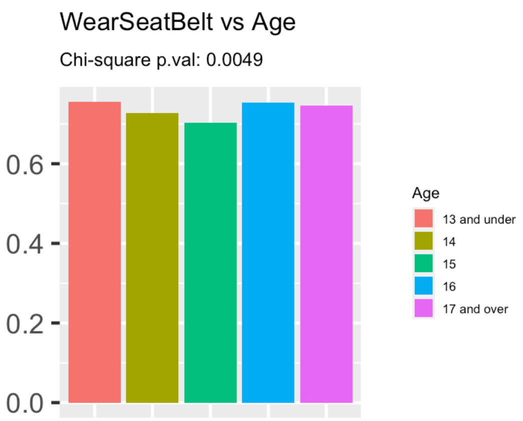

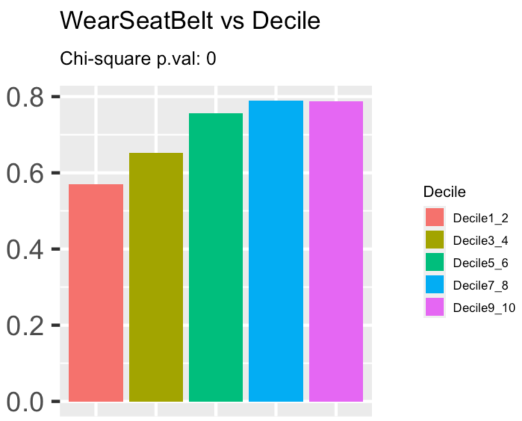

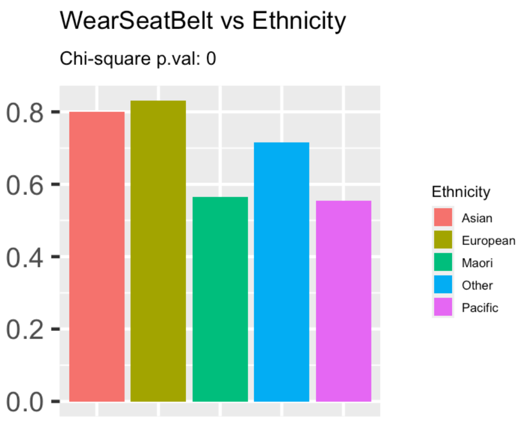

Barplots for variable PassengerRiskyDriver vs Calibration Variables

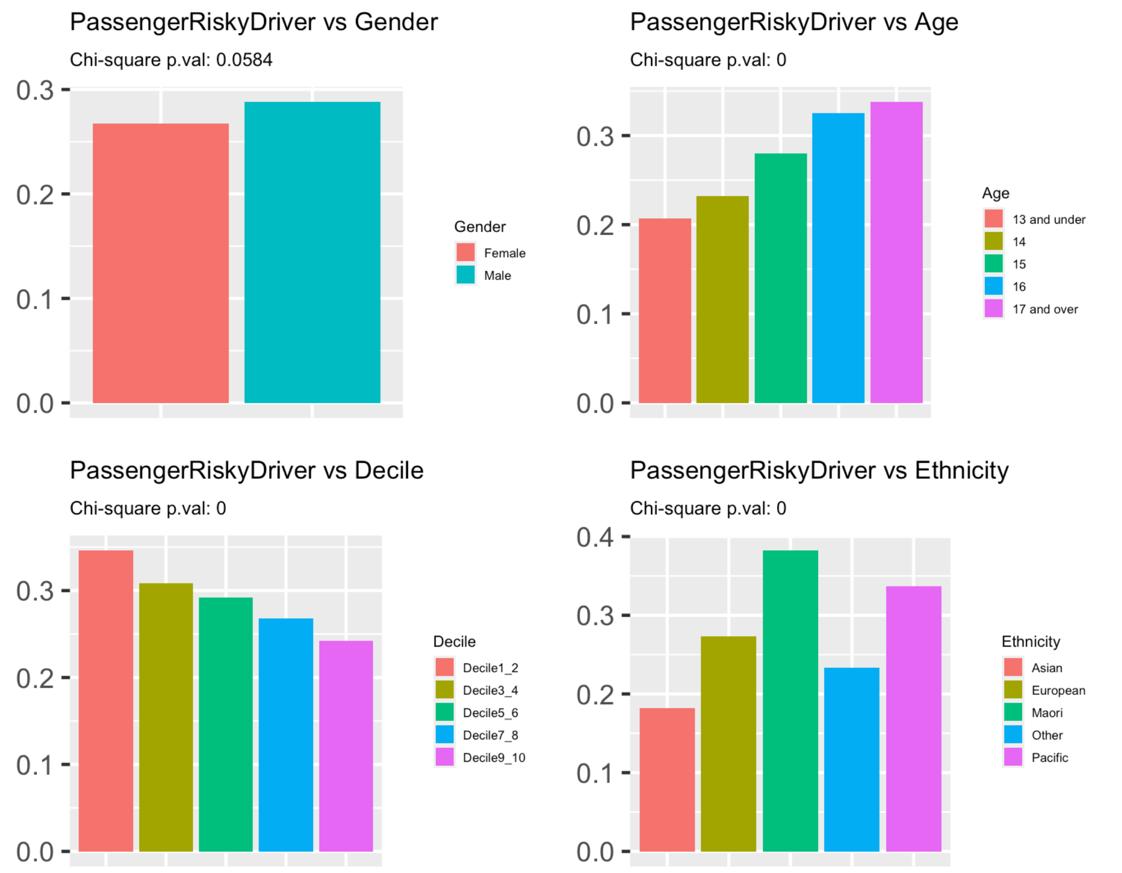

Barplots for variable RiskyDriverLastMonth vs Calibration Variables

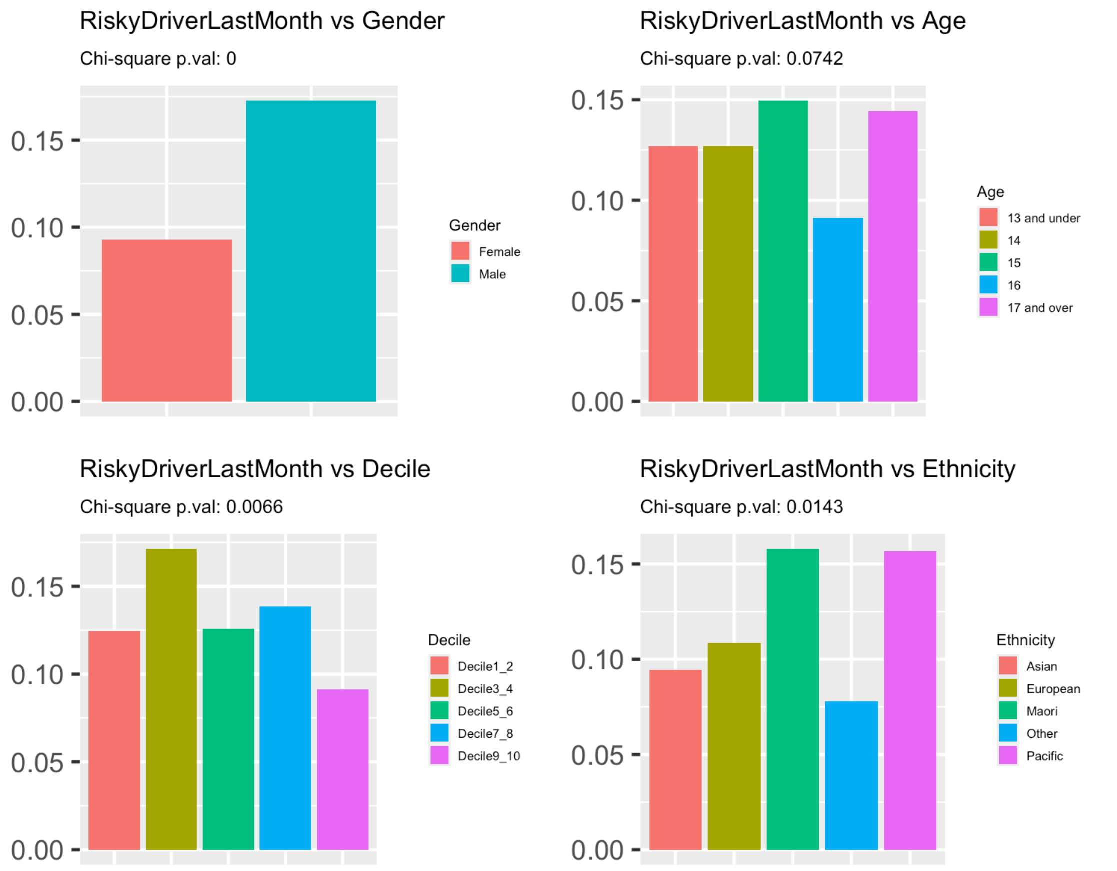

Barplots for variable HealthAccessLastMonth vs Calibration Variables

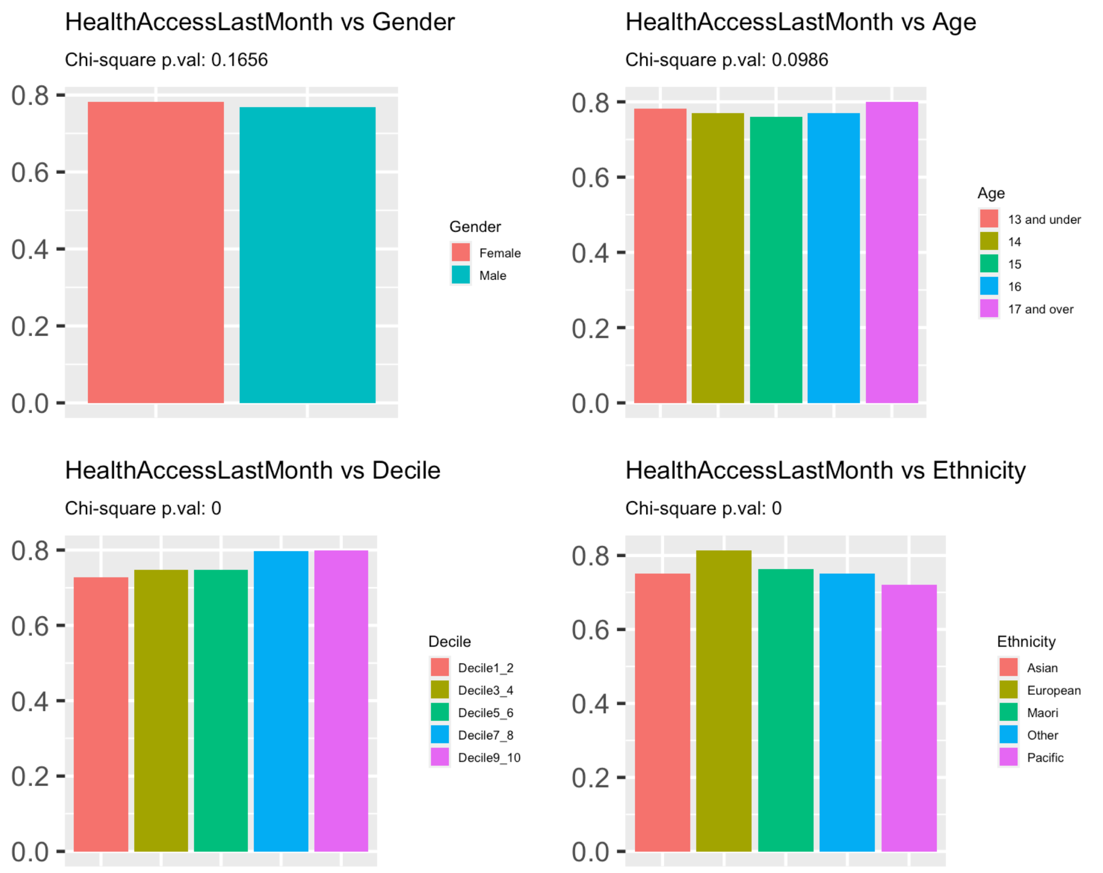

Barplots for variable UnableToSeeHealthProv vs Calibration Variables

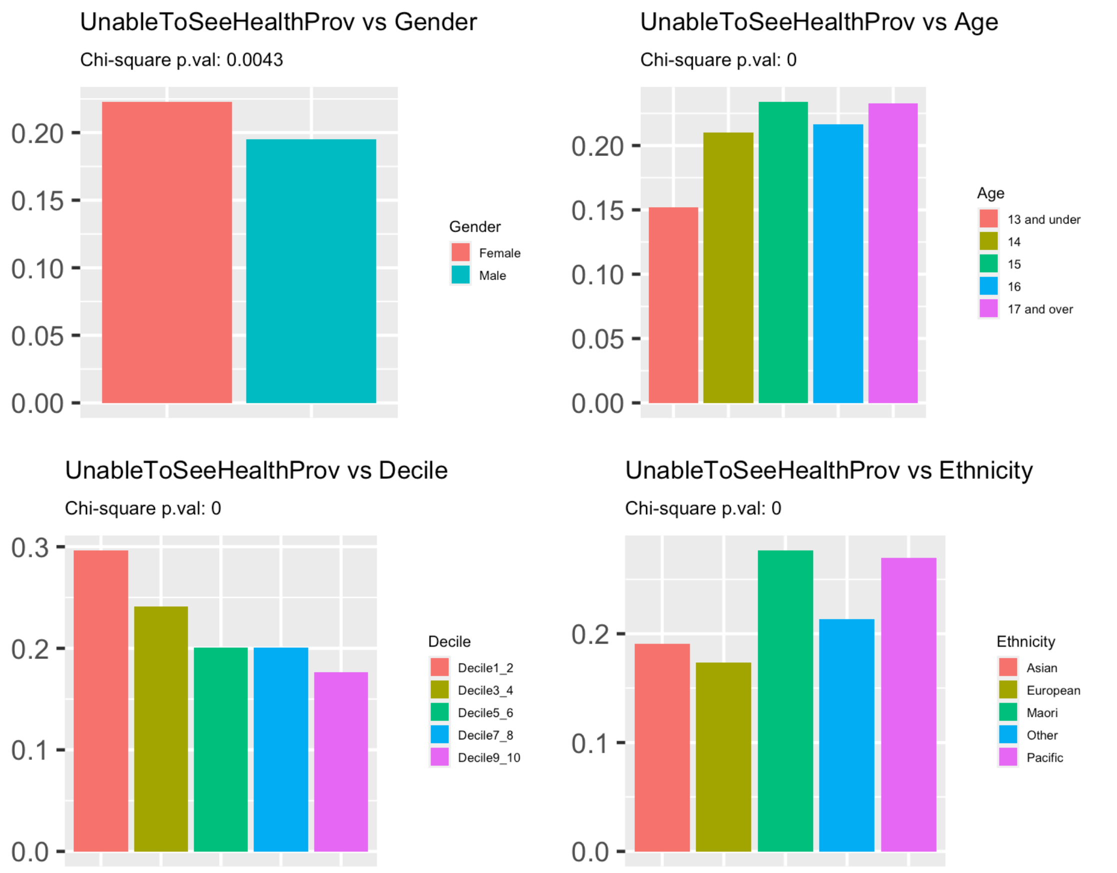

Supplement: S1 Statistics — (PDF) [file pone.0251177.s002.pdf]
