# Supplementary material for: Correction: National estimates from the Youth ’19 Rangatahi smart survey: A survey calibration approach
Source: PLoS One. 2021 Jun 17;16(6):e0253512. doi: 10.1371/journal.pone.0253512 (PMC8211149; doi:10.1371/journal.pone.0253512)
Supplement: S2 File — (PDF) [file pone.0253512.s002.pdf]

PLOS ONE

RESEARCH ARTICLE

National estimates from the Youth ’19 Rangatahi smart survey: A survey calibration approach

C. Rivera-Rodriguez[1]*, **T. C. Clark**[2], **T. Fleming**[3], **D. Archer**[3], **S. Crengle**[4], **R. Peiris-John**[5], **S. Lewycka**[6,7]

1 Department of Statistics, The University of Auckland, Auckland, New Zealand, **2** School of Nursing, University of Auckland, Auckland, New Zealand, **3** School of Health, Victoria University of Wellington, Wellington, New Zealand, **4** Department of Preventive and Social Medicine, University of Otago, Dunedin, New Zealand, **5** Department of Epidemiology and Biostatistics, The University of Auckland, Auckland, New Zealand, **6** Nuffield Department of Medicine, University of Oxford, Oxford, United Kingdom, **7** Oxford University Clinical Research Unit, Ho Chi Minh City, Vietnam

* c.rodriguez@auckland.ac.nz, clriverarodriguez@gmail.com

Check for updates

OPEN ACCESS

Citation: Rivera-Rodriguez C, Clark TC, Fleming T, Archer D, Crengle S, Peiris-John R, et al. (2021) National estimates from the Youth ’19 Rangatahi smart survey: A survey calibration approach. PLoS ONE 16(5): e0251177. https://doi.org/10.1371/journal.pone.0251177

Editor: Andrew R. Dalby, University of Westminster, UNITED KINGDOM

Received: September 23, 2020

Accepted: April 21, 2021

Published: May 14, 2021

Copyright: © 2021 Rivera-Rodriguez et al. This is an open access article distributed under the terms of the Creative Commons Attribution License, which permits unrestricted use, distribution, and reproduction in any medium, provided the original author and source are credited.

Data Availability Statement: The data underlying this study are owned by Youth19 (https://www.youth19.ac.nz/) and are available upon request. Interested and qualified researchers may submit data access requests to https://www.youth19.ac.nz/contact. The authors are members of the Youth2019 research group. However, other researchers would be able to access the same data on request.

Funding: TC - HRC (Health Research Council New Zealand) -grant number 17/315 https://www.hrc.

Abstract

Background

Significant progress has been made addressing adolescent health needs in New Zealand, but monitoring and gathering high quality estimates of adolescent health and social issues remains challenging and resource intensive. Previous nationally representative secondary school surveys were conducted in New Zealand in 2001, 2007 and 2012, as part of the Youth2000 survey series. This paper focuses on a fourth survey conducted in 2019 (https://www.youth19.ac.nz/). The 2019 survey had a regional sampling strategy rather than a national sampling strategy as in previous years. The survey also included kura kaupapa Māori schools (Māori language immersion schools), as well as mainstream secondary schools. This paper presents the overall study methodology, and a weighting and calibration framework in order to provide estimates that reflect the national student population, and enable comparisons with the previous surveys to monitor trends.

Methods

Youth19 was a cross sectional, self-administered health and wellbeing survey of New Zealand high school students. The survey population was secondary school students of New Zealand aged 12 to 18 years (school years 9–13). The study population was drawn from three education regions: Auckland, Tai Tokerau (Northland) and Waikato. These are the most ethnically diverse regions in New Zealand and account for 46% of the adolescent population in New Zealand. The sampling design was two-stage clustered stratified, where schools were the clusters, and strata were defined by kura schools and educational regions. There were four strata, formed as follows: kura schools (Tai Tokerau, Auckland and Waikato regions combined), mainstream-Auckland, mainstream-Tai Tokerau and mainstream-Waikato. From each stratum, 50% of the schools were randomly sampled and then 30% of students from the selected schools were invited to participate. All students in the kura kaupapa

govt.nz/ The funders had no role in study design, data collection and analysis, decision to publish, or preparation of the manuscript. TF - HRC (Health Research Council New Zealand) -grant number 18/473 https://www.hrc.govt.nz/ The funders had no role in study design, data collection and analysis, decision to publish, or preparation of the manuscript.

Competing interests: The authors have declared that no competing interests exist.

schools were invited to participate. In order to make more precise estimates and adjust for differential non-response, as well as to make nationally relevant estimates and allow comparisons with the previous national surveys, we calibrated the sampling weights to reflect the national secondary school student population.

Results

There were 45 mainstream and 4 kura schools included in the final sample, and 7,374 mainstream and 347 kura students participated in the survey. There were differences between the sampled population and the national secondary school student population, particularly in terms of sex and ethnicity, with a higher proportion of females and Asian students in the study sample than in the national student population. We calculated estimates of the totals and proportions for key variables that describe risk and protective factors or health and wellbeing factors. Rates of risk-taking behaviours were lower in the sampled population than what would be expected nationally, based on the demographic profile of the national student population. For the regional estimates, calibrated weights yield standard errors lower than those obtained with the unadjusted sampling weights. This leads to significantly narrower confidence intervals for all the variables in the analysis. The calibrated estimates of national quantities provide similar results. Additionally, the national estimates for 2019 serve as a tool to compare to previous surveys, where the sampling population was national.

Conclusions

One of the main goals of this paper is to improve the estimates at the regional level using calibrated weights to adjust for oversampling of some groups, or non-response bias. Additionally, we also recommend the use of calibrated estimators as they provide nationally adjusted estimates, which allow inferences about the whole adolescent population of New Zealand. They also yield confidence intervals that are significantly narrower than those obtained using the original sampling weights.

1. Background

High quality population-based data that provides estimates of adolescent behaviours are essential for the planning of services, programmes, policy and for monitoring equitable outcomes. However undertaking such surveys are expensive, complex and resource-intensive. Significant progress has been made addressing adolescent health needs in New Zealand and globally since the turn of the century with reductions in morbidity and mortality [1, 2], and increased data surveillance monitoring of adolescent health trends. However, some areas, such as mental health issues remain a concern [3], alongside new important areas have emerged that impact adolescent wellbeing, such as vaping and social media use [4]. Monitoring and tracking trends in adolescent health are vital, particularly for Indigenous, ethnic and sexual minority youth, those with disabilities and from poor neighbourhoods [5].

To investigate the health and wellbeing of young New Zealanders, as part of the Youth2000 survey series, nationally representative secondary school surveys were conducted in New Zealand in 2001, 2007 and 2012, and 2019 [1,2]. These surveys provided an opportunity to assess the situation at each time point, and monitor trends in key indicators of health and wellbeing.

These surveys randomly sampled secondary schools across New Zealand, and from each school that consented to take part, a random sample of around 8,500 year 9–13 students were selected to participate. More recent estimates are required in order to monitor progress and identify areas that need further attention. In 2019 (https://www.youth19.ac.nz/), the schools sampled only included three regions (Waikato, Auckland and Tai Tokerau/Northland), rather than from the whole country, due to loss of Government contract. Alternative funding was sought, and due to logistical and budgetary constraints a pragmatic decision to survey a smaller proportion of students and regions was made.

In this paper we present the overall study methodology, and how we have utilized a weighting and calibration framework that can provide estimates that reflect the national student population, ensure that ethnic groups, particularly Māori are adequately represented and enable comparisons with the estimates from previous surveys.

2. Methods

2.1. Study design

Youth19 was a cross-sectional, self-administered health and wellbeing survey of New Zealand secondary school students. Full details of the methods have been published elsewhere [6]. The study had the following aims:

1. To collect, analyse and disseminate accurate, comprehensive and timely information on the health and wellbeing of young people living in Tai Tokerau, Auckland and Waikato Regions, in order to inform and improve policies and practices;
2. To evaluate how whanaungatanga influences health outcomes for rangatahi Māori;
3. To test the potential benefits of incorporating opt-in access to links for support services within a survey.

2.2. Target and study populations

New Zealand secondary school students (aged 13–18 years, school years 9–13) were surveyed across three regions: Auckland, Tai Tokerau/Northland and Waikato. Almost half the New Zealand youth population resides in these areas (46%), these are the most ethnically diverse regions in New Zealand and include a range of urban and rural settings as well as a breadth of socio-economic groupings. These three regions were chosen to represent the diversity of the New Zealand population, and to ensure that the number of participants from each of the main ethnic groups provided sufficient statistical power for sub-group analyses. Previous population-based studies have used these three regions and found them to be representative of national statistics [7].

2.3. Sampling design

We used the Education Counts 2017 national list of schools as our sampling frame [8], and excluded schools from regions other than Auckland, Tai Tokerau and Waikato. We used a two-stage cluster sampling design. We included single sex, co-education, public, private and fully integrated schools that had over 50 students in years 9–13. As in the previous three surveys, schools with under 50 students were excluded for logistical reasons, hence the conclusions presented here are only for students attending schools with over 50 students. Special schools that only included students who had intellectual or physical disabilities which would have prevented them from being able to participate in the survey where excluded. We stratified

our sample by kura schools and educational regions. There were four strata, formed as follows: kura schools (Tai Tokerau, Auckland and Waikato regions), mainstream-Auckland, mainstream-Tai Tokerau and mainstream-Waikato.

There were 161 eligible mainstream schools (100 in Auckland, 23 in Tai Tokerau and 38 in Waikato). From each stratum, 50% of the schools were randomly sampled using a random number generator. All selected schools were invited to participate through email and follow up phone calls. We piloted the survey in two additional schools from the same sampling frame in Auckland in 2019, these two schools were purposively selected. These were large ethnically and socio-economically diverse schools. Minimal changes were made to the survey after piloting, and these schools have been included in the total. We visited the other schools that agreed to participate between May and September 2019. We randomly sampled 30% of students on the school roll to be invited to participate in the study. One mainstream school also requested 100% of students be invited and this was done.

There were 8 eligible kura kaupapa Māori schools in the three study regions, and two from each region (6 in total) were invited to participate. These schools are smaller than mainstream schools and include immersion in Māori language and culture. Four schools participated and all students in these kura kaupapa schools were invited to participate.

We calculated sample weights as inverse probability weights using the sampling design described above. This design is described in detail in Table 1 and Fig 1.

2.4. Data collection

The survey was refined from previous Youth2000 series questions (https://www.fmhs.auckland.ac.nz/en/faculty/adolescent-health-research-group/publications-and-reports.html), validated measures and measures used in other surveys, as well as new questions developed from a rangatahi and Māori whanau photovoice and qualitative research process, and a digitally integrated survey process. Students completed the web-based survey on tablets in English or Te Reo Māori (the language of New Zealand's indigenous people). Questions appeared in text on the screen and were available via voice-over through headphones [6].

2.5. Regional estimates

Most estimates for this study are based on totals, means or proportions. To help simplify the exposition, we present the methods in the context of estimating totals. This is applicable to means and proportions since they are functions of totals. Initially, we have a population of size

Table 1.

	Not eligible schools	Eligible schools	Invited schools	Participating schools	Students in eligible schools	Students in invited schools	Students in participating schools	Students surveyed
**Total**	2362	169	86	49	130692	71105	42298	7721
**By education region**								
Tai Tokerau	462	25	10	10	97415	52013	31028	5545
Auckland	126	102	53	27	24074	14693	6871	1248
Waikato	238	42	23	12	9203	4399	4399	928
Other regions	1536	0	0	0	0	0	0	0
**By school type**								
Mainstream	2275	161	80	45	129765	70408	41828	7374
Kura	87	8	6	4	927	697	470	347

https://doi.org/10.1371/journal.pone.0251177.t001

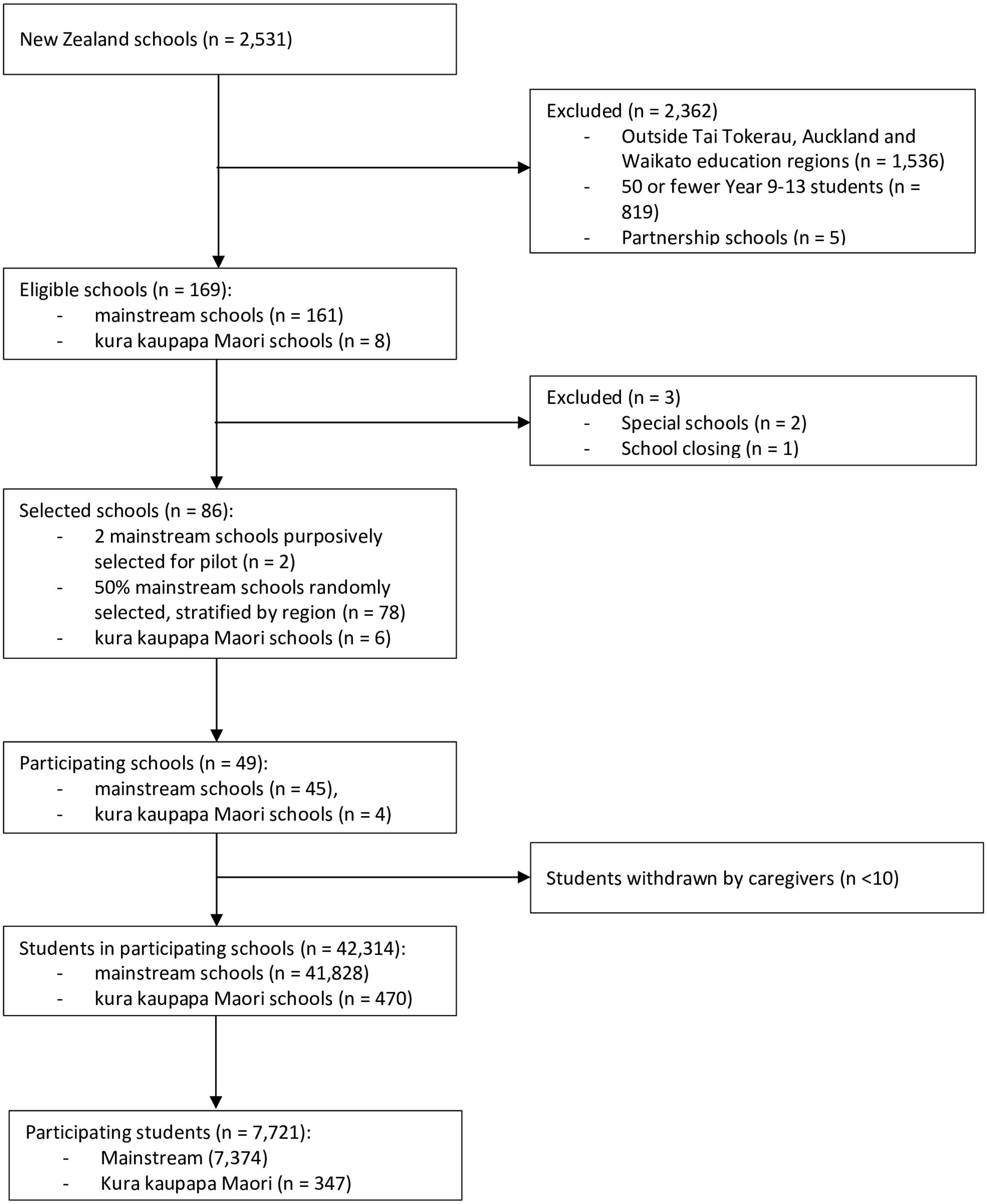

Fig 1. Sample design.

https://doi.org/10.1371/journal.pone.0251177.g001

N and we are interested in estimating the total of a variable of interest, called y, which can be written as $T_y = \sum_{i=1}^{N} y_i$. In the absence of complete data from all the population, T_y cannot be calculated. Consequently, this should be estimated. Since the sampling design is a stratified-multistage design, the estimator has to account for this design through weights [9–12]. The weighted estimator of T_y is

$$\hat{T}_y = \sum_{sample} w_i y_i,$$

where $w_i = 1/\pi_i$, and π_i is the sampling probability for individual i. The weight w_i can be interpreted as the number of people that individual i represents in the population. This type of estimator and its variances are available from the survey package in R.

2.6. Missing observations and extrapolation to the national population

The weighted estimator presented above accounts for the sampling scheme, but it has several drawbacks. First, it is unbiased, but only when there is not missing information. Second, it is known to be inefficient because it yields wider confidence intervals than other estimators of totals [13]. An approach to attaining more efficient estimators is to use auxiliary information available for the entire population (e.g. information from the sampling frame). For instance, for the Youth 2019 surveys, an option would be to use information on the ethnic distribution of students in the population. This information was not used to inform the sampling design, but we can use it post design to improve the estimators [14, 15].

Calibration is among these methods, it has been used in the literature when sampling weights are incorrect, to correct for non-response or to extrapolate to wider populations where there is compelling evidence that the factors contributing to the estimators are very similar in the target population and in the wider population [10, 16, 17]. The primary idea of calibration is to adjust the sampling weights w_i such that totals of known quantities are exactly estimated. To see this, let M denote the total number of Māori students in the population of interest. From the sampling frame, we know that this number is 24983 for the three regions in the study, and 59040 for the whole country. Although M is known, it is interesting to investigate what would be the estimator of M using only the survey data. That is $\hat{M} = \sum_{sample} w_i l_i$, where l_i is a binary variable denoting if the individual is Māori or not. Since M is actually known, one could always modify the sampling weights such that $\hat{M} = M$. The new weights ($\tilde{w}_i$) are found by minimizing a distance function between the original sampling weights and the modified weights subject to the constraint $\hat{M} = M$. The new weights are known as calibrated weights and the estimator is denoted $\tilde{M} = \sum_{sample} \tilde{w}_i l_i$. In theory, the variance of $\tilde{M}$ will never be larger than the variance of $\hat{M}$, which is based on the original weights. This calibration process can be done using several variables simultaneously. For example, the weights can be calibrated to demographic factors that are considered important in the analysis, and are available both for the sampling frame and the study population. Calibration can be implemented via the survey package in R with the function calibrate() [18, 19].

2.7. Calibrated estimates: Regional and national

We use calibrated weights at the regional level (Regions: Auckland, Tai Tokerau and Waikato) in order to improve the efficiency of our estimates, and adjust for differential non-response. In our case, we calibrate the regional weights to Regional totals of the demographic variables available from Education Counts: kura kaupapa Māori, School Deciles, Age, Gender and Ethnicity. The deciles are a measure of the socio-economic position of a school's student

community relative to other schools throughout the country. For example, decile 1 schools are the 10% of schools with the highest proportion of students from low socio-economic communities, whereas decile 10 schools are the 10% of schools with the lowest proportion of these students. A school's decile does not indicate the overall socio-economic mix of the school or reflect the quality of education the school provides. Deciles are used to provide funding to state and state-integrated schools to enable them to overcome the barriers to learning faced by students from lower socio-economic communities. The lower the school's decile, the more funding they receive [20].The majority of our outcome variables show a significant relationship to at least one of these demographic variables, this can be seen in the descriptive plots in S1 Statistics. Calibration invokes no assumptions apart from the study sample being a sample selected using a probabilistic design from a population of interest [16]. In our case, this assumption holds for regional estimates. However, for national estimates, the population of interest (national) is different to the population from where the sample was selected.

Our main goal is to generate national statistics that enable us to compare the results to previous national surveys. In order to do this, we have to assume that the regional sample is selected from the national population. This means that the distributions of factors contributing to the estimators are very similar in the Regional population (Regions: Auckland, Tai Tokerau and Waikato) and in the national population. In order to account for the demographic distribution of the national population, we calibrate these weights to the National totals of the same demographic variables used for the regional weights (kura kaupapa Māori, School Deciles, Age, Gender and Ethnicity). This calibration was done using the calibrate() function from the R package survey. The totals used for calibration are education counts available from https://www.education.govt.nz/our-work/contact-us/. In order to understand how different weights affect the estimation of outcomes of interest, we compared results for key health and wellbeing indicators (Tables 4 and 5).

2.8. Ethics

In each participating mainstream school, the principal or head of the board of trustees provided consent for the students to be invited to participate. Information for parents in English and Te Reo Māori was provided to the school (digitally and or printed) and made available to parents and caregivers who could opt to have their child excluded from the survey. Ethics approval was granted by the University of Auckland Human Subjects Ethics Committee (application #022244).

3. Results

3.1. Study participants

There were 2,531 schools nationally, and 624 in the Auckland, Tai Tokerau and Waikato regions. We excluded 819 schools from these regions because they had less than 50 year 9–13 students, and five because they were partnership schools. A further 2 were excluded mistakenly due to human error. This left 161 eligible mainstream schools in the three regions. Two large ethnically diverse schools were purposively selected for piloting, and 78 schools were randomly selected, making 80 (49.7%) in total. Of these, 45 (56.3%) agreed to participate. There were 41,828, students at participating mainstream schools, and 7,374 (59.7%) participated. The sampling design is shown in Fig 1.

There were 95 kura kaupapa Māori nationally, and 8 in the Auckland, Tai Tokerau and Waikato regions. Six were invited, and 4 (66.7%) agreed to participate. There were 470 students at participating kura, all of whom were invited to participate, and 347 (71%) participated. The rest of the results are presented for mainstream schools only.

The unweighted characteristics of participating mainstream schools and students are shown in Table 2, alongside comparable data for the previous national surveys, and all secondary school students in New Zealand. The participation rates were much lower than previous surveys, both for schools (56.3%) and for students (59.7%). In 16 schools, participation was under 50%, with a measles outbreak, teacher strikes, and high truancy rates indicated by school staff as likely to have affected response rates in their schools. Apart from this, illness, assessments and field-trips may have resulted in students being unable to participate. The majority of non-participating students did not arrive at the room in which the survey was taking place, and only 49 arrived at the room but declined to participate.

There are some important differences in the demographic characteristics of participating students compared to previous surveys, and to the national secondary school population distribution. There were a lower proportion of high decile schools included in the sample, but higher participation at high decile schools means that the 2019 sample population matches the national student population quite well in terms of school decile. There was a lower proportion of boys (45.1%) compared to the national student population, and to the surveys in 2001 and 2012, and a slightly higher proportion of students aged 17 and above than in previous surveys, though this is still lower than the national student population. There were ethnic differences too, with a much higher proportion of Asian students than previous surveys, and than the national student population, reflecting a higher proportion of the Asian population living in the Auckland region.

3.2. Estimates

Tables 3 and 4 display the actual regional and national totals and proportions for variables used to calibrate the sampling weights. This excludes kura kaupapa Māori schools because previous waves did not include such schools. We can observe in the confidence intervals that the variance yielded by calibrated weights is zero for these variables. This is due to the fact that we are calibrating to the actual totals, therefore the calibrated estimates should be exactly the same as the actual totals and in consequence there is no uncertainty (or variance). Fig 2 shows the distribution of calibrated and sampling weights. There is a significant shift (right skewed) in the distribution of calibrated weights. This happens because a large number of individuals are overrepresented by the original sampling weights. Thus, calibration decreases the magnitude of the weights of those individuals that are overrepresented, and increases the weights of individuals that are underrepresented.

Table 5 shows the estimates of the total student numbers for key health and wellbeing indicators, and Table 6 shows the estimated proportions. For the regional estimates, calibrated weights yield standard errors lower than those obtained with the unadjusted sampling weights. This leads to significantly narrower confidence intervals for all the variables in the analysis. We only present calibrated estimates of national quantities because the sampling design was a regional design and therefore, we do not have national level sampling weights. However, national calibration provides a tool to compare to previous surveys, where the sampling population was national.

4. Discussion

We have conducted a multistage cluster sample survey of New Zealand secondary school students from three regions, to build on three previous national surveys. We used calibrated inverse probability weighting in order to correct for demographic differences between the regional and national student populations and for non-response, which enables extrapolation of the results from the Youth 2019 survey to the whole secondary school population of New

Table 2. Unweighted characteristics of participating mainstream schools and students from previous surveys and national school data prior to the studies.

	Previous national surveys			Current survey in 3 regions	National data
	2001	2007	2012	2019* (Excluding kura kaupapa Māori)	2018
	n (%)	n (%)	n (%)	n (%)	n (%)
**Schools**					
Eligible[1]	389	389	397	161	407
Invited	133	115	125	80	-
Participated	114 (85.7)	96 (83.5)	91 (72.8)	45 (56.3)	-
**Decile**					
Low	23 (20.2)	15 (15.6)	26 (28.6)	13 (28.9)	98 (24.1)
Medium	51 (44.7)	52 (54.2)	36 (39.6)	21 (46.7)	177 (43.5)
High	40 (35.1)	29 (30.2)	29 (31.9)	10 (22.2)	125 (30.7)
Unknown	0 (0)	0 (0)	0 (0)	1 (2.2)	7 (1.7)
**Students**					
Eligible				41,828	280,163
Participated[2]	9,567 (74.0)	9,107 (74.0)	8,500 (68.0)	7,374 (59.7)	-
**Year**					
Year 9	2,457 (26.1)	2,176 (24.3)	2,061 (24.3)	1681 (23.1)	57,784 (20.8)
Year 10	2,233 (23.7)	2,090 (23.4)	1,936 (22.8)	1609 (22.1)	57,302 (20.6)
Year 11	2,156 (22.9)	1,933 (21.6)	1,727 (20.4)	1603 (22.1)	58,952 (21.2)
Year 12	1,580 (16.9)	1,669 (18.7)	1,534 (18.1)	1364 (18.8)	56,070 (20.1)
Year 13	978 (10.4)	1,077 (12.0)	1,227 (14.5)	1009 (13.9)	48,161 (17.3)
**Sex**					
Female	4,414 (46.1)	4,911 (54.0)	3,874 (45.6)	3990 (54.6)	140,862 (50.3)
Male	5,152 (53.9)	4,187 (46.0)	4,623 (54.4)	3321(45.4)	139,301 (49.7)
**Age**					
≤13	2,050 (21.5)	1,860 (20.4)	1,838 (21.7)	1338 (18.1)	47,191 (17.0)
14	2,285 (23.9)	2,101 (23.1)	1,896 (22.3)	1650 (22.4)	56,687 (20.5)
15	2,178 (22.8)	1,973 (21.7)	1,755 (20.7)	1631 (22.1)	56,900 (20.6)
16	1,725 (18.1)	1,743 (19.2)	1,578 (18.6)	1418 (19.2)	55,627 (20.1)
≥17	1,308 (13.7)	1,423 (15.6)	1,422 (16.8)	1337 (18.1)	60,417 (21.8)
**Ethnicity[3]**					
European	5,406 (57.4)	4,797 (52.8)	4,024 (47.4)	3067 (41.7)	145,487 (51.9)
Māori	2,340 (24.8)	1,702 (18.7)	1,705 (20.1)	1201(16.3)	58,119 (20.7)
Pacific	768 (8.2)	924 (10.2)	1,201 (14.1)	936 (12.7)	26,825 (9.6)
Asian	679 (7.2)	1,126 (12.4)	1,051 (12.4)	1776 (24.1)	32,739 (11.7)
Other	230 (2.4)	531 (5.8)	511 (6.0)	381 (5.2)	16,993 (6.1)
**NZDep[4]**					
Low deprivation	-	3,218 (36.3)	2,718 (32.4)	2,105 (28.6)	-
Medium	-	3,397 (38.3)	3,001 (35.8)	2,783 (37.7)	-
High deprivation	-	2,250 (25.4)	2,674 (31.9)	1,845 (25.0)	-
**NA**				641 (8.7)	
**Decile**					
Low	1,732 (18.1)	3,218 (35.3)	1,793 (21.1)	1,203 (16.3)	46,716 (16.7)
Medium	4,393 (45.9)	3,397 (37.3)	3,296 (38.8)	3,242 (44.0)	121,810 (43.5)
High	3,445 (36.0)	2,250 (24.7)	3,411 (40.1)	2,887 (39.2)	104,987 (37.5)

(*Continued*)

Table 2. (Continued)

	Previous national surveys			Current survey in 3 regions	National data
	2001	2007	2012	2019* (Excluding kura kaupapa Māori)	2018
	n (%)	n (%)	n (%)	n (%)	n (%)
Unknown	0 (0)	242 (2.7)	0 (0)	42 (0.6)	6,650 (2.4)

[1] Number of schools with more than 50 students in years 9–13

[2] Totals for each variable (not shown) are different to the overall total number of participating students due to different numbers of missing data for each.

[3] Ethnicity was assigned on the basis of prioritised ethnicity, using the NZ Census ethnicity prioritisation method [21]

[4] New Zealand Deprivation Index scores based on census areas [22], combined to form 3 categories

https://doi.org/10.1371/journal.pone.0251177.t002

Table 3. National and regional student population estimates- comparison of actual quantities, estimates using sampling weights and estimates using calibrated weights.

	TOTALS				
DEMOGRAPHIC CHARACTERISTICS	*Actual Total Regional*	Sampling Weights Regional	Calibrated Weights Regional	*Actual Total National*	Calibrated Weights National
**DECILE1**	11839	7321 [-45,14688]	11839 [11839,11839]	14473	14473 [14473,14473]
**DECILE2**	8471	6187 [-2501,14876]	8471 [8471,8471]	12860	12860 [12860,12860]
**DECILE4**	14212	13701 [1033,26369]	14212 [14212,14212]	25958	25958 [25958,25958]
**DECILE3**	9175	7920 [-851,16690]	9175 [9175,9175]	20131	20131 [20131,20131]
**DECILE5**	10379	7554 [-2062,17169]	10379 [10379,10379]	21409	21409 [21409,21409]
**DECILE6**	10145	4978 [-811,10768]	10145 [10145,10145]	38793	38793 [38793,38793]
**DECILE7**	17084	27648 [5303,49994]	17084 [17084,17084]	35650	35650 [35650,35650]
**DECILE8**	8604	3000 [-2714,8715]	8604 [8604,8604]	33650	33650 [33650,33650]
**DECILE9**	22554	35989 [3607,68372]	22554 [22554,22554]	38736	38736 [38736,38736]
**AGE13ANDUNDER**	22241	23522 [17565,29479]	22241 [22241,22241]	47361	47361 [47361,47361]
**AGE14**	26685	29307 [22100,36514]	26685 [26685,26685]	56843	56843 [56843,56843]
**AGE15**	26527	29099 [22001,36197]	26527 [26527,26527]	57060	57060 [57060,57060]
**AGE16**	26181	24892 [18471,31313]	26181 [26181,26181]	55744	55744 [55744,55744]
**GENDER FEMALE**	64737	72549 [52895,92203]	64737 [64737,64737]	139694	139694 [139694,139694]
**MĀORI**	24393	20384 [15844,24924]	24393 [24393,24393]	58866	58866 [58866,58866]
**EUROPEAN**	53889	54902 [37045,72760]	53889 [53889,53889]	145487	145487 [145487,145487]
**PACIFIC**	19913	16418 [10311,22525]	19913 [19913,19913]	26826	26826 [26826,26826]
**ASIAN**	22358	31858 [19382,44335]	22358 [22358,22358]	32739	32739 [32739,32739]

https://doi.org/10.1371/journal.pone.0251177.t003

Table 4. National and regional estimates- comparison of actual quantities, estimates using sampling weights and estimates using calibrated weights.

PROPORTIONS					
	Actual Total Regional	Sampling Weights Regional	Calibrated Weights Regional	*Actual Total National*	Calibrated Weights National
**DECILE1**	0.09	0.056 [-0.004,0.116]	0.091 [0.091,0.091]	0.051	0.052 [0.052,0.052]
**DECILE2**	0.065	0.047 [-0.02,0.115]	0.065 [0.065,0.065]	0.046	0.046 [0.046,0.046]
**DECILE4**	0.108	0.105 [0.007,0.203]	0.109 [0.109,0.109]	0.092	0.092 [0.092,0.092]
**DECILE3**	0.07	0.061 [-0.009,0.13]	0.07 [0.07,0.07]	0.071	0.072 [0.072,0.072]
**DECILE5**	0.079	0.058 [-0.016,0.132]	0.079 [0.079,0.079]	0.076	0.076 [0.076,0.076]
**DECILE6**	0.077	0.038 [-0.007,0.083]	0.078 [0.078,0.078]	0.138	0.138 [0.138,0.138]
**DECILE7**	0.13	0.212 [0.049,0.374]	0.131 [0.131,0.131]	0.126	0.127 [0.127,0.127]
**DECILE8**	0.066	0.023 [-0.021,0.067]	0.066 [0.066,0.066]	0.119	0.12 [0.12,0.12]
**DECILE9**	0.172	0.276 [0.069,0.482]	0.173 [0.173,0.173]	0.137	0.138 [0.138,0.138]
**AGE13 AND UNDER**	0.169	0.18 [0.164,0.196]	0.17 [0.17,0.17]	0.168	0.169 [0.169,0.169]
**AGE14**	0.203	0.224 [0.207,0.242]	0.204 [0.204,0.204]	0.202	0.202 [0.202,0.202]
**AGE15**	0.202	0.223 [0.211,0.234]	0.203 [0.203,0.203]	0.202	0.203 [0.203,0.203]
**AGE16**	0.2	0.191 [0.174,0.207]	0.2 [0.2,0.2]	0.198	0.198 [0.198,0.198]
**GENDER FEMALE**	0.493	0.555 [0.424,0.687]	0.496 [0.496,0.496]	0.496	0.497 [0.497,0.497]
**MĀORI**	0.186	0.156 [0.125,0.188]	0.187 [0.187,0.187]	0.209	0.21 [0.21,0.21]
**EUROPEAN**	0.411	0.42 [0.348,0.492]	0.413 [0.413,0.413]	0.516	0.518 [0.518,0.518]
**PACIFIC**	0.152	0.126 [0.078,0.173]	0.152 [0.152,0.152]	0.095	0.095 [0.095,0.095]
**ASIAN**	0.17	0.244 [0.183,0.305]	0.171 [0.171,0.171]	0.116	0.117 [0.117,0.117]

https://doi.org/10.1371/journal.pone.0251177.t004

Zealand. The original sampling design was only representative of the three main regions in New Zealand (Tai Tokerau, Auckland and Waikato). These three regions are believed to represent the diversity of the New Zealand population [17], however using these data to make inferences about the national situation is imprecise.

One of the main goals of this paper is to improve the estimates at the regional level using calibrated weights. Calibration aims to account for oversampling or non-response of some groups of individuals. An interesting example is the proportion of individuals suffering depressive symptoms. Using the original sampling weights, this proportion is estimated to be 0.25 (0.224,0.275), while national calibration yields a lower estimate of 0.227 (0.216,0.239). A reason for this is that the original sample could have oversampled individuals more prone to suffer such symptoms, including a higher proportion of girls and a higher proportion of Pacific and Asian students. Another example is the proportion of individuals who reported binge drinking in the last 4 weeks. This proportion is estimated as 0.177 (0.155,0.199) using sampling weights,

Table 5. Estimates of total student numbers for health and wellbeing indicators.

TOTALS					
	Unweighted	Sampling Weights Regional	Calibrated Weights Regional	Calibrated Weights National	n
MOVED HOME TWO OR MORE TIMES IN LAST YEAR	526	9346 [7533,11159]	10426 [9451,11402]	21632 [19153,24111]	7311
PARENTS WORRY ABOUT NOT HAVING ENOUGH MONEY FOR FOOD (OFTEN, ALL THE TIME)	896	15976 [12514,19438]	17571 [16148,18993]	33242 [30122,36362]	7311
SPENDING ENOUGH TIME WITH AT LEAST ONE PARENT (MOSTLY)	5130	91642 [69496,113788]	92278 [91346,93210]	199440 [197157,201723]	7311
FAMILY WANTS TO KNOW WHO YOU'RE WITH AND WHERE YOU ARE (USUALLY OR ALWAYS)	6681	119420 [90995,147845]	118686 [117509,119863]	255696 [252310,259081]	7311
ADULTS AT SCHOOL CARE (A LOT)	5653	101015 [77690,124340]	101383 [100040,102727]	217973 [215324,220622]	7218
HAVE AN ADULT FEEL OK TALKING TO OUTSIDE FAMILY	3216	57512 [43342,71681]	58427 [56540,60315]	130051 [125174,134929]	7311
FEEL SAFE IN OWN NEIGHBOURHOOD (ALWAYS)†	4007	71221 [51788,90654]	70452 [68657,72248]	155142 [150947,159337]	7311
BULLIED AT SCHOOL (AT LEAST WEEKLY)	383	6781 [4999,8562]	7024 [6221,7827]	16529 [13977,19081]	7163
WITNESSED ADULTS AT HOME HIT OR PHYSICALLY HURT EACH OTHER	405	7254 [5597,8911]	7669 [7106,8231]	15971 [14651,17292]	6809
SEXUAL ABUSE	820	14740 [11643,17837]	14996 [13976,16015]	32977 [30857,35097]	6822
RATED GENERAL HEALTH AS FAIR OR POOR	4749	84841 [63128,106554]	85404 [83932,86875]	188209 [184259,192158]	7311
CLINICALLY SIGNIFICANT DEPRESSIVE SYMPTOMS (RADS-SF SCORE ≥28)	1727	31281 [24447,38114]	29596 [28227,30966]	61184 [57997,64372]	7014
ATTEMPTED SUICIDE IN LAST 12 MONTHS	439	7934 [6275,9593]	8626 [7856,9397]	17105 [15224,18987]	7048
AT LEAST MONTHLY CIGARETTE USE	262	4621 [3533,5708]	5595 [5010,6181]	12760 [11175,14344]	6850
AT LEAST ONE EPISODE OF BINGE DRINKING IN LAST 4 WEEKS	1221	21395 [14913,27877]	23678 [21963,25393]	57009 [53164,60853]	6775
AT LEAST MONTHLY MARIJUANA USE	712	12425 [9557,15293]	14236 [13248,15225]	32905 [30311,35499]	7311
EVER HAD SEXUAL INTERCOURSE	1180	20877 [15484,26270]	24159 [22947,25371]	54767 [52081,57453]	6907
USED A CONDOM AT LAST SEXUAL INTERCOURSE *	695	12353 [8685,16021]	13803 [12936,14671]	31655 [29317,33993]	6836
EVER BEEN PREGNANT OR GOT SOMEONE PREGNANT	60	1054 [644,1465]	1261 [989,1532]	2564 [1830,3299]	1135
PHYSICAL ACTIVITY FOR AT LEAST 60 MIN EVERY DAY IN LAST WEEK	1065	18948 [13766,24131]	20275 [19394,21155]	44005 [42054,45955]	6976
ALWAYS WEAR A SEATBELT WHEN DRIVING/BEING DRIVEN IN A CAR	5231	93864 [69473,118254]	91480 [89825,93134]	202405 [197714,207095]	6981
PASSENGER IN A CAR DRIVEN BY A RISKY DRIVER IN THE LAST MONTH	1791	31409 [22879,39939]	32869 [30828,34911]	73403 [68963,77843]	6638
DRIVER ENGAGING IN RISKY DRIVING IN THE LAST MONTH	194	3379 [1890,4869]	3705 [2755,4655]	7595 [5545,9645]	1600
ACCESSED HEALTH CARE IN LAST 12 MONTHS	5493	98371 [73250,123492]	97292 [95461,99122]	212253 [208725,215781]	7081
WANTED TO SEE A HEALTH PROVIDER BUT WEREN'T ABLE TO	1448	25807 [20341,31274]	26467 [25242,27692]	54682 [51490,57874]	7061

*Proportion is out of those who have had sex

https://doi.org/10.1371/journal.pone.0251177.t005

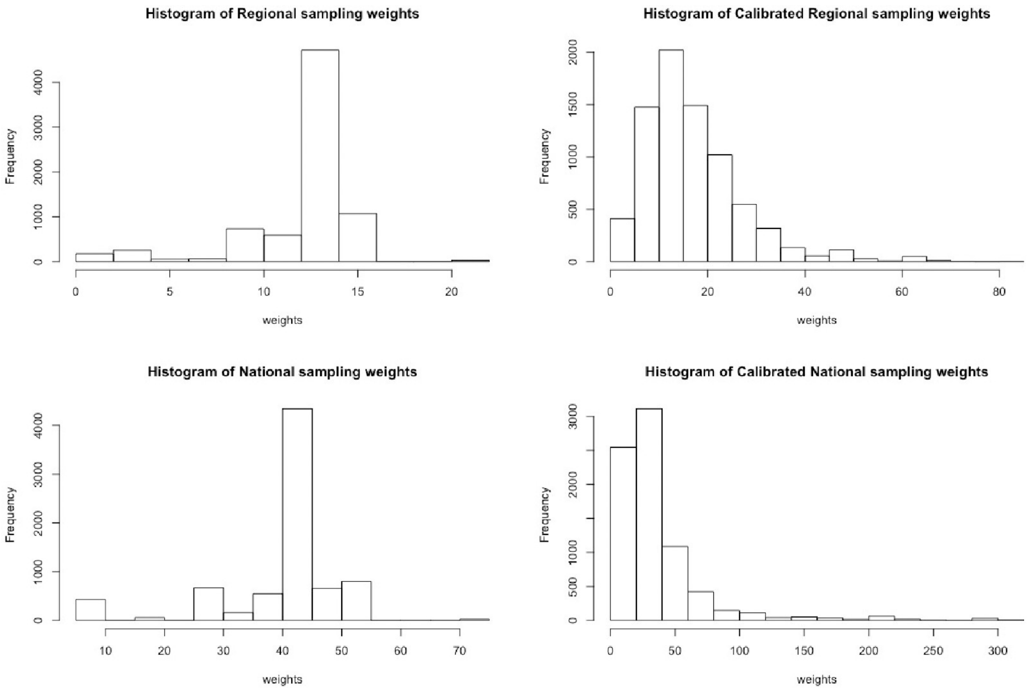

Fig 2. Distribution of regional and national sampling weights and calibrated weights.

https://doi.org/10.1371/journal.pone.0251177.g002

while it is estimated to be 0.219 (0.204,0.234) using nationally calibrated weights. The original sample could have oversampled individuals that were less likely to engage in binge drinking, with the higher proportion of girls, Pacific and Asian students representing groups who engage in binge drinking less.

A question that arises is what estimators are more reliable. In such case we recommend the use of calibrated estimators as they yield confidence intervals that are significantly narrower than those obtained using the original sampling weights. This is a well-known property of calibration since it reduces the uncertainty in the sample by incorporating information known prior to the study [19, 23, 24].

An additional goal of this paper was to use the regional sample to make inferences about the whole adolescent population of New Zealand. This is particularly important because previous Youth2000 surveys were designed using a national sampling frame instead of a regional sampling frame. The 2019 survey was designed using a 3-region sampling frame for logistical and financial reasons. There is ongoing interest in comparing the results and trends with previous national surveys. To achieve this, we calibrated our regional sampling weights to represent the national population based on some of the demographic factors presented in Table 2. The calibrated estimates presented in Table 4 show some differences between the regional and national proportions.

There are few nationally-representative data available for health and wellbeing indicators among New Zealand youth, apart from the Youth 2000 surveys. The ASH Year 10 Snapshot survey reported that 5.9% of Year 10 students are regular smokers. Our data shows that 4.8% of all secondary school students are regular smokers, but this includes younger students who are less likely to smoke. The NZ Health Survey estimated that 78.9% of adults over 15 years visited their GP in the last 12-months, which compares with our estimate of 78.1%. Likewise, the NZ Health Survey estimated that 20.6% of adults over 15 years had an unmet need for

Table 6. Health and wellbeing indicators.

		PROPORTIONS			
	Unweighted	Sampling Weights Regional	Calibrated Weights Regional	Calibrated Weights National	n
MOVED HOME TWO OR MORE TIMES IN LAST YEAR	0.072	0.072 [0.059,0.084]	0.08 [0.072,0.087]	0.077 [0.068,0.086]	7311
PARENTS WORRY ABOUT NOT HAVING ENOUGH MONEY FOR FOOD (OFTEN, ALL THE TIME)	0.123	0.122 [0.098,0.147]	0.135 [0.124,0.145]	0.118 [0.107,0.129]	7311
SPENDING ENOUGH TIME WITH AT LEAST ONE PARENT (MOSTLY)	0.702	0.702 [0.692,0.711]	0.706 [0.699,0.714]	0.71 [0.702,0.718]	7311
FAMILY WANTS TO KNOW WHO YOU'RE WITH AND WHERE YOU ARE (USUALLY OR ALWAYS)	0.914	0.914 [0.905,0.923]	0.909 [0.9,0.918]	0.91 [0.898,0.922]	7311
ADULTS AT SCHOOL CARE (A LOT)	0.783	0.783 [0.763,0.804]	0.787 [0.777,0.798]	0.787 [0.777,0.797]	7218
HAVE AN ADULT FEEL OK TALKING TO OUTSIDE FAMILY	0.44	0.44 [0.421,0.46]	0.447 [0.433,0.462]	0.463 [0.446,0.48]	7311
FEEL SAFE IN OWN NEIGHBOURHOOD (ALWAYS)†	0.548	0.545 [0.51,0.581]	0.539 [0.526,0.553]	0.552 [0.537,0.567]	7311
BULLIED AT SCHOOL (AT LEAST WEEKLY)	0.053	0.053 [0.046,0.06]	0.055 [0.049,0.061]	0.06 [0.051,0.07]	7163
WITNESSED ADULTS AT HOME HIT OR PHYSICALLY HURT EACH OTHER	0.059	0.06 [0.05,0.069]	0.064 [0.059,0.069]	0.061 [0.056,0.066]	6809
SEXUAL ABUSE	0.12	0.121 [0.104,0.138]	0.125 [0.116,0.133]	0.126 [0.118,0.134]	6822
RATED GENERAL HEALTH AS FAIR OR POOR	0.65	0.65 [0.63,0.669]	0.654 [0.643,0.665]	0.67 [0.656,0.684]	7311
CLINICALLY SIGNIFICANT DEPRESSIVE SYMPTOMS (RADS-SF SCORE ≥28)	0.246	0.25 [0.224,0.275]	0.238 [0.227,0.249]	0.227 [0.216,0.239]	7014
ATTEMPTED SUICIDE IN LAST 12 MONTHS	0.062	0.063 [0.049,0.078]	0.069 [0.063,0.075]	0.063 [0.056,0.07]	7048
AT LEAST MONTHLY CIGARETTE USE	0.038	0.038 [0.03,0.046]	0.046 [0.041,0.051]	0.048 [0.043,0.054]	6850
AT LEAST ONE EPISODE OF BINGE DRINKING IN LAST 4 WEEKS	0.18	0.177 [0.155,0.199]	0.197 [0.183,0.212]	0.219 [0.204,0.234]	6775
AT LEAST MONTHLY MARIJUANA USE	0.097	0.095 [0.086,0.105]	0.109 [0.101,0.117]	0.117 [0.108,0.126]	7311
EVER HAD SEXUAL INTERCOURSE	0.171	0.169 [0.152,0.186]	0.197 [0.187,0.208]	0.206 [0.196,0.217]	6907
USED A CONDOM AT LAST SEXUAL INTERCOURSE *	0.627	0.629 [0.591,0.667]	0.613 [0.589,0.637]	0.617 [0.59,0.644]	6836
EVER BEEN PREGNANT OR GOT SOMEONE PREGNANT	0.053	0.053 [0.038,0.067]	0.054 [0.043,0.066]	0.049 [0.035,0.063]	1135
PHYSICAL ACTIVITY FOR AT LEAST 60 MIN EVERY DAY IN LAST WEEK	0.153	0.152 [0.137,0.167]	0.164 [0.157,0.171]	0.164 [0.157,0.172]	6976
ALWAYS WEAR A SEATBELT WHEN DRIVING/BEING DRIVEN IN A CAR	0.749	0.753 [0.73,0.777]	0.741 [0.729,0.754]	0.756 [0.74,0.772]	6981
PASSENGER IN A CAR DRIVEN BY A RISKY DRIVER IN THE LAST MONTH	0.27	0.265 [0.244,0.286]	0.282 [0.266,0.297]	0.289 [0.273,0.305]	6638
DRIVER ENGAGING IN RISKY DRIVING IN THE LAST MONTH	0.121	0.118 [0.1,0.135]	0.127 [0.114,0.14]	0.132 [0.116,0.147]	1600
ACCESSED HEALTH CARE IN LAST 12 MONTHS**	0.776	0.778 [0.76,0.795]	0.774 [0.762,0.786]	0.781 [0.77,0.792]	7081
WANTED TO SEE A HEALTH PROVIDER BUT WEREN'T ABLE TO***	0.205	0.205 [0.189,0.22]	0.211 [0.202,0.22]	0.202 [0.191,0.213]	7061

*Taken from those who have ever had sex.

https://doi.org/10.1371/journal.pone.0251177.t006

healthcare, and our data estimates this to be 20.2% [25, 26] These results highlight that calibration methods can improve the precision of national estimates when compared to similar surveys, however it should be noted that calibration methods cannot account for factors outside of demographic features (i.e. unique regional differences) and therefore should be utilised with this limitation in mind.

Future research will involve calibration of the previous surveys using a similar approach to reduce bias in the estimates, as well as investigating how different designs can improve the results and methods for combining the periodic complex surveys done in the years 2001, 2007, 2012 and 2019.

Supporting information

S1 File.
(ZIP)

S1 Statistics.
(PDF)

Author Contributions

Conceptualization: T. Fleming, S. Lewycka.

Data curation: C. Rivera-Rodriguez, D. Archer.

Formal analysis: C. Rivera-Rodriguez.

Funding acquisition: T. C. Clark, T. Fleming.

Investigation: T. C. Clark, T. Fleming, S. Crengle, R. Peiris-John.

Methodology: C. Rivera-Rodriguez.

Project administration: T. C. Clark, D. Archer.

Resources: T. Fleming, D. Archer.

Software: C. Rivera-Rodriguez, D. Archer.

Supervision: T. Fleming, S. Lewycka.

Validation: D. Archer, S. Lewycka.

Visualization: S. Lewycka.

Writing – original draft: C. Rivera-Rodriguez, S. Lewycka.

Writing – review & editing: T. C. Clark, T. Fleming, D. Archer, S. Crengle, R. Peiris-John, S. Lewycka.

References

1. Clark T, Fleming T, Bullen P, Crengle S, Denny S, et al. (2013) Health and well-being of secondary school students in New Zealand: trends between 2001, 2007 and 2012. J Paediatr Child Health 49: 925–934. https://doi.org/10.1111/jpc.12427 PMID: 24251658
2. Lewycka S, Clark T, Peiris-John R, Fenaughty J, Bullen P, et al. (2018) Downwards trends in adolescent risk-taking behaviours in New Zealand: Exploring driving forces for change. J Paediatr Child Health 54: 602–608. https://doi.org/10.1111/jpc.13930 PMID: 29779222
3. Bor W, Dean AJ, Najman J, Hayatbakhsh R (2014) Are child and adolescent mental health problems increasing in the 21st century? A systematic review. Aust N Z J Psychiatry 48: 606–616. https://doi.org/10.1177/0004867414533834 PMID: 24829198

4. Soneji S, Barrington-Trimis JL, Wills TA, Leventhal AM, Unger JB, et al. (2017) Association Between Initial Use of e-Cigarettes and Subsequent Cigarette Smoking Among Adolescents and Young Adults: A Systematic Review and Meta-analysis. JAMA Pediatr 171: 788–797. https://doi.org/10.1001/jamapediatrics.2017.1488 PMID: 28654986
5. Clark TC, Le Grice J, Moselen E, Fleming T, Crengle S, et al. (2018) Health and wellbeing of Māori secondary school students in New Zealand: Trends between 2001, 2007 and 2012. Aust N Z J Public Health 42: 553–561. https://doi.org/10.1111/1753-6405.12839 PMID: 30370961
6. Fleming T, Peiris-John R, Crengle S, Archer D, Sutcliffe K, et al. (2020) Youth19 Rangatahi Smart Survey, Initial Findings:Introduction and Methods. The Youth19 Research Group. The University of Auckland and Victoria University of Wellington, New Zealand.
7. Morton SMB, Ramke J, Kinloch J, Grant CC, Carr PA, et al. (2015) Growing Up in New Zealand cohort alignment with all New Zealand births. Aust N Z J Public Health 39: 82–87. https://doi.org/10.1111/1753-6405.12220 PMID: 25168836
8. Education Counts: School Rolls (n.d.). Available: https://www.educationcounts.govt.nz/statistics/schooling/student-numbers/6028. Accessed 22 July 2020.
9. Deville J-C, Särndal C-E (1992) Calibration estimators in survey sampling. J Am Stat Assoc 87: 376–382. https://doi.org/10.1080/01621459.1992.10475217
10. Sarndal CE, Swensson B, Wretman J (1992) Model Assisted Survey Sampling. New York.
11. Fuller WA (2009) Sampling Statistics. Hoboken, NJ, USA: John Wiley & Sons, Inc. https://doi.org/10.1002/9780470523551
12. Särndal C-E, Lundström S (2005) Introduction to estimation in the presence of nonresponse. Estimation in Surveys with Nonresponse. Chichester, UK: John Wiley & Sons, Ltd. pp. 43–48. https://doi.org/10.1002/0470011351.ch5
13. Breslow NE, Holubkov R (1997) Weighted likelihood, pseudo-likelihood and maximum likelihood methods for logistic regression analysis of two-stage data. Stat Med 16: 103–116. https://doi.org/10.1002/(sici)1097-0258(19970115)16:1<103::aid-sim474>3.0.co;2-p PMID: 9004386
14. Spiegelman D, Rivera-Rodriguez CL, Haneuse S (2016) Evaluating Public Health Interventions: 3. The Two-Stage Design for Confounding Bias Reduction-Having Your Cake and Eating It Two. Am J Public Health 106: 1223–1226. https://doi.org/10.2105/AJPH.2016.303250 PMID: 27285260
15. Breslow NE, Amorim G, Pettinger MB, Rossouw J (2013) Using the Whole Cohort in the Analysis of Case-Control Data: Application to the Women's Health Initiative. Stat Biosci 5. https://doi.org/10.1007/s12561-013-9080-2 PMID: 24363785
16. Deville JC, Sarndal CE (1992) Calibration estimators in survey sampling. J Am Stat Assoc 87: 376–382.
17. Fuller W (2009) Sampling Statistics. John Wiley and Sons.
18. Lumley T, Shaw PA, Dai JY (2011) Connections between survey calibration estimators and semiparametric models for incomplete data. Int Stat Rev 79: 200–220. https://doi.org/10.1111/j.1751-5823.2011.00138.x PMID: 23833390
19. Breslow NE, Lumley T, Ballantyne CM, Chambless LE, Kulich M (2009) Improved Horvitz-Thompson Estimation of Model Parameters from Two-phase Stratified Samples: Applications in Epidemiology. Stat Biosci 1: 32. https://doi.org/10.1007/s12561-009-9001-6 PMID: 20174455
20. School Deciles (2021). New Zealand Ministry of Education. Available: https://www.education.govt.nz/school/funding-and-financials/resourcing/operational-funding/school-decile-ratings/. Accessed 23 March 2021.
21. Statistics New Zealand (2005) Statistical standard for ethnicity.
22. Salmond C, Crampton P, Sutton F, Atkinson J (2006) NZDep2006 Census Area Unit Data. Available: http://www.wnmeds.ac.nz/academic/dph/research/socialindicators.html.
23. Rivera-Rodriguez C, Toscano C, Resch S (2019) Improved calibration estimators for the total cost of health programs and application to immunization in Brazil. PLoS One 14: e0212401. https://doi.org/10.1371/journal.pone.0212401 PMID: 30840645
24. Breslow NE, Lumley T, Ballantyne CM, Chambless LE, Kulich M (2009) Using the whole cohort in the analysis of case-cohort data. Am J Epidemiol 169: 1398–1405. https://doi.org/10.1093/aje/kwp055 PMID: 19357328
25. NZHS (2018) Regional Data Explorer. New Zealand Health Survey: Regional Data Explorer. Available: https://minhealthnz.shinyapps.io/nz-health-survey-2014-17-regional-update/. Accessed 25 March 2021.
26. ASH (2019) 2019 ASH Year 10 Snapshot: Topline Results–Smoking. Action on Smoking and Health.
